# miRNA signatures associated with vulnerability to food addiction in mice and humans

**DOI:** 10.1172/JCI156281

**Published:** 2022-05-16

**Authors:** Alejandra García-Blanco, Laura Domingo-Rodriguez, Judit Cabana-Domínguez, Noèlia Fernández-Castillo, Laura Pineda-Cirera, Jordi Mayneris-Perxachs, Aurelijus Burokas, Jose Espinosa-Carrasco, Silvia Arboleya, Jessica Latorre, Catherine Stanton, Bru Cormand, Jose-Manuel Fernández-Real, Elena Martín-García, Rafael Maldonado

**Affiliations:** 1 Laboratory of Neuropharmacology-Neurophar, Department of Medicine and Life Sciences, Universitat Pompeu Fabra (UPF), Barcelona, Spain.; 2 Departament de Genètica, Microbiologia i Estadística, Facultat de Biologia, Universitat de Barcelona, Barcelona, Catalonia, Spain.; 3 Centro de Investigación Biomédica en Red de Enfermedades Raras (CIBERER), Valencia, Spain.; 4 Institut de Biomedicina de la Universitat de Barcelona (IBUB), Barcelona, Catalonia, Spain.; 5 Institut de Recerca Sant Joan de Déu (IR-SJD), Esplugues de Llobregat, Barcelona, Catalonia, Spain.; 6 Nutrition, Eumetabolism and Health Group, Girona Biomedical Research Institute (IdibGi), Girona, Spain.; 7 CIBER Fisiopatología de la Obesidad y Nutrición (CIBEROBN), Girona, Spain.; 8 Department of Diabetes, Endocrinology and Nutrition, Dr. Josep Trueta University Hospital, Girona, Spain.; 9 Department of Biological Models, Institute of Biochemistry, Life Sciences Center, Vilnius University, Vilnius, Lithuania.; 10 Centre for Genomic Regulation (CRG), The Barcelona Institute of Science and Technology, Barcelona, Catalonia, Spain.; 11 APC Microbiome Institute, University College Cork, Cork, Ireland.; 12 Teagasc Food Research Centre, Moorepark, Fermoy, Ireland.; 13 Deparment of Medical Sciences, Faculty of Medicine, University of Girona, Girona, Spain.; 14 Hospital del Mar Medical Research Institute (IMIM), Barcelona, Catalonia, Spain.

**Keywords:** Neuroscience, Addiction, Epigenetics

## Abstract

Food addiction is characterized by a loss of behavioral control over food intake and is associated with obesity and other eating disorders. The mechanisms underlying this behavioral disorder are largely unknown. We aimed to investigate the changes in miRNA expression promoted by food addiction in animals and humans and their involvement in the mechanisms underlying the behavioral hallmarks of this disorder. We found sharp similitudes between miRNA signatures in the medial prefrontal cortex (mPFC) of our animal cohort and circulating miRNA levels in our human cohort, which allowed us to identify several miRNAs of potential interest in the development of this disorder. Tough decoy (TuD) inhibition of miRNA-29c-3p in the mouse mPFC promoted persistence of the response and enhanced vulnerability to developing food addiction, whereas miRNA-665-3p inhibition promoted compulsion-like behavior and also enhanced food addiction vulnerability. In contrast, we found that miRNA-137-3p inhibition in the mPFC did not lead to the development of food addiction. Therefore, miRNA-29c-3p and miRNA-665-3p could be acting as protective factors with regard to food addiction. We believe the elucidation of these epigenetic mechanisms will lead to advances toward identifying innovative biomarkers and possible future interventions for food addiction and related disorders based on the strategies now available to modify miRNA activity and expression.

## Graphical abstract

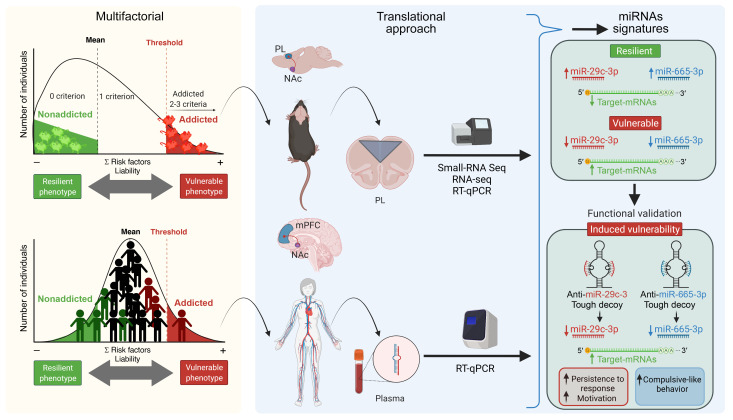

## Introduction

Food addiction is a multifactorial, complex disorder characterized by loss of control over food intake and has increased in prevalence in recent years (1), particularly during the current COVID-19 pandemic (2). This behavioral alteration is related to obesity and eating disorders and lacks effective treatments, leading to high socioeconomic costs worldwide. Despite the early definition of this concept (3), the 5th edition of the *Diagnostic and Statistical Manual of Mental Disorders* (DSM-5) does not include the construct of food addiction, given its controversy and lack of strong scientific evidence. However, a widely accepted tool is currently used in the clinic to measure food addiction: the Yale Food Addiction Scale (YFAS) (4), and a new YFAS 2.0 has been recently designed to apply the DSM-5 criteria for substance use disorder to eating behaviors. The YFAS 2.0 food addiction criteria can be summarized in 3 behavioral hallmarks also used in rodent models to mimic this disorder: persistent food seeking, high motivation to obtain food, and compulsion-like behavior (5). Studies in animals and humans have discovered important similitudes in the neurobiological substrate underlying substance use disorders and food addiction. Indeed, animal studies revealed similar brain networks involved in both disorders (6–10), whereas human neuroimaging studies also showed similar neuroadaptations within the reward circuits in the mesocorticolimbic system in both groups of patients (11, 12).

Eating behavior is controlled by complex networks of signals leading to homeostatic mechanisms that promote intake directly depending on energy requirements and an allostatic control that stimulates food intake independently of energy needs to accumulate energy for possible deficits of food supplies (13). The hypothalamus mainly controls homeostatic mechanisms within the central nervous system (13), whereas the main pathway for allostatic eating control is the mesocorticolimbic system (6, 8, 10). Notably, the corticostriatal pathway regulates the reward circuitry, and the prefrontal cortex (PFC) is responsible for behavioral self-control (7, 14). Therefore, we focused our study on the epigenetic signatures of food addiction in the medial prefrontal cortex (mPFC), since we have recently pointed out the crucial role of this cortical area in the development of addiction-like eating behavior in mice (8). Indeed, chemogenetic inhibition of the glutamatergic projections of the prelimbic (PL) area of the mPFC enhances food addiction vulnerability in mice (8). Furthermore, modulation of the endocannabinoid and dopaminergic signaling systems in this pathway confers vulnerability to food and cocaine addiction–like behavior (15). In agreement, synaptic deficits in the PL to nucleus accumbens (NAc) core projections have been associated with addiction-like behavior toward highly palatable food (7). Consistently, mPFC hypoactivity has been associated with obesity and drug addiction in neuroimaging studies in humans (14), further supporting interest in the mPFC as the target area to investigate the epigenetic signatures of food addiction.

The interactions between genes and the environment seem crucial in the vulnerability to developing food addiction. Epigenetics is essential in the interplay between these 2 factors to understand how the environment controls gene function changes without modifying the gene sequence. Epigenetic mechanisms are mainly mediated by DNA methylation, histone modification, and miRNAs (16, 17). miRNAs are small, noncoding RNA molecules that regulate gene expression by binding to target mRNAs to inhibit translation or promote mRNA degradation. Each miRNA can regulate up to hundreds of downstream targets, and every mRNA can be targeted by several miRNAs, creating a dynamic system that allows the cells to fine-tune gene expression (17). miRNAs play a crucial role in metabolic alterations leading to obesity (16) and in several psychiatric disorders and physiopathological processes related to drug addiction, such as reward, synaptic plasticity, learning, withdrawal, and relapse (18–20). However, the role of miRNAs in the development of food addiction has not yet been investigated. Considering the close overlap between drug and food addiction and the relationships between food addiction and obesity, miRNAs could also play a crucial role as key mediators between environments and genetic vulnerability in the development of these disorders (21). Furthermore, miRNAs could represent potential biomarkers of interest for the early detection of physiopathological alterations that may lead to food addiction.

In this study, we obtained and characterized extreme subpopulations of addicted and nonaddicted male mice to identify the differential expression of miRNAs in the mPFC associated with vulnerability to food addiction. Using similar food addiction–like criteria, we have applied the YFAS 2.0 score to classify a cohort of patients of both sexes to assess the possible association of this behavioral disorder with circulating miRNAs. Finally, we functionally validated the involvement of selected miRNAs differentially expressed in addicted mice and in patients with food addiction in specific phenotypes of this addiction-like behavior.

## Results

### Selection of extreme subpopulations of addicted and nonaddicted mice.

**Figure 1:**
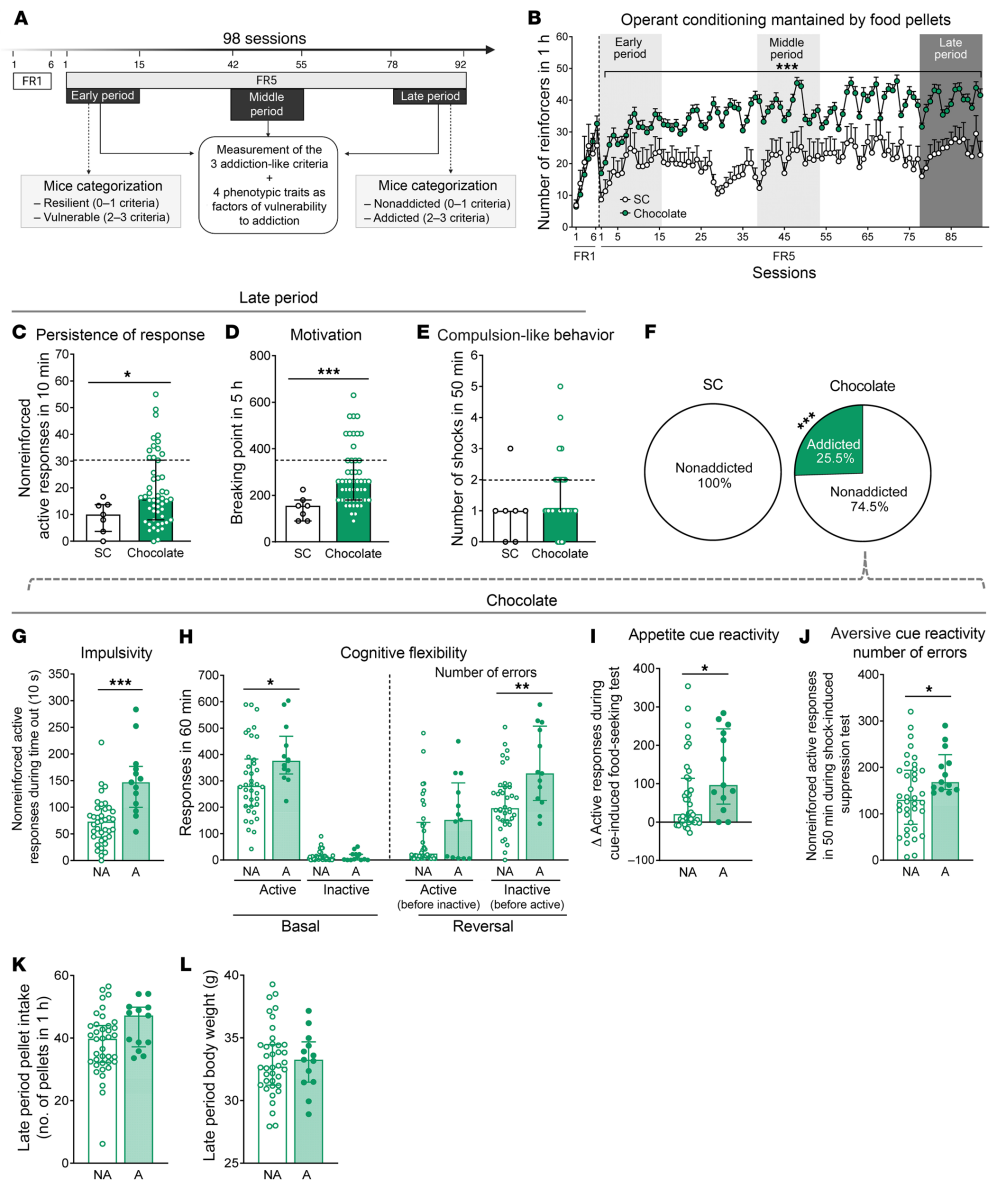
Extreme subpopulations of addicted and nonaddicted mice were selected among mice trained with chocolate-flavored pellets. (**A**) Timeline of the experimental sequence. (**B**) Operant conditioning maintained by chocolate-flavored or standard chow (SC) pellets. Mice trained with chocolate increased the number of reinforcers in 1 hour during FR5 daily sessions compared with mice trained with SC (data indicate the mean ± SEM; repeated-measures ANOVA; ***P < 0.001, pellet effect and pellets per session). (**C**–**E**) The 3 addiction-like criteria for the SC and chocolate-trained groups in the late period. (**C**) Persistence of response (Mann-Whitney *U*, *P < 0.05). (**D**) Motivation (Mann-Whitney *U*, ***P < 0.001). (**E**) Compulsion-like behavior. The dashed horizontal lines indicate the 75th percentile of the distribution of the chocolate-trained group. It was used as a threshold for considering a mouse positive for 1 criterion. (**F**) Percentage of addicted and nonaddicted mice trained with chocolate and SC pellets classified in the late period (χ^2^ test, ***P < 0.001). *n =* 51 mice trained with chocolate pellets; *n =* 7 mice trained with SC. (**G**–**J**) Tests for the 4 phenotypic traits in the late period for the chocolate-trained group, divided into addicted (A) and nonaddicted (NA) mice. (**G**) Impulsivity (Student’s *t* test, ***P < 0.001). (**H**) Cognitive flexibility (Mann Whitney *U*, *P < 0.05 and **P < 0.01. (**I**) Appetitive cue reactivity. Increased active response after presentation of the cue light (Mann-Whitney *U*, *P < 0.05). (**J**) Aversive cue reactivity. The number of nonreinforced active responses after the shock test with the same discriminative stimulus (grid floor) for the shock test. Pressing the active lever had no consequences: no shock, no pellets, and no cue light (Student’s *t* test, *P < 0.05). (**K**) Pellet intake and (**L**) body weight for mice classified as addicted or nonaddicted among those trained with chocolate pellets. *n =* 38 addicted mice; *n =* 13 nonaddicted mice trained with chocolate pellets. Data are presented as individual values with the IQR. Statistical details are included in Supplemental Table 10.

To study the miRNA signatures underpinning the susceptibility to developing food addiction–like behavior toward palatable food, C57Bl/6J mice (*n =* 58) underwent a long operant food addiction protocol (98 days) to obtain standard or palatable pellets (Figure 1A and Supplemental Figure 1, A and B; supplemental material available online with this article; https://doi.org/10.1172/JCI156281DS1). We have already used a similar operant protocol to identify resilient mice and mice that are vulnerable to developing this behavior in previous studies (8, 22). The different sample sizes of mice trained with standard (*n =* 7) or palatable (*n =* 51) pellets were based on the power analysis calculation using Power and Precision Software, vesion 4, which took into account the results of our previous studies in order to obtain a significant percentage of addicted mice. The male sex was chosen, considering the previous literature on food (8, 10, 23) and drug (24, 25) addiction models. In spite of all these studies previously performed with male rodents, further studies will be necessary to validate these models in female mice and rats. During fixed ratio 1 (FR1), mice trained with standard or palatable pellets had the same intake. However, as expected, the palatable pellet–trained group showed a higher number of responses than did the standard pellet–trained group in the FR5 period (Figure 1B). Indeed, we reported similar differences in operant responses for standard and palatable pellets in our previous studies showing the high reinforcement value of this palatable food (10). The 3 hallmark criteria of food addiction — persistence of response, motivation, and compulsion-like behavior — were evaluated during the early (days 1–15), middle (days 42–55), and late (days 78–92) training periods. In the early and middle periods, significant differences between mice trained with palatable pellets and those trained with standard pellets were evident only for the persistence of response and motivation criteria, respectively (Supplemental Figure 1, C–H). In the late period, chocolate-trained mice showed a higher persistence of response, motivation, and compulsion-like behavior compared with mice trained with standard pellets (Figure 1, C–E), revealing the requirement of a long training period to fully develop all the manifestations of food addiction in this mouse model. In this late period, mice were categorized according to the 3 addiction-like criteria previously used in our food addiction mouse model (8, 10) based on the DSM-5 for substance use disorders and the YFAS 2.0 food addiction diagnosis: persistence of response, motivation, and compulsion-like behavior (26, 27). Mice that achieved 2 or 3 addiction-like criteria were considered addicted, and mice that achieved 0 or 1 criterion were considered nonaddicted, in agreement with our previous studies (8, 10) and the requirement of 55% achievement of total criteria for severe diagnosis of the substance use disorder in the DSM-5. We found that 25.5% of mice trained with chocolate pellets achieved 2–3 criteria (addicted mice) compared with 0% of standard pellet–trained mice (Figure 1F), confirming that only palatable food, not standard chow, was able to trigger the addiction behavior (10). Consequently, further analyses of addictive behavioral differences and evaluation of miRNA signatures of addiction were performed only in mice trained with chocolate-flavored pellets, without any further analysis of mice trained with standard pellets. In agreement, significant positive correlations between the number of criteria achieved and the results obtained for each specific criterion confirmed that addicted mice had high values for all criteria (Supplemental Figure 1, I–K). We also evaluated in our operant paradigm 4 well-recognized phenotypic traits related to addiction (22, 28, 29). The first trait is impulsivity, which involves the inability to change the course of an action once it is initiated (30). The second trait is cognitive inflexibility, which indicates the inability to shift responses to stimuli that have previously predicted the availability of reward (31). The third trait is appetitive cue reactivity, which refers to the strength shown in the association of the appetitive stimuli with the cue (32). And the fourth trait is aversive cue reactivity, which indicates the value of the aversive cue in controlling the behavior (33). We found higher impulsivity, cognitive flexibility impairment, appetitive cue reactivity, and lower aversive cue reactivity in the addicted animals compared with the resilient mice (Figure 1, G–J, and Supplemental Figure 2, A–J). The pellet intake number and body weight were similar between addicted and nonaddicted mice (Figure 1, K and L). We also found significant positive correlations between the number of criteria achieved and each phenotypic trait (Supplemental Figure 2, C–F) underlying the relevance of these behavioral traits for the food addiction phenotype. Furthermore, the levels of impulsivity, cognitive flexibility, and appetitive cue reactivity increased over time in addicted mice, whereas they remained stable in nonaddicted mice (Supplemental Figure 2, G–I). In contrast, aversive cue reactivity was stable across time in both groups (Supplemental Figure 2J). Therefore, we have identified in our operant behavioral model a particular subgroup of mice vulnerable to developing food addiction after a long operant training period and characterized the main behavioral traits of these mice.

### Principal component analysis revealed differential patterns of behavioral factor loadings in food addiction–like behavior in mice.

**Figure 2:**
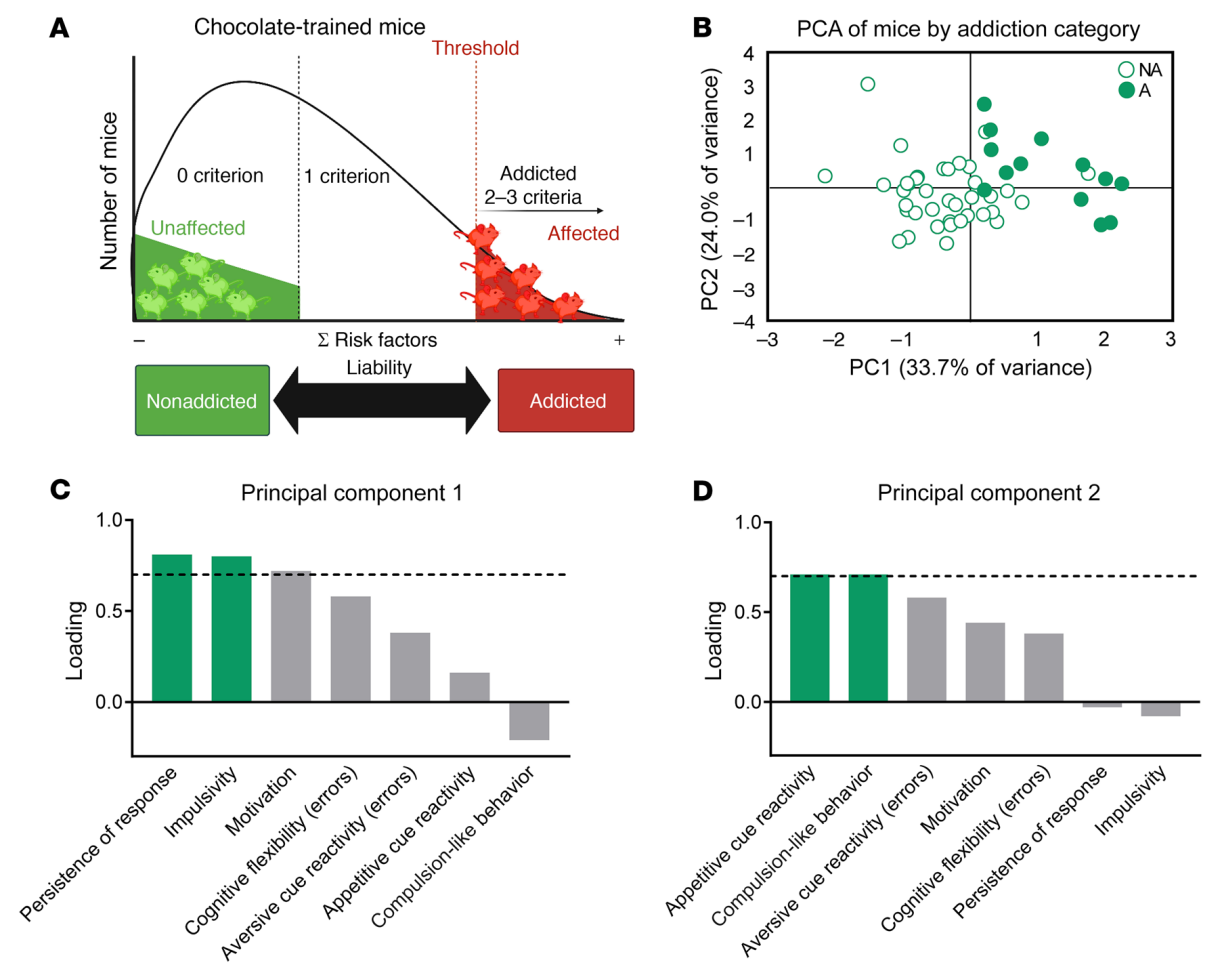
PCA of the 3 addiction criteria and the 4 phenotypic traits. (**A**) Inverted U-shaped curve showing that operant training with chocolate-flavored pellets allowed for the differentiation of extreme subpopulations of addicted and nonaddicted mice. (**B**) Individual mice clustered according to addiction or nonaddiction in the space yielded by 2 components of the PCA, which accounted for the maximum data variance, with factor loadings of PC1 (33.7%) and PC2 (24%). (**C** and **D**) Order of factor loading of the different variables in PC1 and PC2. The dashed horizontal line marks loadings greater than 0.7, mainly contributing to the component. With respect to the addiction criteria, a dissociation between persistence and motivation for 1 side and compulsion-like behavior for the other was observed. Impulsivity and cognitive flexibility weighted more in PC1, and both cue reactivities weighted more in the PC2.

We evaluated the links between the different addiction-like behavioral criteria and the phenotypic traits defined in our behavioral paradigm in order to obtain objective criteria for the selection of mice either vulnerable or resistant to developing food addiction (Figure 2A). For this purpose, we performed a principal component analysis (PCA), which showed that the percentage of variance explained by the 2 principal components (PCs) was 33.7% (PC1) and 24.0% (PC2) (Figure 2B). The main addiction criteria loading in PC1 were persistence and motivation, whereas compulsion-like behavior was the predominant criterion in PC2 (Figure 2, C and D). Among the 4 phenotypic traits, impulsivity and cognitive flexibility had the highest loading in PC1, whereas aversive and appetitive cue reactivity comprised the main loading in PC2. PCA allowed the classification of 2 extreme phenotypes of mice vulnerable (*n =* 6) or resistant (*n =* 6) to developing food addiction–like behavior based on these criteria and phenotypic traits (Figure 2, A and B). These extreme subgroups of mice trained with chocolate pellets were selected to identify brain miRNA signatures of vulnerability to food addiction (Supplemental Figure 1A). Significant differences in all behavioral addiction hallmarks (Supplemental Figure 1, L–N) and phenotypic traits (Supplemental Figure 1, Q–T) were confirmed between these extreme sub-groups of addicted and nonaddicted mice. In contrast, we found no differences between groups with regard to pellet intake, indicating that the possible differential epigenetic changes were due to the addiction-like phenotype and not to distinct food intake or body weight (Supplemental Figure 1, O and P).

### miRNA signatures of vulnerability to addiction in mice.

**Figure 3:**
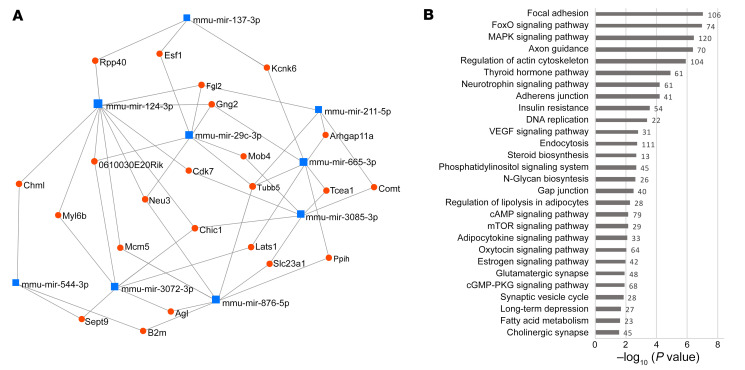
miRNAs differentially expressed in the mPFC of addicted mice. (**A**) Network from miRNet analysis based on the 9 downregulated miRNAs and their target genes, filtered to retain more nodes with more connections. (**B**) Selection of enriched KEGG pathways identified in the target genes of the downregulated miRNAs.

We performed small RNA-Seq of the mPFC, an area critically engaged in loss of eating control (34), to characterize miRNA expression signatures for food addiction vulnerability. We compared miRNA profiles of extreme subpopulations of resilient (*n =* 6) and vulnerable (*n =* 6) mice in a discovery sample and replicated the results in independent cohorts of resilient (*n =* 6) and vulnerable (*n =* 6) mice from a replica sample (Supplemental Figure 1A). For this purpose, we used a quantitative gradual addiction scale to order mice according to their degree of food addiction severity in the inverted U-shaped curve of the normal distribution (Supplemental Figure 1A). The discovery sample consisted of animals with the 6 most extreme values of this inverted U-shaped curve, and the 6 animals with the extreme values in the curve formed the replica sample. A comparison of the extreme resilient and vulnerable cohorts identified 11 miRNAs with significant differential expression in the mPFC both in the discovery and replica samples (Tables 1 and 2 and Supplemental Tables 1 and 2): 9 were underexpressed (mmu-miR-29c-3p, -124-3p, -137-3p, -211-5p, -544-3p, -665-3p, -876-5p, -3072-3p and -3085-3p), and 2 were overexpressed (mmu-miR-100-5p and -192-5p) in the addicted group compared with the resilient group (Table 1 and Supplemental Tables 1 and 2). We observed a significant overlap between discovery and replica only for the downregulated miRNAs, but not for the upregulated ones that were then excluded for potential functional validation studies (Table 2). Network analysis revealed that downregulated miRNAs and their target genes were highly interconnected (Figure 3A).

### Target genes regulated by the miRNAs altered in addicted mice.

Many of those miRNAs coregulate target genes involved in several pathways relevant to cognitive function and addiction, including long-term depression, glutamatergic synapse, cholinergic synapse, mTOR, cAMP, MAPK, oxytocin, and neurotrophin signaling pathways. These target genes are also involved in several pathways related to morphological changes in the nervous system, such as changes in axon guidance, focal adhesion, actin cytoskeleton, adherens junctions, and gap junctions, as well as pathways related to metabolism, including insulin resistance, lipolysis, adipocytokine, and the thyroid hormone pathway (Figure 3B, Supplemental Figure 3, A–F, and Supplemental Table 4).

We then explored whether the expression of the targets of these miRNAs in the mPFC were altered. For this purpose, we performed RNA-Seq of the samples from the same addicted and nonaddicted mice used to analyze the miRNA profile. We observed an enrichment of altered target genes for 9 of 11 miRNAs, in either the discovery or replica samples. Interestingly, these enrichments were revealed for the target genes of all the miRNAs underexpressed in the addicted group compared with the resilient mice: mmu-miR-29c-3p, -124-3p, -137-3p, -211-5p, -544-3p, -665-3p, -876-5p, -3072-3p and -3085-3p (Table 1). Thus, our results indicate that these particular miRNAs that were found to be differentially expressed affected the expression of their regulated transcripts in the mPFC of these addicted mice, thereby affecting the above-mentioned pathways potentially relevant for food addiction and related processes.

### miRNA signatures in humans.

**Figure 4:**
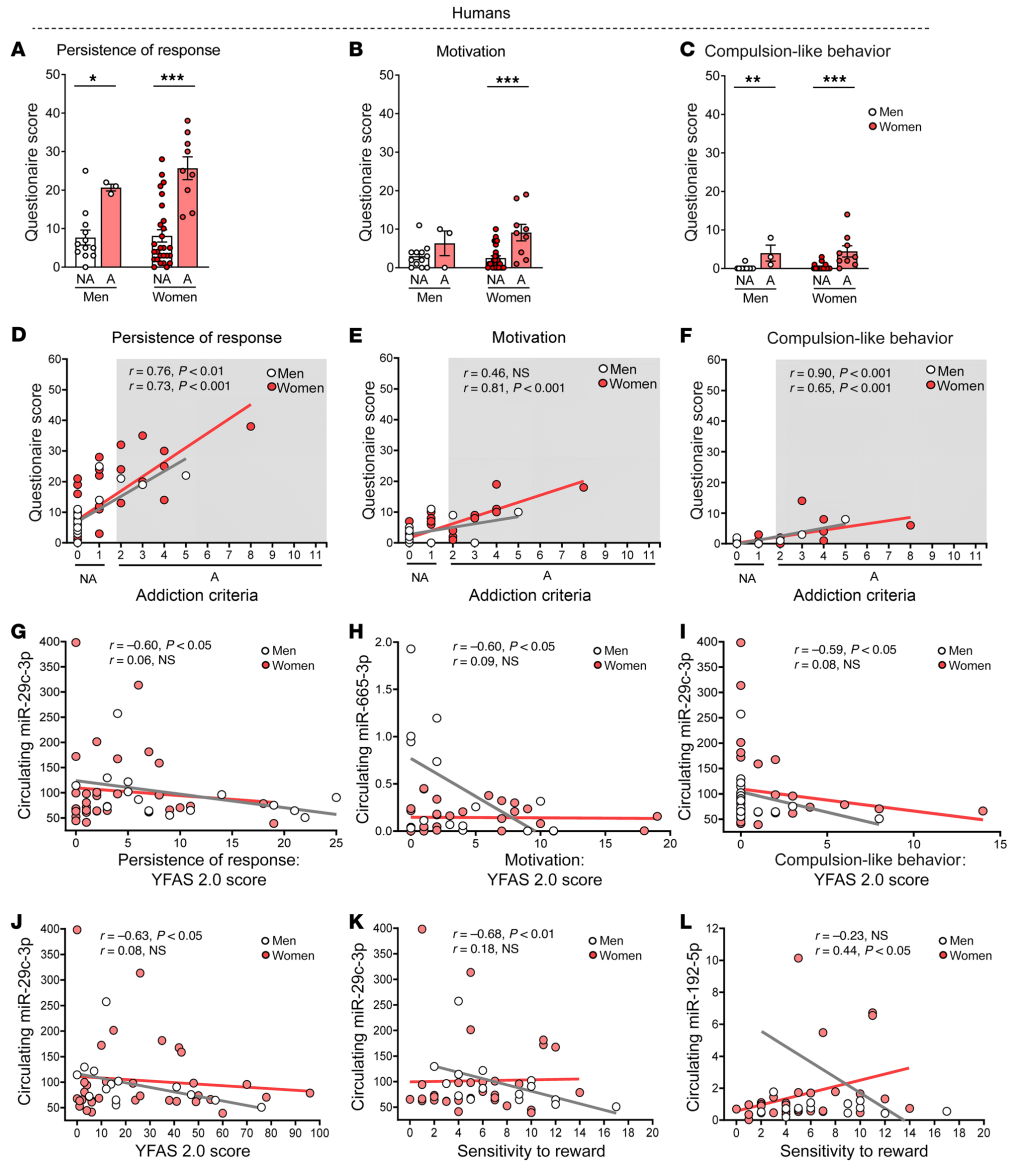
Behavioral results for the 3 hallmarks of addiction in a human cohort comparing nonaddicted and addicted individuals. (**A**) Persistence of response, (**B**) motivation, and (**C**) compulsion-like behavior (median with the IQR). *P < 0.05,**P < 0.01, and ***P < 0.001, by Mann-Whitney *U* test. (**D**–**F**) Pearson’s correlations between addiction-like criteria achieved and the questionnaire score obtained for (**D**) persistence of response, (**E**) motivation, and (**F**) compulsion-like behavior comparing nonaddicted and addicted participants. *n =* 51 participants total (*n =* 39 nonaddicted; *n =* 12 addicted). (**G**) Scatter correlation plot for the association between the expression of circulating hsa-miR-29c-3p and the persistence of response YFAS 2.0 score. (**H**) Scatter correlation plot for the association between the expression of circulating hsa-miR-665-3p and the motivation to response YFAS 2.0 score. (**I**) Scatter correlation plot for the association between the expression of circulating hsa-miR-29c-3p and the compulsion-like behavior YFAS 2.0 score. (**J**) Scatter correlation plot for the association between the expression of circulating hsa-miR-29c-3p and the YFAS 2.0 score. (**K**) Scatter correlation plot for the association between the expression of circulating hsa-miR-29c-3p and the sensitivity to reward score. (**L**) Scatter correlation plot for the association between the expression of circulating hsa-miR-192-5p and the sensitivity to reward score. Statistical details are included in Supplemental Table 11.

The YFAS 2.0 score was used for a cohort of patients (*n =* 51) to identify possible signatures of circulating miRNAs associated with food addiction. First, we evaluated whether the 3 addiction-like criteria measured in our food addiction mouse model accurately recapitulated the principal features of food addiction in our human cohort selected (Table 3) using the 35-item self-report YFAS 2.0. For this purpose, we analyzed the human data extracted from the YFAS 2.0 questionnaire, taking into account that several YFAS 2.0 questions could be grouped under these 3 addiction-like criteria used in mice. As expected, the sum of the YFAS 2.0 questions for the criteria of persistence of response, motivation, and compulsion-like behavior was much higher in participants diagnosed with food addiction than in nonaddicted individuals for both sexes (Figure 4, A–C). Notably, the severity of the food addiction diagnosis in humans (2–3 criteria: mild, 4–5 criteria: moderate, and 6–11: severe) positively correlated with the questionnaire score in the 3 addiction criteria of persistence of response, motivation, and compulsion-like behavior in women, whereas in men, only the persistence of response and compulsion-like behavior were positively correlated, indicating that a greater severity of disease means higher scores for the 3 hallmarks of addiction (Figure 4, D–F).

Interestingly, we found similitudes in the association between these behavioral hallmarks of addiction and circulating miRNA signatures in humans and those previously revealed in our animal studies. In agreement, the circulating levels of hsa-miR-29c-3p were negatively correlated with persistence of response and compulsion-like behavior in men, and hsa-miR-665-3p levels were also negatively correlated with motivation in this sex (Figure 4, G–I). Indeed, the circulating levels of hsa-miR-29c-3p were negatively associated with both the YFAS 2.0 (Figure 4J) and the sensitivity to reward (Figure 4K) scores in men, in exact agreement with the findings in our mouse model of food addiction. Circulating levels of hsa-miR-29c-3p and hsa-miR-665-3p did not correlate with these reward-related behavioral responses in women. Conversely, the circulating levels of hsa-miR-192-5p were positively associated with the sensitivity to reward score for women, but not for men (Figure 4L). Circulating levels of hsa-miR-29c-3p and hsa-miR-665-3p were also negatively correlated with BMI in individuals with high YFAS 2.0 scores (data not shown). We next assessed these miRNAs in a large GWAS of BMI that included more than 700,000 individuals and found that the MIR-665 gene was significantly associated with BMI (*P =* 0.02). Interestingly, target genes for hsa-miR-29c-3p and hsa-665-3p were significantly enriched among those genes that were significantly associated with BMI in this large GWAS cohort (Supplemental Table 5), further suggesting that both miRNAs are relevant candidates for involvement in food addiction.

### Functional validation of candidate miRNAs.

**Figure 5:**
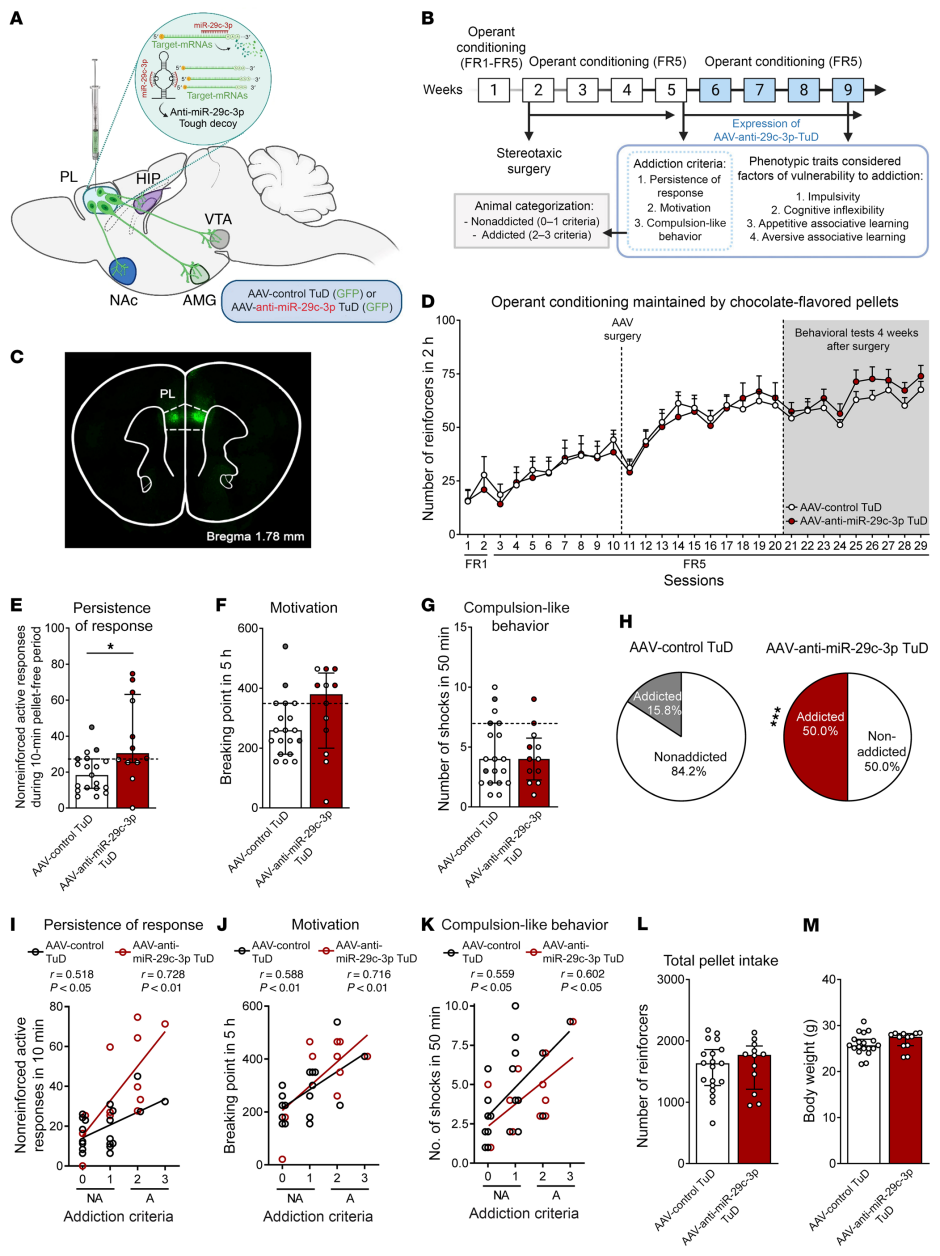
Functional validation of candidate mmu-miR-29c-3p inhibition. (**A**) Scheme of viral strategy for inhibiting mmu-miR-29c-3p in PL neurons. Schematic representation of miRNA-mRNA interaction in basal conditions and of the miRNA inhibitor TuD mechanism. (**B**) Experimental design. (**C**) Representative fluorescence images showing virus-dependent GFP protein expression at the PL injection site. (**D**) Number of reinforcers during operant training sessions maintained by chocolate-flavored pellets, comparing AAV control TuD–treated mice and AAV–anti–mmu-miR-29c-3p TuD–treated mice. (**E**–**G**) Behavioral tests for the 3 addiction-like criteria showed increased persistence in mice with mmu-miR-29c-3p inhibition (individual values are shown with the median and IQR; Student’s *t* test, *P < 0.05). Addicted mice are indicated by filled circles. (**H**) An increase in the percentage of miR-29c-3p–underexpressing mice classified as food addicted was observed (χ^2^ test, ***P < 0.001). (**I**–**K**) Pearson’s correlations between individual addiction-like criteria and (**I**) nonreinforced active responses in a 10-minute period, (**J**) the breaking point in 5 hours, (**K**) the number of shocks in a 50-minute period, (**L**) pellet intake, and (**M**) body weight (*n =* 19, AAV control TuD mice; *n =* 12, AAV–anti–mmu-miR-29c-3p-TuD mice). Statistical details are provided in Supplemental Table 12.

Given the results from these mouse and human cohorts, we performed a functional validation of the most promising candidate miRNAs identified in the mPFC of addicted mice and in plasma samples from our human cohort. The selection of the miRNA candidates was based on the expression levels and enrichment of target genes that showed differential expression (Figure 3A and Table 1) as well as on previous literature about the functional role of these miRNAs. We first selected the miRNA mmu-miR-29c-3p, which was found to be strikingly similar in both our animal and human cohorts and previously reported to be an epigenetic marker related to methamphetamine addiction (35). Target genes of this miRNA are enriched in several pathways relevant to the addiction process (dopaminergic synapse, MAPK, and neurotrophin signaling pathways), neuronal morphological changes (axon growth, axon guidance, cellular adhesion, and focal adhesion), and pathways related to digestion and metabolism (carbohydrate digestion and absorption, insulin signaling pathway, and insulin resistance) (Supplemental Table 6). We aimed to mimic the underexpression of mmu-miR-29c-3p observed in the mPFC of addicted mice, which is in agreement with the negative correlation with the YFAS 2.0 score found in our human cohort. Thus, we used a tough decoy (TuD) inhibitor with an adeno-associated virus (AAV) anti–mmu-miR-29c-3p TuD-GFP, previously validated (35), which was stereotaxically microinjected into the PL mPFC to selectively inhibit mmu-miR-29c-3p function (Figure 5, A and B). We have previously demonstrated the crucial role of this subregion of the mPFC in the loss of control of food intake (8). After bilateral microinjection of TuD, the mice underwent an operant conditioning schedule, as previously described (8, 22), to evaluate, following short operant training, the possible early development of food addiction due to these epigenetics manipulations (Figure 5A). All the mice included in our behavioral analysis were correctly microinjected in this subregion of the mPFC, as revealed by GFP detection (Figure 5C). Inhibition of mmu-miR-29c-3p in the PL area significantly increased the persistence of response and enhanced motivation during a short operant training period of 29 sessions, and the percentage of mice that achieved addiction-like criteria with palatable pellets was 50.0% compared with 15.8% of mice injected with the control TuD (Figure 5, E–H). In contrast, no significant differences in palatable food reinforcement during FR1 and FR5, compulsion-like behavior, or other behavioral phenotype traits were observed (Figure 5D and Supplemental Figure 5, A–D). We noted a positive correlation between the number of criteria achieved and the values obtained for each criterion (Figure 5, I–K). With mmu-miR-29c-3p underexpression, we observed no differences in other variables, such as body weight or food intake (Figure 5, L and M). These results demonstrate a crucial role of miR-29c in the mPFC in the development of 2 particular hallmarks of food addiction: persistence of response and motivation.

**Figure 6:**
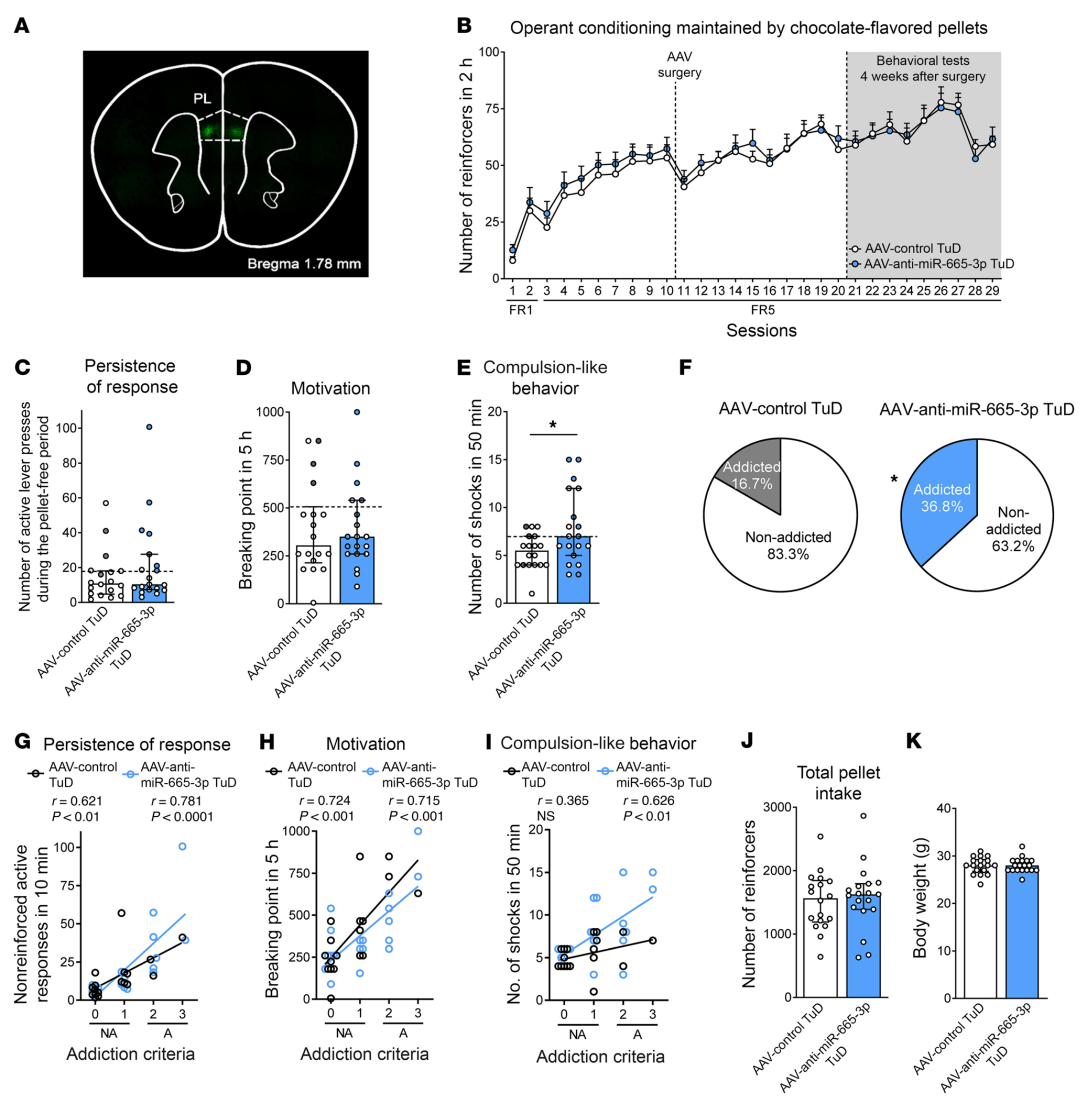
Functional validation of candidate mmu-miR-665-3p inhibition. (**A**) Representative fluorescence images showing virus-dependent GFP protein expression at the PL injection site. (**B**) Number of reinforcers during operant training sessions maintained by chocolate-flavored pellets comparing AAV control TuD mice and AAV–anti–mmu-miR-665-3p TuD mice. (**C**–**E**) Behavioral tests for the 3 addiction-like criteria showed increased compulsion-like behavior in mice with mmu-miR-665-3p inhibition (individual values with the median and IQR are shown; Student’s *t* test, *P < 0.05). Addicted mice are indicated by filled circles. (**F**) Increased percentage of mice with mmu-miR-665-3p inhibition classified as food-addicted animals (χ^2^ test, *P < 0.05). (**G**–**I**) Pearson’s correlations between individual addiction-like criteria and (**G**) nonreinforced active responses in a 10-minute period, (**H**) the breaking point in 5 hours, (**I**) the number of shocks in a 50-minute period, (**J**) pellet intake, and (**K**) body weight (*n =* 18, AAV control TuD mice; *n =* 19, AAV–anti–mmu-miR-665-3p TuD mice). Statistical details are provided in Supplemental Table 13.

Considering the preferential involvement of mmu-miRNA29c-3p alone in these specific hallmarks of addiction, we extended our validation studies to 2 additional miRNAs, mmu-miR-665-3p and mmu-miR-137-3p. The mmu-miR-665-3p miRNA was previously associated with regulation of the expression of cannabinoid receptors in patients with severe heart failure and with changes in microbiota composition (36). The mmu-miR-137-3p miRNA was selected for its involvement in psychiatric disorders, including schizophrenia and impaired sociability (37). We used the same TuD strategy to selectively inhibit mmu-miR-655-3p and mmu-miR-137-3p in the PL mPFC for evaluation of its involvement in the development of food addiction (Figure 5, A and B). We found that inhibition of mmu-miR-665-3p in the PL mPFC enhanced compulsion-like behavior after short operant training, without modifying the other hallmarks of addiction (Figure 6, C–E), the number of reinforcers maintained by chocolate-flavored pellets during FR1 and FR5, or other behavioral phenotype traits (Figure 6B and Supplemental Figure 6, A–D). Accordingly, a subset of mice (36.8%) with inhibition of mmu-miR-665-3p in this brain area reached addiction-like criteria in this short training period, a percentage markedly higher than that for control mice (16.7%) (Figure 6F). All of the mice included in the study were correctly microinjected in this mPFC subregion, as revealed by GFP detection (Figure 6A). We found positive correlations between the number of criteria reached and the values obtained for each criterion (Figure 6, G–I), with no significant differences in body weight or food intake (Figure 6, J and K).

**Figure 7:**
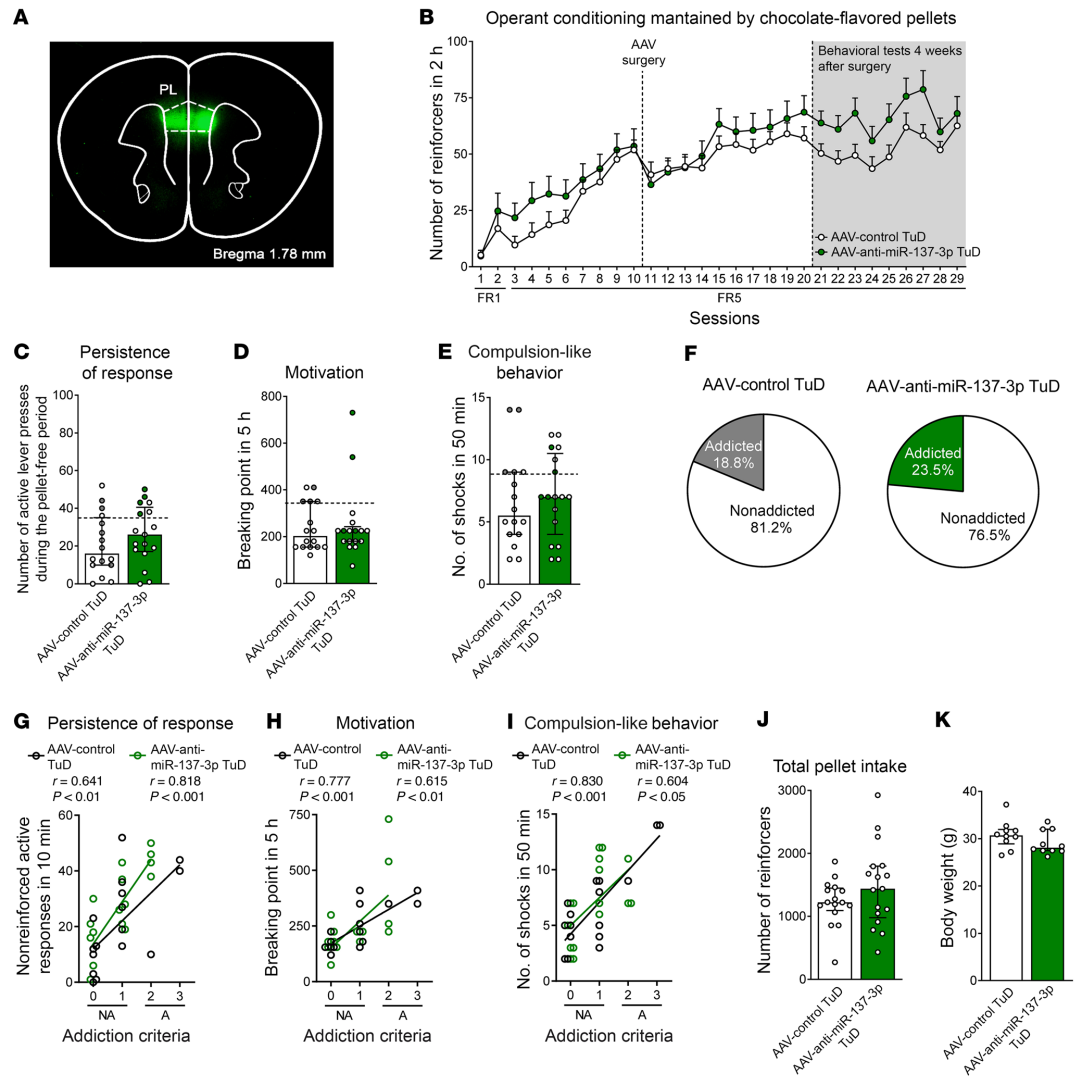
Functional validation of candidate mmu-miR-137-3p inhibition. (**A**) Representative fluorescence images showing virus-dependent GFP protein expression at the PL injection site. (**B**) Number of reinforcers during operant training sessions maintained by chocolate-flavored pellets comparing AAV control TuD mice and AAV–anti–mmu-miR-137 TuD mice. (**C**–**E**) Behavioral tests for the 3 addiction-like criteria did not show an increase in the addiction phenotype in mice with mmu-miR-137 inhibition (individual values with the median and IQR are shown). Addicted mice are indicated by filled circles. (**F**) The percentage of mmu-miR-137–underexpressing mice classified as food-addicted animals did not differ from that of the control group (χ^2^ test, NS). (**G**–**I**) Pearson’s correlations between individual addiction-like criteria and (**G**) nonreinforced active responses in a 10-minute period, (**H**) the breaking point in 5 hours, (**I**) the number of shocks in a 50-minute period, (**J**) pellet intake, and (**K**) body weight (*n =* 16, AAV control TuD mice; *n =* 17, AAV–anti–mmu-miR-137 TuD mice). Statistical details are provided in Supplemental Table 14.

Finally, we also performed a functional validation study of mmu-miR-137-3p using TuD in the PL mPFC (Figure 5, A and B). All of the mice included in the study were correctly microinjected in this mPFC subregion, as confirmed by GFP expression (Figure 7A). Inhibition of this miRNA in mice did not yield significant differences when compared with the control group for any of the 3 addiction-like criteria, reinforcers for palatable food during FR1 and FR5, or behavioral phenotype traits, whereas positive correlations between the number of criteria and the values obtained for each criterion were found, as expected (Figure 7, B–I, and Supplemental Figure 7). No significant differences in body weight or food intake were revealed after mmu-miR-137-3p inhibition (Figure 7, J and K).

We validate that our TuD strategy efficiently inhibited the function of the target miRNAs by assessing the expression of related target genes in the PL mPFC. We found that expression of the mmu-miR-665-3p target genes *Ncam1* and *Rbfox1* was significantly upregulated in AAV anti–mmu-miR-665-3p TuD and that expression of these genes was also higher in addicted TuD mice than in nonaddicted mice (Supplemental Figure 4, A and B). Therefore, the inhibition of mmu-miR-29c-3p and mmu-miR-665-3p expression in the PL mPFC enhanced the vulnerability to developing food addiction, whereas mmu-miR-137-3p inhibition in this brain area did not modify food addiction–like behavior.

### Target genes regulated by miR-29c-3p, miR-665-3p, and miR-137-3p.

**Figure 8:**
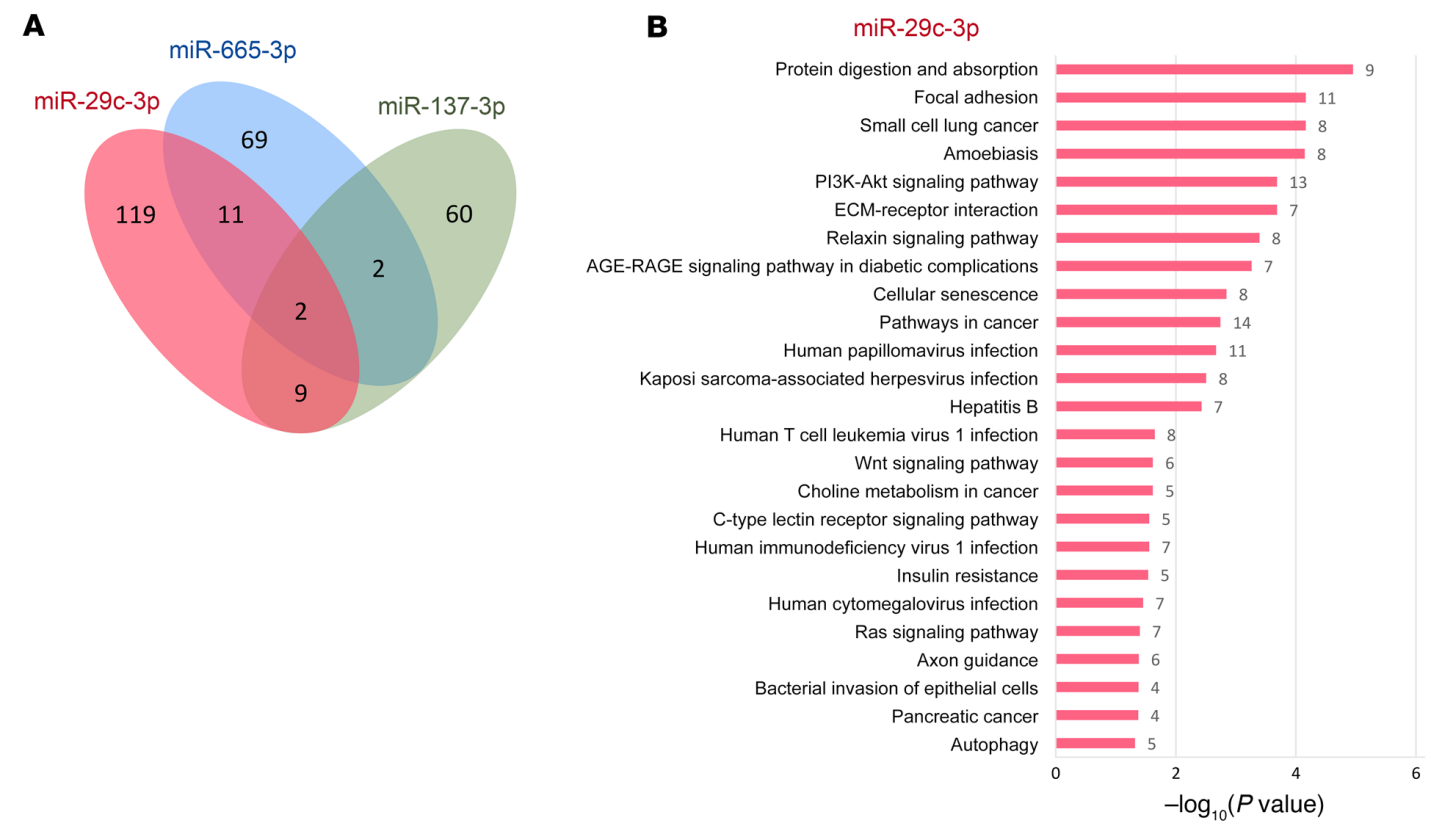
Differentially expressed targets of miR-29c-3p, miR-665-3p, and miR-137-3p. (**A**) Venn diagram of the genes found to be differentially expressed that are targets of these 3 miRNAs. Genes were considered differentially expressed only when the target genes were enriched in the RNA-Seq data in the discovery sample (statistically significant for miR-29c-3p and miR-665-3p) or in the replication sample (miR-29c-3p, miR-665-3p, and miR-137-3p; Table 1). (**B**) Enriched KEGG pathways identified in the target genes of miR-29c-3p.

To further understand the regulatory mechanisms of these 3 miRNAs, we selectively inspected their differentially expressed target genes using the RNA-Seq data from the discovery and replica samples (Supplemental Table 3). We found a small overlap between differentially expressed targets of these 3 miRNAs, suggesting that most were regulated specifically by each corresponding miRNA (Figure 8A). This is in line with the findings in our functional validation using the TuD approach, in which mmu-miR-29c-3p and mmu-miR-665-3p were found to affect different behavioral hallmarks of addiction. Interestingly, we found that the differentially expressed targets of mmu-miR-29c-3p are involved in Kyoto Encyclopedia of Genes and Genomes (KEGG) pathways such as those for focal adhesion, insulin resistance, axon guidance, PI3/AKT and relaxin signaling (Figure 8B and Supplemental Table 7), as well as in Reactome pathways for neurite outgrowth signaling and extracellular matrix organization (Supplemental Table 8). In the case of mmu-miR-665-3p, differentially expressed target genes were enriched in the metabolism of lipids, according to the Reactome pathways database (Supplemental Table 8). Among the enriched gene ontology (GO) biological processes in the differentially expressed targets (Supplemental Table 9), we found mRNA destabilization, extracellular matrix organization, and cell adhesion for mmu-miR-29c-3p; cellular lipid metabolic process for mmu-miR-665-3p; and adult walking behavior for mmu-miR-137-3p (Supplemental Table 9). Interestingly, the gene *Trp53inp2*, a target of mmu-miR-29c-3p and mmu-miR-665-3p, was differentially expressed in the discovery and replica samples, and its encoded transcript participates in axonal growth by interacting with the nerve growth factor (NGF) receptor TrkA. *Trp53inp2* is also involved in processes of morphological changes in the nervous system (38). Also, 2 differentially expressed genes, *Luzp1* and *Mtmr4*, were targets of all 3 miRNAs (mmu-miR-29c-3p, mmu-miR-665-3p, and mmu-miR-137). *Luzp1* encodes a protein involved in the actin cytoskeleton that participates in neuronal morphological changes. Remarkably, in a GWAS of eating behavior in pigs, *Mtmr4*, which is involved in dephosphorylation, was found to be highly associated with feeding behavior (39).

## Discussion

In the present study, we investigated the changes in miRNA expression promoted by food addiction in animals and humans and the involvement of these miRNAs in the mechanisms underlying the behavioral hallmarks of this disorder. For this purpose, we used similar food addiction–like criteria for male mice and humans of both sexes to select subpopulations with extreme addiction or no addiction. We evaluated miRNA expression in the mPFC in the mouse cohort and plasma circulating miRNA levels in the human cohort. Two subgroups of extreme phenotypes of mice vulnerable or resilient to developing food addiction were selected based on the basis of the 3 addiction-like criteria and the main related phenotypic traits. Our behavioral characterization took into account each specific endophenotype that integrates this complex behavioral disease. PCA of the mouse behavioral responses revealed a predominant load of 2 hallmarks of addiction, persistence of response and motivation, in a first component defining these extreme phenotypes, whereas the major weight in the second component corresponded to the third addiction criterion, compulsion-like behavior. Persistence of response is associated with difficulty in stopping reward seeking due to habit formation or disruption of extinction learning and has been reported to involve the mPFC (5, 40), whereas compulsion-like behavior has been directly related to the network activity of the mPFC glutamatergic projections to the NAc (8). Motivation is linked to reward processing, and the mPFC has also been associated with the decision-making process evaluated in our motivation paradigm (41).

Here, we showed that the extreme phenotype groups of mice had a particular miRNA signature in the mPFC, an area closely involved in addictive behavior (5, 8, 28, 40, 41). This signature was characterized by a predominant downregulation of various relevant miRNAs in addicted mice and altered expression of their specific target genes. Subsequent analysis of the target genes of these downregulated miRNAs showed enrichment in gene pathways related to cognitive processes, which are essential for the main behavioral components of addictive behavior, including persistence of response, motivation, compulsion-like behavior, craving, and relapse (28). The downregulated miRNAs also target genes involved in other pathways relevant to addiction, such as pathways for glutamatergic and cholinergic transmission, MAPK, and neurotrophin signaling, which play a crucial role in compulsion-like behavior, craving, relapse, and neuronal morphological changes (28). Also, the oxytocin signaling pathway regulated by these miRNAs is relevant in addiction for its role in the mesocorticolimbic dopaminergic pathway modulating reward-related processes (42). *Oxtr*, a key gene in the oxytocin signaling pathway that encodes for the oxytocin receptor, is a target of miR-29c-3p, which was differentially expressed in the discovery and replica samples. Several of the genes targeted by these miRNAs are also involved in pathways related to metabolism and obesity, including insulin resistance, lipolysis, adipocytokines, and thyroid hormone pathways. The loss of behavioral control that characterizes the hallmarks of addiction and the related behavioral phenotype traits are frequently seen in patients with obesity (12, 41), underlining the relevance of these common pathways regulated by the targeted genes. Interestingly, mmu-miR-29c-3p– and mmu-miR-665-3p–targeted genes are also involved in the majority of these pathways, highlighting the potential relevance of these particular miRNAs in neuronal functions that are essential for the development of food addiction.

Addiction-like criteria similar to those used in our animal studies were used to classify our cohort of patients according to the YFAS 2.0 in order to evaluate the correlation of these human behavioral alterations with circulating miRNA levels for a translational comparison of our animal and human findings. To ensure the translational value of the behavioral parameters evaluated, we first verified that the 3 addiction criteria measured in our mouse model recapitulated the principal features of food addiction in our human cohort. As expected, the participants diagnosed with food addiction had the highest scores for the YFAS 2.0 questions according to the criteria of persistence of response, motivation, and compulsion-like behavior. Taking into account the previously reported sexual dimorphism in all kinds of reward-related behaviors (43, 44), including the transcriptomic profiles associated with addiction (45) and stress vulnerability (44), we investigated this possible dimorphism in the behavioral responses evaluated in our human cohort. Addicted individuals of both sexes had similar highest and significantly correlated scores for persistence of response, motivation, and compulsion-like behavior, although the motivation results for men did not reach significance, probably due to the limited number of men that obtained a high YFAS 2.0 score in this food addiction criterion and were classified as addicted. Interestingly, we identified sharp similitudes in the miRNA signatures in our animal and human cohorts. The most striking correlations were found in men with peripheral circulating levels of hsa-miR-29c-3p. Interestingly, the circulating levels of this miRNA in men correlated with the YFAS 2.0 score for sensitivity to reward, persistence of response, compulsion-like behavior, and BMI in the same negative direction as its PFC expression was observed in our male mouse cohort, pointing to hsa-miR-29c-3p as a potential biomarker for this eating disorder. Hsa-miR-665-3p also correlated with motivation and BMI in our male human cohorts to the same degree and direction as this miRNA’s correlation with vulnerability to developing addiction in male mice. Women did not show any significant correlation of hsa-miR-29c-3p or hsa-miR-665-3p with the different behavioral responses evaluated. Indeed, only hsa-miR-192-5p was correlated with sensitivity to reward in women, but in the opposite direction of what was observed in men, underlining the existence of sexual dimorphism in these reward-related behavioral responses.

Given the close similarities between the animal and human results for miR-29c-3p with regard to correlations with food addiction scores, we first validated in mice the functional relevance of this miRNA in the mPFC. The PL subregion of the mPFC was chosen for this validation because of its crucial role in vulnerability to developing food addiction (8). We found that mmu-miR-29c-3p inhibition in this brain area dramatically enhanced the development of food addiction–like behavior in mice. Indeed, half of the mice exposed to this epigenetic modification reached the addiction-like criteria in a short training period compared with a low percentage of control mice that reached these criteria. Interestingly, this epigenetic change mainly affected 2 particular hallmarks of addiction: persistence of response and enhanced motivation of mice. Persistence of response and motivation were the hallmarks of addiction, with the main load in the first component identified by PCA defining this behavioral alteration. However, mmu-miR-29c-3p inhibition had no effect on the third addiction-like criterion, compulsion-like behavior. Taking into account that the inhibition of this first miRNA had a partial effect on this addictive behavior, we also selected 2 additional miRNAs, mmu-miR-665-3p and mmu-miR-137-3p, for functional validation, given their role in responses related to metabolic (24, 25) and psychiatric disorders (28) and the similar types of changes found in our mouse and human cohorts with regard to mmu-miR-665-3p. Inhibition of mmu-miR-665-3p in the PL mPFC also markedly enhanced the development of food addiction–like behavior. Interestingly, this epigenetic change mainly altered compulsion-like behavior, a criterion unaffected by mmu-miRNA29c-3p inhibition that represents the main addiction hallmark in the second PCA component of this behavioral disorder. In contrast, inhibition of mmu-miRNA-137-3p in this brain area had no impact on the development of food addiction–like behavior. In agreement with the results of this behavioral validation, enrichment of the differentially expressed gene targets of mmu-miR-29c-3p and mmu-miR-665-3p was found in the discovery and replication samples, but in the case of mmu-miR-137-3p, enrichment was only observed in the replication sample, which could partly explain the results obtained. It is remarkable that miR-29c-3p and miR-665-3p contribute to food addiction by differently and selectively affecting each 1 of the 2 main behavioral components that define this disorder, possibly mediating together an important interplay between genes and environment relevant to the development of food addiction. Furthermore, in humans, BMI is associated with genetic risk variants present in the MIR-665 gene and also in the target genes of both hsa-miR-665-3p and hsa-miR-29c-3p, providing additional support for the important role of these miRNAs in the vulnerability to developing food addiction described in our study.

Finally, we also evaluated the expression of mmu-miR-29c-3p, mmu-miR-665-3p, and mmu-miR-137-3p target genes in our mouse cohort to gain insights into the possible regulatory mechanisms involved in the behavioral responses modified by downregulating these miRNAs. In agreement with the different behavioral responses obtained after the downregulation of these miRNAs, we found a small overlap in their differentially expressed target genes in our cohort of mice. The differentially expressed target genes of mmu-miR-29c-3p and mmu-miR-665-3p are mainly involved in several pathways related to morphological changes in the nervous system, such as pathways for axon growth and guidance as well as those for cellular and focal adhesion. These changes suggest the possible involvement of morphological and/or neuronal connectivity changes in the behavioral effects on food addiction promoted by these miRNAs, in agreement with the morphological (46, 47) and functional connectivity changes previously reported in drug addiction and obesity (12). Our validation studies therefore allowed us to identify the specific role of miR-29c-3p and miR-665-3p in each main behavioral component of food addiction in a cortical area crucial for the development of this disorder, the PL mPFC (8), and suggest a possible involvement of morphological and/or neuronal connectivity changes in these mechanisms.

The identification of a differential miRNA signature in mice and humans vulnerable to developing food addiction and of the functional role of miR-29c-3p and miR-665-3p in specific behavioral components of this disorder represents what we believe to be a relevant advance in the understanding of the epigenetic mechanisms underlying food addiction. We believe the similitudes between our animal and human results provide important translational value that supports the applicability of our findings. This understanding of the role of miRNAs in the development of food addiction may lead to new approaches to identifying possible biomarkers for the early diagnosis of this disorder. Our findings may also be helpful for the future development of innovative therapies to treat food addiction and related eating disorders using the strategies now available to modify miRNA activity and expression.

## Methods

Detailed methods are provided in the Supplemental Methods.

The 3 addiction-like criteria considered in the food addiction mouse model were evaluated using 3 specific behavioral tests. These criteria summarized the hallmarks of addiction based on the DSM-5 for substance use disorders and the food addiction diagnosis through the YFAS 2.0 (26, 27). (a) Persistence of response is the criterion that measures persistent desire for a reward. It is measured during 3 consecutive sessions of FR5, before the progressive ratio test of motivation (Supplemental Figure 1B), and considers the nonreinforced active responses during the 10-minute pellet-free period of an FR5 session, when the box is illuminated and signaling the unavailability of pellet delivery, as persistence of food-seeking behavior or unsuccessful efforts to cut down. (b) Motivation is measured with the progressive ratio (PR) schedule of reinforcement to evaluate the effort to obtain the chocolate-flavored pellets (8). The response required to earn 1 single pellet escalated according to the following series: 1, 5, 12, 21, 33, 51, 75, 90, 120, 155, 180, 225, 260, 300, 350, 410, 465, 540, 630, 730, 850, 1000, 1200, 1500, 1800, 2100, 2400, 2700, 3000, 3400, 3800, 4200, 4600, 5000, and 5500. The maximum number of responses that the animal made to obtain 1 pellet was the last event completed, referred to as the breaking point. The maximum duration of the PR session was 5 hours or until the mouse did not respond on any lever within 1 hour. (c) Compulsion-like behavior was measured as the total number of shocks in the shock test session (50 min). Each pellet delivery was associated with an electric footshock as a punishment in this test. In this shock session, the mice were under an FR5 schedule of reinforcement for 50 minutes with 2 scheduled changes: at the fourth active lever response, the mice received only an electric footshock (0.18 mA, 2 s) without pellet delivery, and at the fifth active lever response, the mice received another electric footshock with a chocolate-flavored pellet paired with the cue light. The schedule was reinitiated after 10 seconds of pellet delivery (time-out period) and after the fourth response if the mice did not perform the fifth response within 1 minute.

The addiction-like criteria were attributed after the performance of the mice in these 3 behavioral tests. Then, the mice were categorized as addicted or nonaddicted depending on the number of positive criteria they had achieved. A mouse was considered positive for an addiction-like criterion when the score of the specific behavioral test was above the 75th percentile of the normal distribution of the palatable food–trained mice in the control group. Mice that achieved 2 or 3 addiction-like criteria were considered addicted, and mice that achieved 0 or 1 addiction-like criteria were considered nonaddicted, in agreement with our previous studies that considered addicted mice to be those that reached 2–3 criteria of addiction (8, 10). Similarly, in the DSM-5, a severe diagnosis for a substance use disorder does not require that 100% of the criteria be achieved, but rather 6 or more of the 11 criteria (i.e., 55% of the total criteria). Therefore, in our mouse model, we also considered a similar value of 66% of the total criteria to define the addicted phenotype (with 2 or more of the 3 criteria being considered positive).

### Data and materials availability.

All data are available in the main text or supplemental materials. Requests for materials should be addressed to co–corresponding author RM. The RNA-Seq data used in this study are available in the NCBI’s Sequence Read Archive (SRA) under accession number PRJNA817046 (SUB11224868; https://www.ncbi.nlm.nih.gov/bioproject/PRJNA817046/).

### Statistics.

All statistical comparisons were performed with SPSS software, version 25 (IBM). The comparison between 2 groups was analyzed with the Student’s *t* or Mann-Whitney *U* test, depending on the distribution defined by the Kolmogorov-Smirnov normality test. ANOVA with repeated measures was used when required to test the evolution over time with the subsequent post hoc analysis (Fisher’s least significant difference [LSD]). The relationship between the individual values of the 3 addiction-like criteria or the 4 addiction-related phenotypic traits and the final addiction criteria achieved were analyzed by Pearson’s correlation coefficient. The percentage of addicted mice compared with nonaddicted mice was analyzed with the χ^2^ test, and the observed frequencies with the frequencies obtained in the control group were compared. A *P* value of less than 0.05 was used to determine statistical significance. The sample sizes were similar to those reported in previous publications, with a power analysis superior to 80% (8).

### Study approval.

All animal procedures were conducted in strict accordance with the guidelines of the European Communities Council Directive 2010/63/E.U. and approved by the local ethics committee in Barcelona (Comitè Ètic d’Experimentació Animal-Parc de Recerca Biomèdica de Barcelona, CEEA-PRBB, agreement no. 9213). Maximal effort was made to reduce suffering and the number of mice used.

## Author contributions

EMG and RM conceived and designed the experimental approaches in the animal studies. JMFR conceived and designed the experimental approaches used in the human cohort. BC and NFC conceived the characterization of miRNA signatures. AB, AGB, and LDR performed the behavioral phenotype characterization with the supervision of EMG and RM. AGB, JCD, and LDR performed statistical analyses and generated graphs with the supervision of EMG and RM. JCD and LPC performed RNA extractions, small RNA-Seq, and bioinformatics analyses under the supervision of BC and NFC. SA performed DNA extractions and DNA library preparation for sequencing and analyses under the supervision of CS. AB and SA contributed to data analysis and interpretation of the results. JL and JMP performed the studies in humans with the supervision of JMFR. JEC contributed to bioinformatics analysis of behavioral data and miRNA expression in mice. EMG and RM wrote the manuscript and prepared the figures and tables with the support of AGB. BC, JCD, JMFR, and NFC provided critical review of the manuscript with input from all authors.

## Supplementary Material

Supplemental data

## Figures and Tables

**Table 3:**
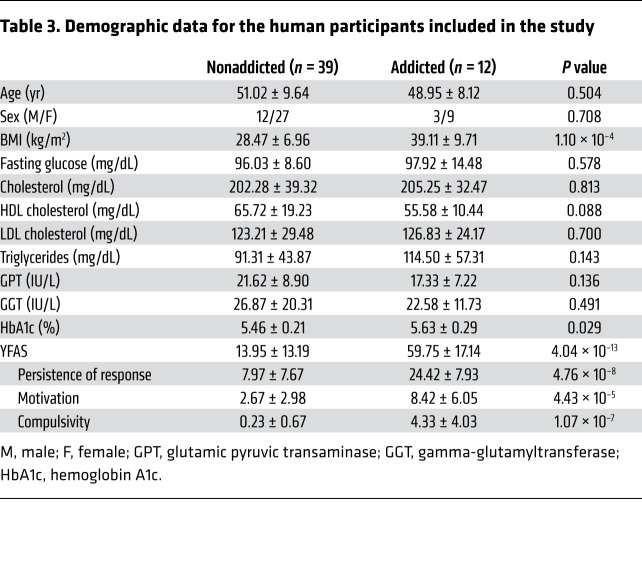
Demographic data for the human participants included in the study

**Table 2:**
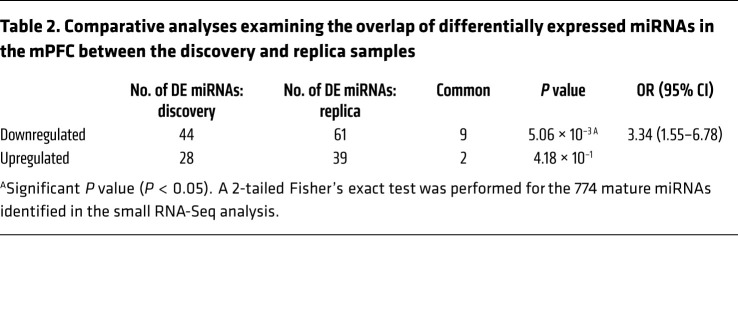
Comparative analyses examining the overlap of differentially expressed miRNAs in the mPFC between the discovery and replica samples

**Table 1:**
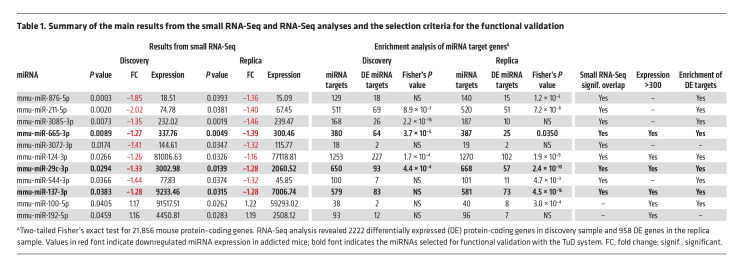
Summary of the main results from the small RNA-Seq and RNA-Seq analyses and the selection criteria for the functional validation

## Version 1 03/29/2022

In-Press Preview

## Version 2 05/16/2022

Electronic publication
