# Supplementary material for: miRNA signatures associated with vulnerability to food addiction in mice and humans
Source: J Clin Invest. 2022 May 16;132(10):e156281. doi: 10.1172/JCI156281 (PMC9106358; doi:10.1172/JCI156281)
Supplement: Supplemental data [file jci-132-156281-s205.pdf]

Supplemental Material for

MicroRNAs signatures associated with vulnerability to food addiction in mice and humans

Authors: Alejandra García-Blanco[1,†], Laura Domingo-Rodriguez[1,†], Judit Cabana-Domínguez[2,3,4,5,†], Noèlia Fernàndez-Castillo[2,3,4,5,†], Laura Pineda-Cirera[2,3,4,5], Jordi Mayneris-Perxachs[6,7,8], Aurelijus Burokas[1,9,], Jose Espinosa-Carrasco[10], Silvia Arboleya[11,12,‡], Jessica Latorre[6,7,8], Catherine Stanton[11,12], Bru Cormand[2,3,4,5], Jose Manuel Fernández-Real[6,7,8,13,*], Elena Martín-García[1,14,A,*,] Rafael Maldonado[1,14,A,*]

*Corresponding author. Email: rafael.maldonado@upf.edu
elena.martin@upf.edu
jmfreal@idibgi.org

Methods and materials

Animals

Experiments were performed in male JAX™ C57BL/6J wild-type (WT) mice, aged 8 weeks, purchased from Charles River (Lyon, France). Mice were housed and maintained individually in controlled laboratory conditions, the temperature at 21 ± 1ºC and humidity at 55 ± 10%, and food and water were available ad libitum during the entire experiment. Mice were tested during the dark phase of a reverse light cycle (lights off at 8.00 a.m and on at 8.00 p.m). Animal procedures were conducted in strict accordance with the guidelines of the European Communities Council Directive 2010/63/E.U. and approved by the local ethical committee (Comitè Ètic d'Experimentació Animal-Parc de Recerca Biomèdica de Barcelona, CEEA-PRBB, agreement N°9213). In agreement, maximal efforts were made to reduce the suffering and the number of mice used.

Experimental design

Mouse food addiction protocol. Mice were trained to obtain chocolate-flavored pellets (n=51) or standard pellets (n=7) in operant boxes during 98 sessions in 1 h-daily self-administration sessions. The operant boxes used and a daily self-administration session of the food addiction protocol was accurately described in (1, 2). Briefly, operant responding maintained by food was performed in mouse operant chambers (Model ENV-307A-CT, Med Associates, Georgia, VT, USA) equipped with two retractable levers, one randomly selected as the active lever and the other as the inactive. The grid floor is made of parallel metal bars and conducts the electric foot shock when it is scheduled.

Pressing on the active lever resulted in a pellet delivery together with a stimulus-light (associated-cue), located above the active lever, while pressing on the inactive lever had no consequences. The chambers were made of aluminum and acrylic and were housed in sound- and light-attenuated boxes equipped with fans to provide ventilation and white noise. A food dispenser equidistant between the two levers permitted delivery of food pellets when required. Mice were under fixed-ratio 1 (FR1) during six sessions, followed by 92 sessions under FR5. During this long operant conditioning maintained by food, the food addiction-like behavior was evaluated in 3 different time-points, an early period (1-14 sessions), a medium period (42-55 sessions), and a late period (82-92 sessions) to study the evolution of the addictive phenotype. The three food addiction-like criteria assessed were (1) persistence of response, (2) motivation, and (3) compulsive-like behavior, these criteria summarized the main features of the human food addiction diagnosis through the Yale Food Addiction Scale (YFAS 2.0) based on the 5th edition of the diagnostic and statistical manual of mental disorders (DSM-5) for substance use disorders. The three behavioral tests performed to evaluate these three addiction-like criteria were performed as described in detail (1–3).

At the end of the late period, we categorized animals in two subpopulations, and mice were classified as food-addicted or non-addicted depending on the number of addiction-like criteria achieved (1–3). Mice that achieved 2 or 3 addiction-like criteria were considered addicted animals, and mice with 0 or 1 addiction-like criteria were considered non-addicted animals.

We also evaluated 4 additional phenotypic traits during the food addiction protocol considered as factors of vulnerability to addiction to have a complete addictive-like

behavioral characterization (Figure S1B). We assessed impulsivity, cognitive flexibility, appetitive cue reactivity, and aversive cue reactivity (2, 4).

1. Impulsivity: The motor impulsivity, defined as the inability to stop a response once it is initiated (5), was measured considering the non-reinforced active responses during the time-out periods after each pellet delivery. A time-out period of 10 s was established after each pellet delivery, where the cue light was off, and no reinforcer was provided after responding on the active lever. In addition, the mean of the total active responses in the time-out periods of the 3 consecutive days before the progressive ratio test were considered.
2. Cognitive flexibility: The cognitive flexibility was measured in a reversal test. The reversal test was a regular training self-administration session, but the active and the inactive levers were reversed compared to the previous basal session. A mouse was considered to discriminate when at least 75% of the responses were on the same lever. The total number of inactive lever-presses in the basal session active responses was considered a measure of cognitive inflexibility.
3. Appetitive cue reactivity: The appetitive cue reactivity was measured with the cue-induced food-seeking test. The self-administration session lasted 90 min during this test and was divided into 2 periods: 60 min (extinction) + 30 min (cue-induced food-seeking) consecutive in 1 session. In the first 60 min extinction period, all lever-presses were not reinforced (active and inactive lever-presses had no scheduled consequences). In the second 30 min cue-induced food-seeking period, the white cue light, associated with pellet delivery during a regular self-administration session, was illuminated contingently according to an FR5 without pellet delivery. This period was initiated with the presentation of

the cue-light twice non-contingently and for 4 s. The increment of the active responses during the first 15 min of the second period, when the cue-light was presented, was considered a measure of appetitive cue reactivity. High active responses mean a high reactivity to the context (cue-light).

4. Aversive cue reactivity: The aversive cue reactivity was measured the day after the shock test using the shock context and not introducing the shock. In this test, mice were placed in the self-administration chamber for 50 min with the same grid floor used during the shock test. The mentioned grid floor is made of parallel metal bars and serves as a contextual cue because it is different from the floor of the regular FR5 sessions, without shock. In the regular FR5 sessions, a metal lamina with holes covers the grid floor and acts as a discriminative stimulus. In addition, during this session, pressing the active lever had no consequences: no shock, no chocolate-flavored pellets, and no cue-light. Therefore, the total non-reinforced active responses during this shock-context test were considered a measure of aversive cue reactivity. High active responses mean low cue-reactivity to the context (grid floor) associated with the aversive stimulus (shock).

Considering the individual score in each addiction-like criteria and the phenotypic traits associated with the disease, we selected some addicted and non-addicted mice with extreme phenotypes exposed to chocolate-flavored pellets to evaluate the differential miRNA epigenetic signature underlying the loss of control toward palatable food. For these studies, a specific brain area (medial prefrontal cortex, mPFC) was extracted immediately after the last FR5 session.

Candidate miRNAs functional validation. For the study of the inhibition of miR-29c-3p, miR-665-3p, and miR-137-3p in mPFC (specifically in the PL subregion), mice followed the same behavioral procedure described for the early period in the first experiment with some variations due to the surgical adeno-associated virus (AAV) injection (Fig. 5B). In particular, C57BL/6J WT mice were trained to acquire the operant conditioning maintained by chocolate-flavored pellets under FR1 (2 sessions) and FR5 (8 sessions) schedule of reinforcement followed by the surgery for injecting AAVs carrying the miRNA inhibitor (tough decoy (TuD) approach) (Fig. 5A). The TuD approach is one of the most potent approaches for miRNA inhibition strategies and provides the longest miRNA inhibition time compared with modified antimiRs and sponge decoy (6). TuDs are 100 nucleotides long and form a hairpin structure that increases their stability and contains an unpaired region in the middle. Both strands from this unpaired region are complementary to the target miRNA (6–8). Regarding miR-29c-3p inhibition, n=12 mice were injected with AAV-anti-mmu-miR-29c-3p TuD, and n=19 were injected with the AAV-control TuD; regarding miR-665-3p inhibition, n=19 mice were injected with AAV-anti-mmu-miR-665-3p TuD and n=18 were injected with the AVV-control TuD, and regarding miR-137-3p inhibition, n=16 mice were injected with AAV-control TuD and n=17 for AAV-anti-mmu-miR-665-3p TuD mice.

After bilateral intracranial injection of the AAV in the PL, the AAV expression was allowed for 4 weeks. After these 4 weeks, mice were under FR5 scheduled sessions followed by the measurement of the 3 addiction-like criteria and the subsequent classification of addicted and non-addicted mice. Mice were also tested for the 4 addiction-related phenotypic traits (Fig. S5, S6, and S7). After the last operant-conditioning session, animals were euthanized by decapitation, and brains were quickly removed and frozen

in methylbutane (previously stored at -80ºC overnight). Brains were cut in the cryostat at -20ºC. The prefrontal part of the brain (where PL, our area of interest, is located) was cut, alternating two different widths with obtaining different information (Fig. S4C). Brain slices of 30 µm were placed in a microscope slide (gelatinized KNITTEL StarFrost® slides 76 x 26 mm) embedded in mounting media (Fluromount-G™ with DAPI from Invitrogen) and covered with cover glass (deltalab 60 x 24 mm) to detect the presence of the AAV by GFP expression. Separately, in brain slices of 100 µm, mPFC were isolated by cutting meticulously with a scalpel. mPFC fragments of 11 mg were used for molecular confirmation by an RT-qPCR protocol explained below.

Food pellets

A standard pellet (20 mg, TestDiet, Richmond, IN, USA) or a chocolate-flavored pellet (20 mg, highly isocaloric pellet, TestDiet, Richmond, IN, USA) was delivered after pressing the active lever during the operant conditioning sessions. The standard pellets contain 24.1% protein, 10.4% fat, 65.5% carbohydrate with a caloric value of 3.30 kcal/g, while the chocolate-flavored pellets contain 20.6% protein, 12.7% fat, 66.7% carbohydrate with a caloric value of 3.44 kcal/g. Although both pellets presented a similar percentage of carbohydrates and caloric value, chocolate-flavored pellets included slight differences in their composition with an addition of chocolate flavor (2% pure unsweetened cocoa) and a higher proportion of sucrose content (50.1%) compared to standard pellets (8.3%). Indeed, although the carbohydrate content was similar in standard (65.5%) and highly palatable isocaloric pellets (66.8%), the proportion of sugars within this carbohydrate amount was different: sucrose content in standard pellets was 3.1% of the total carbohydrates and 50.1% in highly palatable pellets. These pellets were

presented only during the operant behavior sessions, and animals were maintained on standard chow for their daily food intake (1, 3, 9).

Drugs

To anesthetize mice during the surgical procedure to inject the AAV, an injection of ketamine hydrochloride (Imalgène; Merial Laboratorios S.A.) and medetomidine hydrochloride (Domtor; Esteve, Spain) mixed and dissolved in sterile 0.9% physiological saline (75 mg/kg and 1 mg/kg of body weight respectively) was intraperitoneally (i.p.) administered. After surgery, anesthesia was reversed by a subcutaneous (s.c.) injection of atipamezole hydrochloride (Revertor; Virbac, Spain; 2.5 mg/kg of body weight) dissolved in sterile 0.9% physiological saline. In addition, mice received an i.p. injection of gentamicin (Genta-Gobens; LaboratoriosNormon, Spain; 1 mg/kg of body weight) and a s.c. injection of meloxicam (Metacam; Boehringer Ingelheim, Rhein; 2 mg/kg of body weight) both dissolved in sterile 0.9% physiological saline.

Surgery and virus vector microinjection

Mice were anesthetized as described in the drugs section and placed into a stereotaxic apparatus for receiving the intracranial injections of the AAV-anti-miR-29c-3p TuD (Y4364 pAAV-CAG-eGFP-U6-TuD-RNA-mmumiR-29c-3p, 1.0E+12 gc/ml, from Obio Technology Corp., Ltd., Shanghai, China), the AAV-anti-miR-665 TuD (Y11816 pAAV-CAG-eGFP-U6-TuD-RNA-mmumiR-665-3p, 1.0+12 gc/mL, from Obio Technology Corp., Ltd. Shanghai, China), the AAV-anti-miR-137 TuD (Y11817 pAAV-CAG-eGFP-U6-TuD-RNA-mmumiR-137-3p, 2.0E+12 gc/ml, from Obio Technology Corp., Ltd. Shanghai, China) or

the AAV-control TuD (1.0E+12 gc/ml from Obio Technology Corp., Ltd. Shanghai, China) as a control. All the viruses are from Obio Technology Corp., Ltd. Shanghai, China. AAV-anti-miR-29c-3p TuD and AAV-control TuD have been tested before, and the effectiveness of the TuD inhibitors has been validated in (10). A detailed explanation step by step of the surgery was reported in (2). Briefly, the injections of the AAV were made through a bilateral injection cannula (33-gauge internal cannula, Plastics One, UK) connected to a polyethylene tubing (PE-20, Plastics One, UK) attached to a 10 µl microsyringe (Model 1701 N SYR, Cemented NDL, 26 ga, 2 in, point style 3, Hamilton company, NV). The displacement of an air bubble inside the length of the polyethylene tubing that connected the syringe to the injection needle was used to monitor the microinjections. The volume of 0.2 µl per site in the prelimbic (PL) area was injected at a constant rate of 0.05 µl/min by using a microinfusion pump (Harvard Apparatus, Holliston, MA) for 4 min. After infusion, the injection cannula was left in place for an additional period of 10 min to allow the fluid to diffuse and to prevent reflux, and then it was slowly withdrawn for 10 additional min. We applied the following coordinates to target the PL area according to Paxinos and Franklin (11): AP +1.98 mm, L ±0.3 mm, DV -2.3 mm.

To detect the viral expression of the AAVs, mice sacrificed at the end of the experiment (Fig. S4C), and the fluorescent reporter GFP was directly visualized in brain slices using a Leica DMR microscope equipped with a digital camera Leica DFC 300FX (10x objectives). Thus, GFP is a green fluorescent protein that was visible in our experimental conditions without performing immunofluorescence.

Principal component analysis

The principal component analysis (PCA) technique was used to evaluate the multidimensional data obtained in mice trained with chocolate-flavored pellets in the late period. PCA and varimax rotation were conducted using the 3 addiction-like criteria and the 4 phenotypic traits considered as factors of vulnerability to addiction and were dimensionality reduced to the minimum number of components that best explain and maximizes the variance present in the data set. An eigenvalue greater than 0.7 was set as the criterion for selecting components.

MicroRNA signatures in humans

Consecutive subjects from the IRONMET cohort with available YFAS 2.0 scores were recruited for this study (n=51) to analyze circulating miRNAs. The IRONMET cohort is a cross-sectional case-control study setting at the Endocrinology Department of Dr. Josep Trueta University Hospital (Table 3). The recruitment of subjects started in January 2016 and finished in October 2017. Consecutive middle-aged subjects, 27.2–66.6 years, were included. Patients with obesity (body mass index (BMI) ≥30 kg/m2) and age-matched and sex-matched subjects without obesity (BMI 18.5–<30 kg/m2) were eligible. Exclusion criteria were type 2 diabetes mellitus, chronic inflammatory systemic diseases, acute or chronic infections in the previous month; use of antibiotic, antifungal, antiviral or treatment with proton pump inhibitors; severe disorders of eating behavior or major psychiatric antecedents; neurological diseases, history of trauma or injured brain, language disorders and excessive alcohol intake (≥40 g OH/day in women or 80 g OH/day in men). The Institutional review board - Ethics Committee and the Committee for Clinical Research (CEIC) of Dr. Josep Trueta University Hospital (Girona, Spain) approved the study protocol and informed written consent was obtained from all participants.

Profile of Circulating miRNAs

Circulating RNA Extraction and Purification. Plasma was obtained by standard venipuncture and centrifugation using EDTA-coated Vacutainer tubes (Becton-Dickinson, Franklin Lakes, NJ). The separation of the plasma was performed by double-centrifugation using a laboratory centrifuge (Beckman J-6M Induction Drive Centrifuge, Beckman Instruments Inc., Palo Alto, CA). RNA extraction was performed from a starting volume of 300 µL of plasma using the mirVana PARIS Isolation Kit (Applied Biosystems, Darmstadt, Germany), according to manufacturer's instructions. Before RNA isolation, two synthetic oligonucleotides corresponding to miRNAs that do not exist in humans were spiked-in for quality control. These "exogenous" controls were synthesized to match the sequence of miRNAs of C. elegans, cel-miR-39 and cel-miR-54 (Qiagen, Gaithersburg, MD). The spiked-in oligonucleotides were introduced into the plasma sample as a mixture of 20 fmol in a 5 µL total volume of water and after addition of 2x Denaturing Solution. The final recovery of these synthetic oligonucleotides was measured for each sample using TaqMan qRTPCR miRNAs assays (Applied Biosystems, Darmstadt, Germany). To validate the success of each extraction, we also assessed the thermal cycle (Ct) values obtained for a serial dilution (10-1) of these miRNAs. Samples with recovery values less than approximately 50% were excluded.

Circulating miRNAs Retrotranscription and Preamplification. A fixed volume of 3 µL RNA solution from the 40-mL eluate of RNA isolation was used as input into the retrotranscription using the TaqMan miRNA Reverse Transcription kit (Life Technology, Darmstadt, Germany). Preamplification was performed using TaqMan PreAmp Master Mix (Life Technology, Darmstadt, Germany).

Analysis of Individual miRNAs Using TaqMan Hydrolysis Probes

Commercially available TaqMan hydrolysis probes (Applied Biosystems) were used to assess the presence in plasma of individual miRNAs. Gene expression was assessed by real-time PCR using the Light Cycler 480 Real-Time PCR System (Roche Diagnostics, Barcelona, Spain), using TaqMan technology suitable for relative gene expression quantification following the manufacturer's protocol. For the quantitative RT-PCR analysis, we performed the DeltaCt normalization using four well-known stable miRNAs in plasma (miR-106a, miR-146a, miR-19b and miR-223). The average of these four selected internal controls was used as a normalizing factor, as previously described (12). Ct values higher than 35 were excluded. TaqMan hydrolysis probes used were the following: hsa-miR-876-5p (002205), hsa-miR-211 (000514), hsa-miR-665 (002681), hsa-miR-29c (000587), hsa-miR-544 (002265), hsa-miR-137 (000593), hsa-miR-100 (000437), hsa-miR-192 (000491), hsa-miR-3085-3p (464290_mat), hsa-miR-124-3p (003188_mat), miR-106a (002169), miR-146a (000468), miR-19b (000396), miR-223 (002295), cel-miR-39 (000200) and cel-miR-54 (001361).

Yale Food Addiction Scale: The YFAS is a 35-item questionnaire that assesses addictive-like eating according to the diagnostic criteria for substance dependence (13, 14). The YFAS has been shown to have adequate psychometric properties (original validation study Cronbach alpha=0.86) (13). The tool includes two scoring options: a symptom score from 0–11 based on the DSM criteria for substance dependence (FA diagnosis if ≥3 symptoms are reported) and clinical impairment or distress.

In this study, we used the Spanish YFAS 2.0 that is also a 35-item self-report instrument that measured the presence or absence of food addiction symptoms based on the eleven diagnostic criteria for Substance-Related and Addictive Disorders (SRAD) in the DSM-5 adapted to the context of highly processed food (15). Each YFAS 2.0 question had eight frequency responses (0 = “never”, 7 = “every day”), and depending on this, the question threshold was met or not met. As specific YFAS 2.0 questions represented one DSM-5 SRAD criterion, if the sum of questions under one addiction criterion was ≥ 1, the addiction criterion was met and scored as 1. If the sum was 0, then the addiction criterion was not met and scored as 0. Finally, it was counted the number of addiction criteria met, and reaching two addiction criteria was sufficient to be considered food addicted if clinical significance impairment or distress were reported (15). Since certain YFAS 2.0 questions represented a specific DSM-5 addiction criterion and specific DSM-5 criteria were grouped in three addiction-like criteria, (1) persistence of response, (2) motivation, and (3) compulsive-like behavior, in the food addiction mouse model, each addiction-like criterion could be denoted by exact YFAS 2.0 questions as follows: persistence of response, YFAS 2.0 questions 1, 2, 3, 4, 25, 31 and 32; motivation, YFAS 2.0 questions 5, 6, 7, 8, 10, 18 and 20; compulsive-like behavior: YFAS 2.0 questions 22 and 23. In this study, the total question score for each addiction-like criterion in human graphs was the sum of the frequency mark obtained in each YFAS 2.0 question. The YFAS 2.0 was translated into Spanish according to the International Test Commission Guidelines for Translating and Adapting Tests by two bilingual clinical psychologists experts in eating disorders. The validated Spanish version YFAS 2.0 showed good psychometric properties with internal consistency, accuracy, discriminative and convergent validity with other eating-related constructs (16).

Human studies: Gene-based association analysis

GWAs summary statistics of BMI (that includes about ~700000 individuals) was obtained from the GIANT database (https://portals.broadinstitute.org/collaboration/giant/index.php/GIANT_consortium_data_files) (17). Gene-based association studies were performed on MAGMA v1.06 using the 1000 Genomes Project Phase 3 (European data only) as a reference panel. We used the SNP-wise mean model, which uses a statistic test of the sum of -log(SNP p-value) for SNPs located within the transcribed region. For miRNA, we selected all miRNA genes encoding each differentially expressed mature miRNA and the regulatory regions identified according to histone marks H3K4me1 and H3K27Ac (suggestive of enhancer regions) and H3K4me3 (associated with promoters) from the ENCODE regulation track in the UCSC Genome Browser (assembly GRCh37/hg19), as previously described (10). For target genes, we used NCBI 37.3 gene definitions without window around genes. We obtained a list of miRNAs target genes both validated (miRTarBase; http://mirtarbase.mbc.nctu.edu.tw/php/index.php) (18) and predicted (miRSystem; http://mirsystem.cgm.ntu.edu.tw/).

RNA extraction

Animals were euthanized by decapitation, and brains were quickly removed. The mPFC brain area was dissected following coordinates from Paxinos and Franklin (Paxinos, G. and Franklin, 2001): mPFC AP+1.98 mm. Samples were frozen by immersion in 2-methylbutane surrounded by dry ice and stored at −80°C for later quantification of

miRNA expression. According to the manufacturer's protocol, total RNA, including miRNA, was isolated from mPFC using the AllPrep DNA/RNA/miRNA Universal Kit (Qiagen Düsseldorf, Germany) according to the manufacturer's protocol and stored at -70ºC.

Library preparation and smallRNA sequencing

SmallRNA sequencing (smallRNA-seq) was performed by the Centre de Regulació Genòmica (CRG, Barcelona, Spain). RNA integrity was assessed by Agilent 2100 Bioanalyzer (Agilent Technologies, Santa Clara, CA, USA) and quantified through qPCR. According to the manufacturer, libraries were prepared with the NEBNext® Ultra™ Small R.N.A. Library Prep set for Illumina (New England Biolabs, Ipswich, MA, USA) 's protocol and sequenced 1x50 Illumina HiSeq2500 System for both the discovery and replica samples. The analysis of smallRNA-seq was carried out through the OASIS2 pipeline (http://oasis.dzne.de/)(19). Briefly, the quality of reads was inspected using FastQC v0.10.1(20), and data was cleaned using CutAdapt 1.7.1(21) to remove adapters, and low-quality reads, and only the reads between 15-32bp were kept. Next, reads were mapped against the *mus musculus* genome of reference (GRCm38/mm10) with STAR 2.4.1d(22), allowing 0 mismatches for reads of length 15-19bp and 1 mismatch for reads between 20-32bp. Finally, the differential expression analysis was done by DESeq2 (23). In order to discard those miRNAs with very low expression or sequencing errors, we only considered the mature miRNA showing at least five reads across all the samples. We selected those miRNAs showing nominal differential expression in mPFC, with concordant effects on gene expression in both the discovery and replica samples. We then used the two-tailed Fisher Exact test to determine the statistical significance of the

overlap observed between the discovery and replica samples for each brain area, considering up-and down-regulated genes separately. We only used those genes for further analysis, showing a statistical significance of the overlap observed between samples.

RNA sequencing

Sequencing of mRNAs (mRNAseq) from mPFC samples was also carried out by the CRG (Barcelona, Spain). RNA integrity was previously assessed by Bioanalyzer (see above) and quantified with a Qubit fluorometer. The KAPA Stranded mRNA-Seq Kit (Roche, Basel, Switzerland) was used to construct the libraries and sequenced on an Illumina HiSeq 3000/4000 with 75 bp paired end (PE) reads for the discovery sample and on an Illumina NovaSeq 6000 SP with 50 PE reads for the replica sample. RNAseq reads were cleaned of adapters and low-quality reads and mapped against the Mus musculus genome of reference (GRCm38/mm10) using STAR version 2.5.3a (Dobin et al., 2013) with ENCODE parameters. The annotated genes were quantified (Gencode version M18 and M24 for the discovery and replica sample, respectively) using RSEM version 1.3.0 (12) with default options. Differential expression between conditions was performed with DESeq2 version 1.18 (23) using the Wald test and correcting the results for multiple testing using the Benjamini and Hochberg method.

Prioritization of differentially expressed miRNAs

We filtered out those miRNAs with low expression considering baseMean<300. Then, we obtained a list of the miRNAs target genes both validated (miRTarBase;

http://mirtarbase.mbc.nctu.edu.tw/php/index.php) (18) and predicted (miRSystem; http://mirsystem.cgm.ntu.edu.tw/) (24). Then, we performed a two-tailed Fisher Exact test using data from mRNAseq of the same samples to identify the enrichment of the miRNA target genes among the differentially expressed protein-coding genes, separately for the discovery and replication sample. Finally, we search on the bibliography information about these miRNAs related to "addiction", "psychiatric disorders", and "obesity" to filter miRNA based on the novelty of the findings and to their possible therapeutic target.

Analysis of miRNA networks and miRNAs target genes

To inspect the relationships among the DE miRNAs and their target genes, we performed network analysis using the web-based platform miRNet (https://www.mirnet.ca/miRNet/home.xhtml)(25). We used the "Degree Filter" to retain more nodes with more connections. In addition, we used the "Function explorer" option to perform an analysis of KEGG pathways of all miRNA target genes using the default options. Finally, we used the lists of target genes of each miRNA to perform enrichment analysis on biological functions using Gene Ontology non-redundant Biological Process (GO) and Kyoto Encyclopedia of Genes and Genomes (KEGG) with the webtool WebGestAlt (WEB-based GEne SeT AnaLysis Toolkit, http://www.webgestalt.org/) (26).

RT-qPCR of miRNAs and target genes

Total RNA with miRNAs was extracted from samples of mice treated with AAV-anti-miR-665 TuD (n=19) and the AVV-control TuD (n=18) using the miRNeasy micro kit (Qiagen) according to the manufacturer's protocol. Changes in gene expression of miR-665-3p target genes: *Ncam1,* and *Rbfox1*, were analyzed. mRNA was retrotranscribed using the High Capacity cDNA Reverse Transcription kit (Roche Life Science, Branford, CT, USA) and quantified by RT-qPCR using the LightCycler® 480 SYBR Green I Master (Roche). In both cases, relative quantification was performed on LightCycler® 480 Software, Version 1.5 (Roche Life Science) by the comparative Ct (ΔΔCt) method using reference genes *Gapdh, Hprt1,* and *Pgk1*. Differences between conditions were evaluated with a Student t-test or U Mann-Whitney depending on the distribution defined by the Kolmogorov-Smirnov normality test using SPSS statistics software version 22.0.

References

1. Domingo-Rodriguez L et al. A specific prelimbic-nucleus accumbens pathway controls resilience versus vulnerability to food addiction. *Nat. Commun.* 2020;11(1):1–16.
2. Martín-García E, Domingo-Rodriguez L, Maldonado R. An operant conditioning model combined with a viral vector approach to study the neurobiology of food addiction in mice. *Bio-protocol* 2020;10(19):1–23.
3. Mancino S et al. Epigenetic and Proteomic Expression Changes Promoted by Eating Addictive-Like Behavior.. *Neuropsychopharmacology* 2015;40(12):2788–800.
4. Dimet AL et al. A protocol for measuring cue reactivity in a rat model of cocaine use disorder. *J. Vis. Exp.* 2018;2018(136):1–8.
5. Koob GF, Volkow ND. Neurocircuitry of addiction. *Neuropsychopharmacology* 2010;35(1):217–238.
6. Hollensen AK et al. Enhanced Tailored MicroRNA Sponge Activity of RNA Pol II-Transcribed TuD Hairpins Relative to Ectopically Expressed ciRS7-Derived circRNAs. *Mol. Ther. - Nucleic Acids* 2018;13(December):365–375.
7. Bak RO, Hollensen AK, Primo MN, Sørensen CD. Potent microRNA suppression by RNA Pol II-transcribed 'Tough Decoy' inhibitors2013;280–293.
8. Maldonado R et al. Genomics and epigenomics of addiction. *Am. J. Med. Genet.* 2021;https://doi.org/10.1002/ajmg.b.32843.
9. Burokas A et al. Extinction and reinstatement of an operant responding maintained by food in different models of obesity. *Addict. Biol.* 2018;23(2):544–555.
10. Su H et al. Regulation of microRNA-29c in the nucleus accumbens modulates

methamphetamine -induced locomotor sensitization in mice. *Neuropharmacology* 2019;148(January):160–168.
11. Paxinos, G. and Franklin KBJ. *The Mouse Brain in Stereotaxic Coordinates*. Academic Press, San Diego: 2001:
12. Li B, Dewey C. Assembly of non-unique insertion content using next-generation sequencing. *RSEM accurate Transcr. Quantif. from RNA-Seq data with or without a Ref. genome* 2011;21–40.
13. Gearhardt AN, Corbin WR, Brownell KD. Preliminary validation of the Yale Food Addiction Scale. *Appetite* 2009;52(2):430–436.
14. Schulte EM, Gearhardt AN. Development of the Modified Yale Food Addiction Scale Version 2.0. *Eur. Eat. Disord. Rev.* 2017;25(4):302–308.
15. Gearhardt AN, Corbin WR, Brownell KD. Development of the Yale Food Addiction Scale Version 2.0.. *Psychol. Addict. Behav.* 2016;30(1):113–121.
16. Granero R et al. Validation of the Spanish version of the Yale Food Addiction Scale 2.0 (YFAS 2.0) and clinical correlates in a sample of eating disorder, gambling disorder, and healthy control participants. *Front. Psychiatry* 2018;9(MAY):1–11.
17. Yengo L et al. Meta-analysis of genome-wide association studies for height and body mass index in ∼700000 individuals of European ancestry.. *Hum. Mol. Genet.* 2018;27(20):3641–3649.
18. Chou C-H et al. miRTarBase update 2018: a resource for experimentally validated microRNA-target interactions. *Nucleic Acids Res.* 2018;46(D1):D296–D302.
19. Rahman R-U et al. Oasis 2: improved online analysis of small RNA-seq data. *BMC Bioinformatics* 2018;19(1):54.
20. Andrews S. FastQC: A quality control tool for high throughput sequence data.2010;
21. Martin M. Cutadapt removes adapter sequences from high-throughput sequencing reads. *EMBnet.journal* 2011;17(1):10.
22. Dobin A et al. STAR: Ultrafast universal RNA-seq aligner. *Bioinformatics* 2013;29(1):15–21.
23. Love MI, Huber W, Anders S. Moderated estimation of fold change and dispersion for RNA-seq data with DESeq2. *Genome Biol.* 2014;15(12):550.
24. Lu T-P et al. miRSystem: An Integrated System for Characterizing Enriched Functions and Pathways of MicroRNA Targets. *PLoS One* 2012;7(8):e42390.
25. Fan Y et al. miRNet - dissecting miRNA-target interactions and functional associations through network-based visual analysis. *Nucleic Acids Res.* 2016;44(W1):W135–W141.
26. Liao Y, Wang J, Jaehnig EJ, Shi Z, Zhang B. WebGestalt 2019: gene set analysis toolkit with revamped UIs and APIs. *Nucleic Acids Res.* 2019;47(W1):W199–W205.

Figures and legends

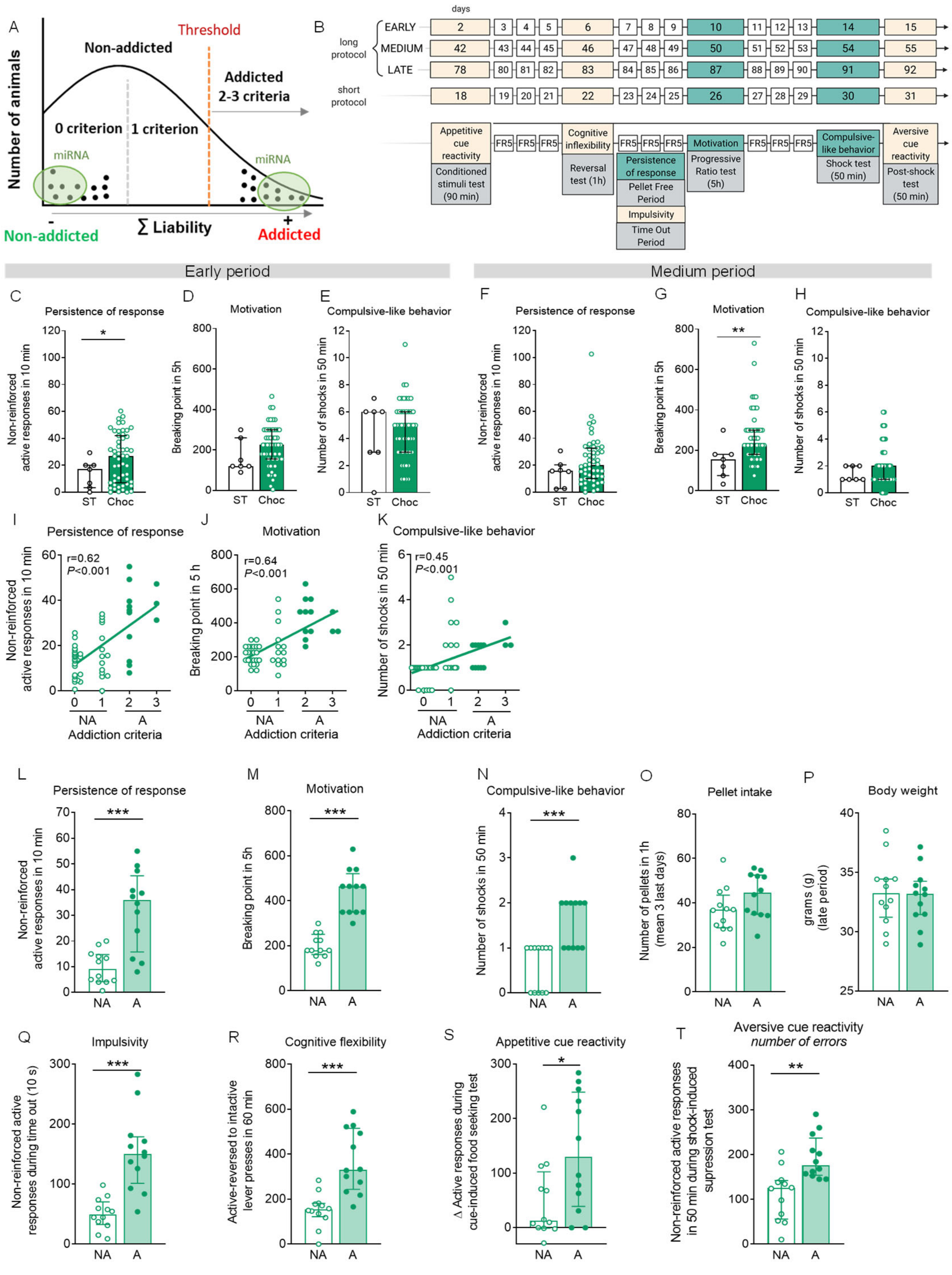

Fig. S1

Fig. S1. Complementary characterization of two extreme subpopulations of addicted and non-addicted mice obtained by operant training with chocolate-flavored pellets. **(A)** Inverted U-shaped curve showing that operant training with chocolate-flavored

pellets allows distinguishing extreme subpopulations of addicted and non-addicted mice. **(B)** Timeline of the experimental sequence for the long and short protocols indicating the specific time each test was performed. **(C-E)** Behavioral tests for the 3 addiction-like criteria in the early period expressed by individual values with the median and interquartile range. **(C)** persistence of response (t-test, *P<0.05) **(D)** motivation, **(E)** compulsive-like behavior. **(F-H)** Behavioral tests for the 3 addiction-like criteria in the medium period expressed by individual values with the median and interquartile range. **(F)** persistence of response, **(G)** motivation (U Mann-Whitney ** P<0.01), **(H)** compulsive-like behavior. **(I-K)** Pearson correlations between individual addiction-like criteria and **(I)** non-reinforced active responses in 10 min (r=0.62, P<0.001), **(J)** breaking point in 5 h (r=0.64, P<0.001), **(K)** number of shocks in 50 min (r=0.45, P<0.001). Behavioral tests of the three addiction-like criteria **(L-N)** and pellets intake **(O), and** body weight **(P),** in daddition to the 4 additional factors of vulnerability to addiction **(Q-T)** for those mice selected for the epigenetic study in addicted ("A") and non-addicted ("NA") mice trained with chocolate pellets. **(L)** persistence of response (U Mann-Whitney *** P< 0.001). **(M)** Motivation (t-test *** P< 0.001). **(N)** Compulsive-like behavior (U Mann-Whitney *** P< 0.001), **(O)** Pellets intake in the last FR5 three sessions, **(P)** body weight in the late period, **(Q)** Impulsivity (t-test *** P< 0.001), **(R)** Cognitive flexibility (t-test *** P< 0.001), **(S)** appetitive cue-reactivity (t-test * P< 0.05), **(T)** aversive cue-reactivity (U Mann-Whitney ** P< 0.01) (n=12 A mice, n=12 NA mice; individual values with the median and the interquartile range). Statistical details are included in Table S10.

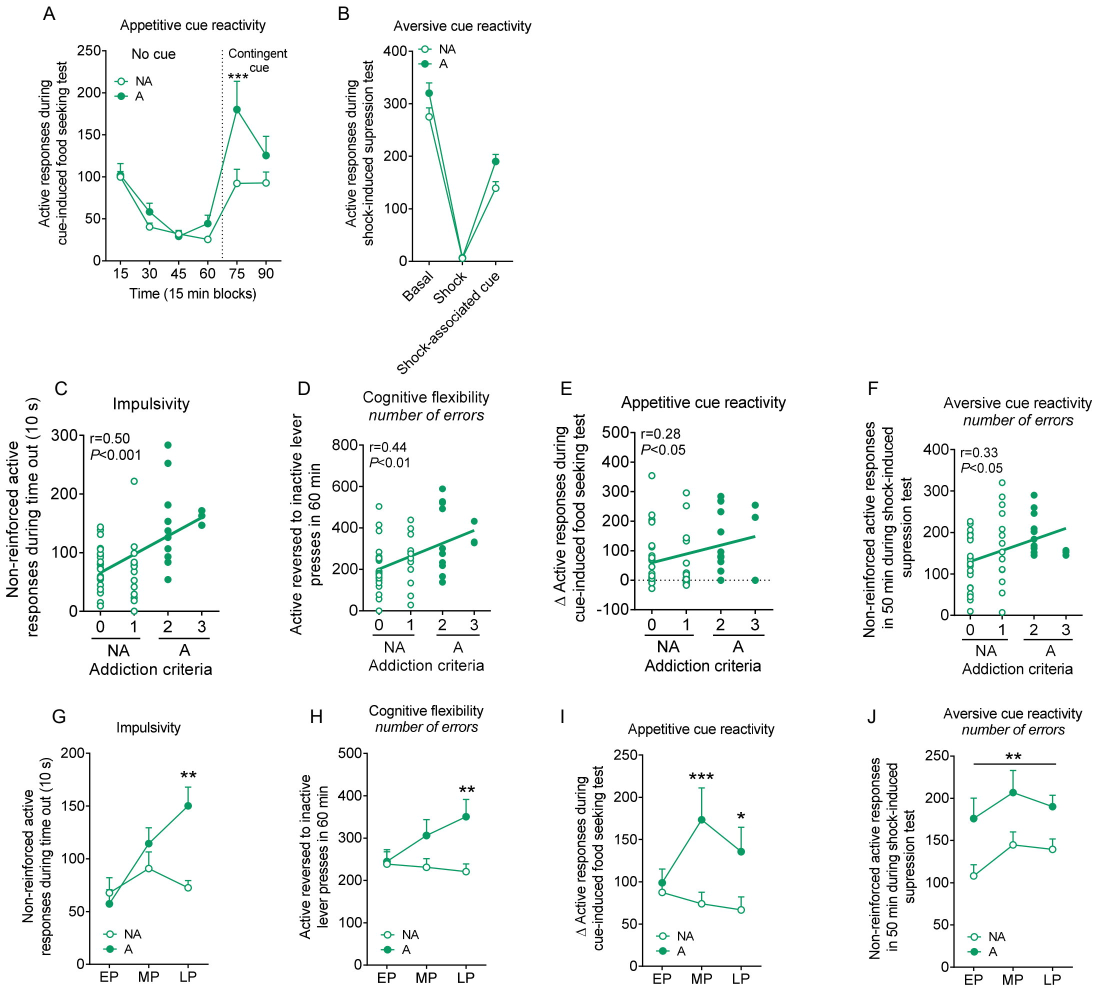

Fig. S2. Characterization of phenotypic traits related to addiction in two extreme subpopulations of addicted (A) and non-addicted mice (A). Appetitive cue-reactivity measured by the number of active responses during 90-min cue-induced food-seeking test (mean ± SEM; repeated measures ANOVA; time x addiction categorization P<0.01, Fisher LSD posthoc analysis, ***P<0.001**). (B)** The number of active responses measures aversive cue-reactivity associative learning during the shock-induced suppression test. **(C-F)** Pearson correlations between individual addiction-like criterion and **(C)** impulsivity (r=0.50, P<0.001); **(D)** cognitive flexibility (r=0.44, P<0.01); **(E)** appetitive cue-reactivity (r=0.28, P<0.05) and **(F)** aversive cue-reactivity (r=0.33, P<0.05). **(G-J)** Evolution over early, medium and late periods of **(G)**, impulsivity (mean ± SEM; repeated measures ANOVA; periods effect P<0.01, periods x addictive phenotype P<0.05, Fisher LSD post

hoc analysis, **P<0.01), **(H)** cognitive flexibility (mean ± SEM; repeated measures ANOVA; periods x addiction categorization P<0.05, Fisher LSD post hoc analysis, **P<0.01), **(I)** appetitive cue-reactivity (mean ± SEM; repeated measures ANOVA, Fisher LSD post hoc analysis, *P<0.05, ***P<0.001) and **(J)** aversive cue-reactivity (mean ± SEM; repeated measures ANOVA; addiction categorization effect, **P<0.01). Statistical details are included in Table S10.

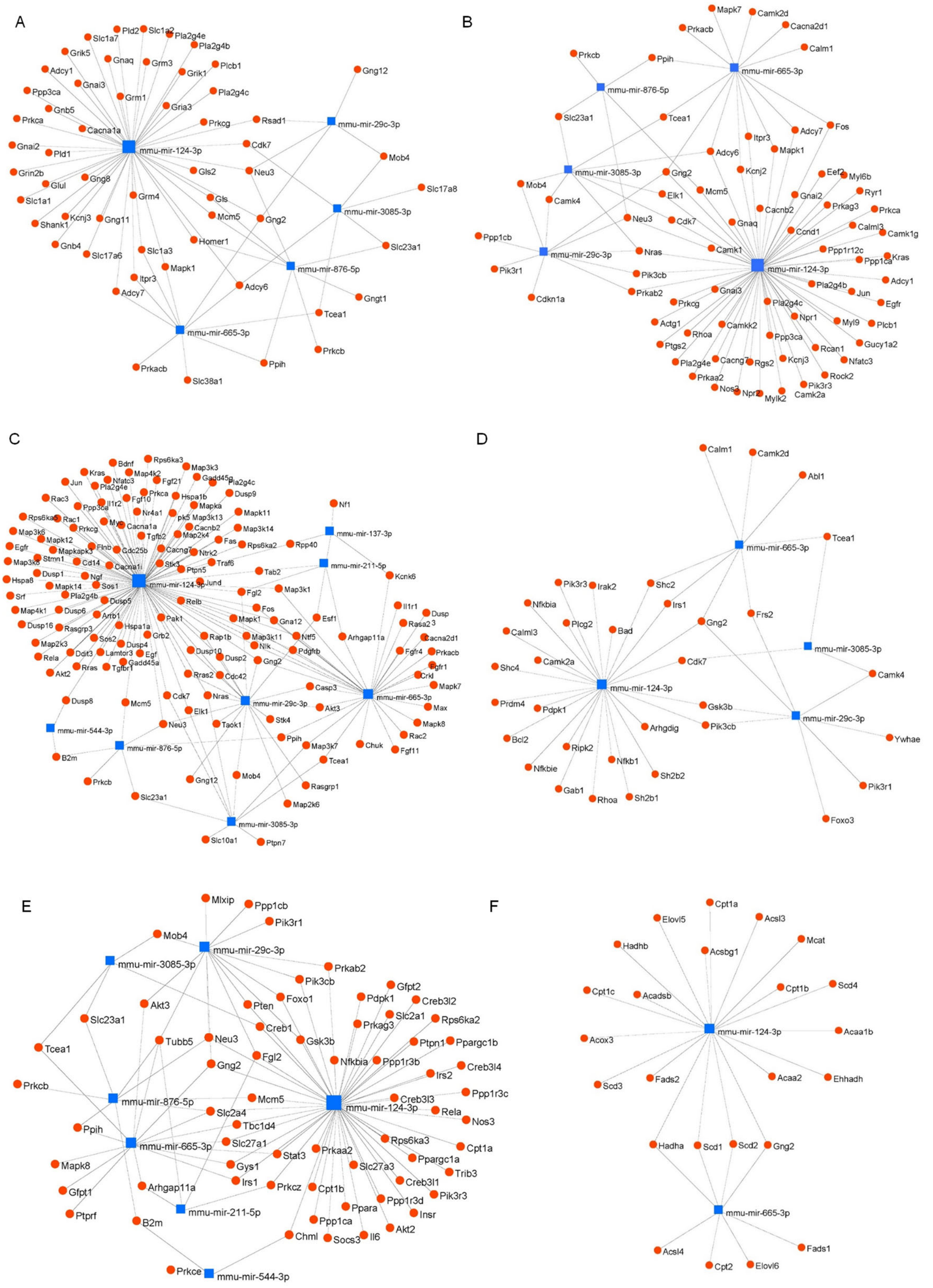

Fig. S3. Networks of the selected KEGG pathways enriched among the downregulated miRNAs and their target genes. Results of networks from miRNet analysis based on the KEGG pathways **(A)** glutamatergic synapse, **(B)** oxytocin signaling pathway, **(C)** MAPK

signaling pathway, **(D)** neurotrophin signaling pathway, **(E)** insulin resistance, and **(F)** fatty acid metabolism.

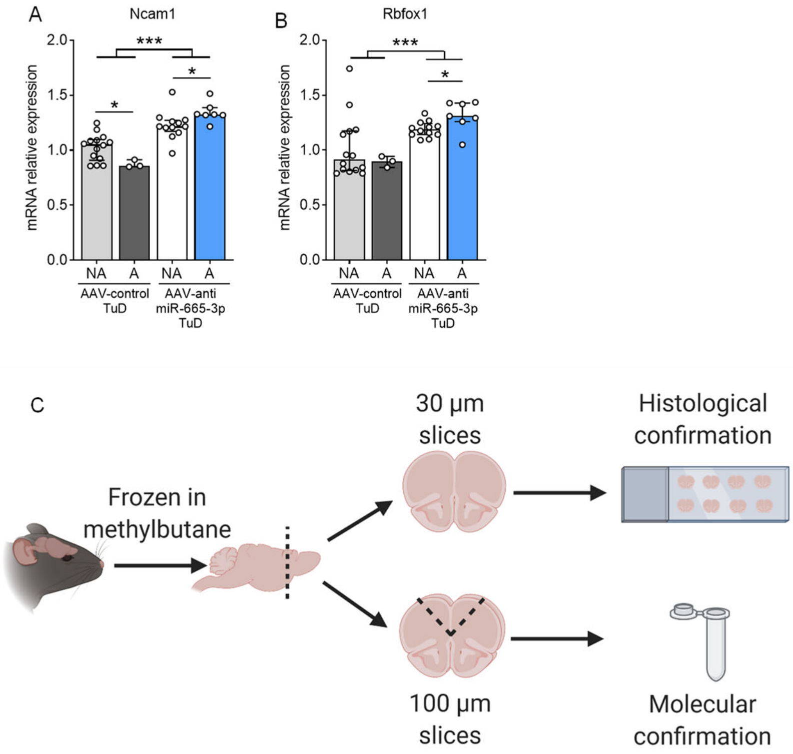

Fig. S4. Detection of mmu-miR-665-3p target genes in PL mPFC. (A) *Ncam1* and **(B)** *Rbfox1* were significantly upregulated in AVV-anti mmu-miR-665-3p TuD mice, and the expression of these genes was also higher in addicted AAV-anti-mmu-miR-665-3p TuD mice than non-addicted mice. **(C)** Histological verification of correct AAV injection. After behavioral experiments, mice were sacrificed, and brain tissue was rapidly dissected, immediately frozen in methylbutane and dry ice, and stored at −80 °C until used. To detect the viral expression in all the experiments, the GFP reporter was visualized directly in the fluorescence microscope in coronal slices of 30 µm. We also performed qPCR analysis for molecular analysis using interleaved coronal slices of 100 µm. Statistical details are included in Table S13.

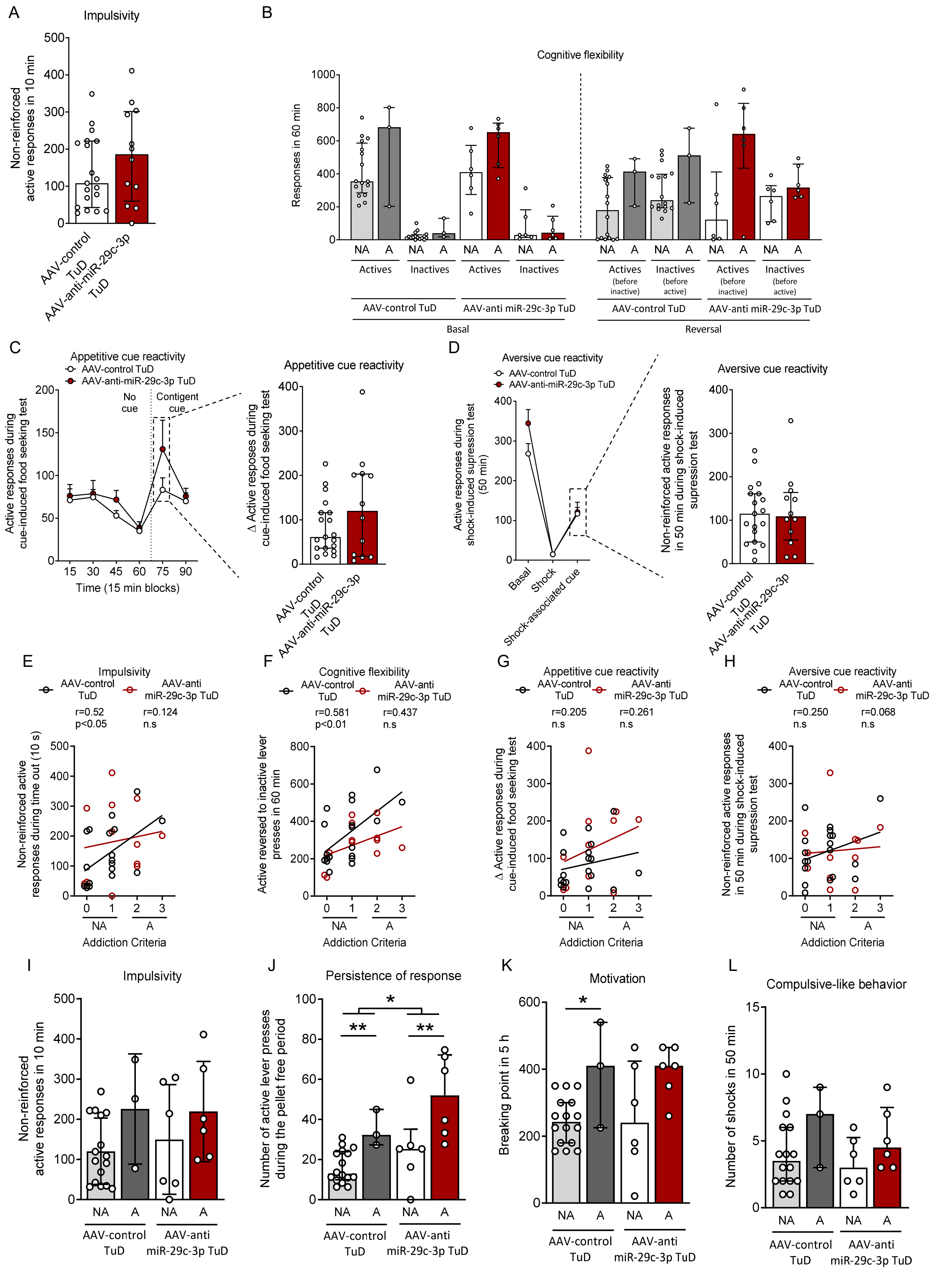

Fig. S5. Characterization of phenotypic traits related to addiction in AAV-anti-mmu-miR-29c-3p TuD mice (A-D). Behavioral test of AAV-anti-mmu-miR-29c-3p TuD mice regarding four phenotypic traits related to addiction. **(A)** Impulsivity. **(B)** Cognitive flexibility. **(C)** Appetitive cue-reactivity and **(D)** aversive cue-reactivity. **(E-H)** Pearson

correlations between individual addiction-like criteria and **(E)** impulsivity. **(F)** cognitive flexibility. **(G)** Appetitive cue-reactivity. **(H)** Aversive cue-reactivity. **(I-L)** Behavioral tests of impulsivity **(I)** and the three addiction-like criteria **(J)** persistence of response, **(K)** motivation, and **(L)** compulsive-like behavior in non-addicted ("NA") and addicted ("A") mice in both genotypes. Individual values and bars with median and the interquartile range (n=16 for Non-Addicted AAV-control TuD; n=3 for Addicted AAV-control TuD; n=6 for Non-Addicted AAV-anti-mmu-miR-29c-3p TuD mice and n=6 for AAV-anti-mmu-miR-29c-3p TuD mice). Statistical details are included in Table S12.

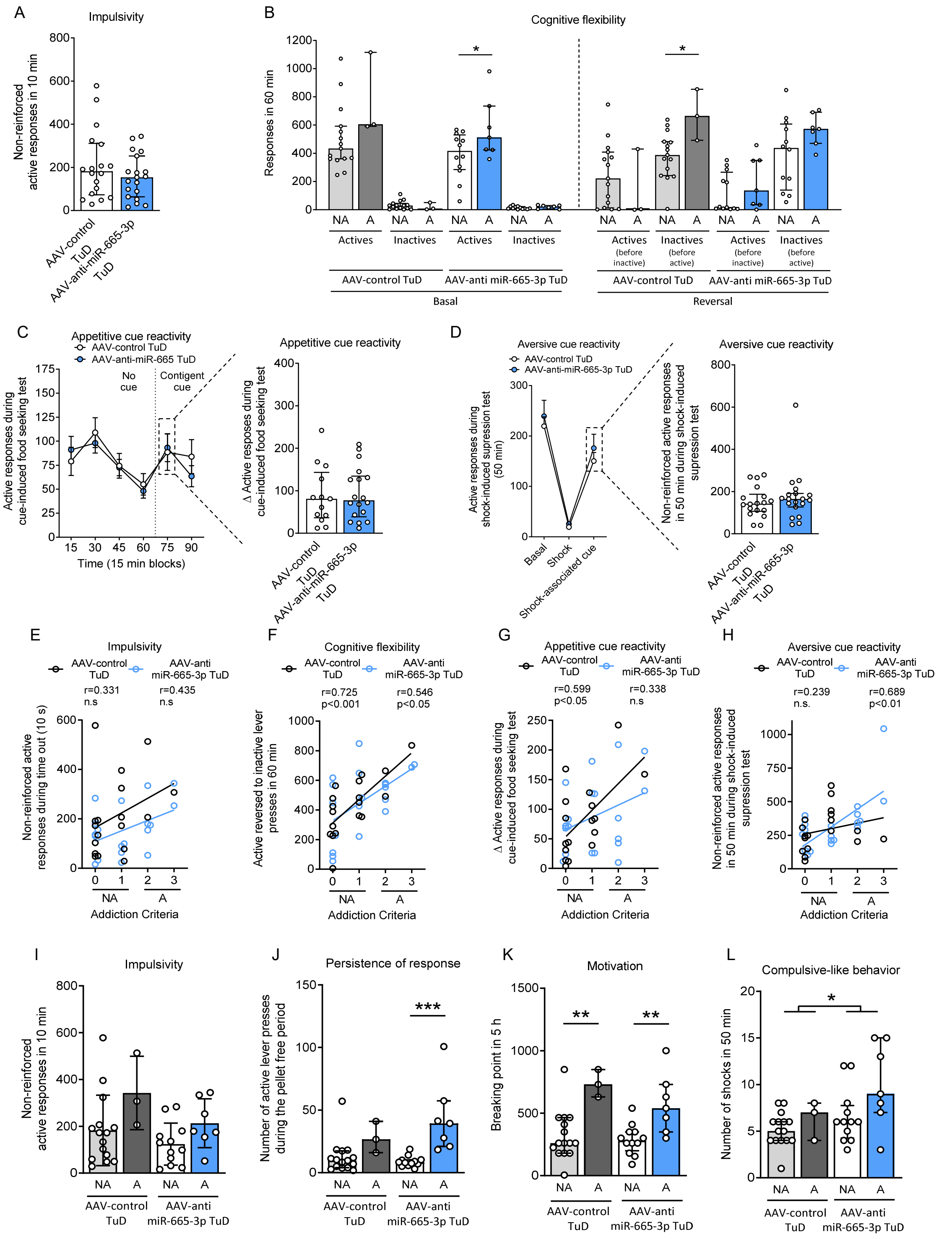

Fig. S6. Characterization of phenotypic traits related to addiction in AAV-anti-mmu-miR-665-3p TuD mice (A-D). Behavioral test of AAV-anti-mmu-miR-665-3p TuD mice regarding five phenotypic traits related to addiction. **(A)** Impulsivity. **(B)** Cognitive flexibility. **(C)** Appetitive cue-reactivity. **(D)** Aversive cue-reactivity. **(E-H)** Pearson

correlations between individual addiction-like criteria and **(E)** impulsivity, **(F)** cognitive flexibility, **(G)** appetitive cue-reactivity and **(H)** aversive cue-reactivity. **(I-L)** Behavioral tests of **(I)** impulsivity and the three addiction-like criteria **(J)** persistence of response, **(K)** motivation, and **(L)** compulsive-like behavior in non-addicted (NA) and addicted (A) mice in both genotypes. Individual values and bars with median and the interquartile range. (n=15 for Non-Addicted AAV-control TuD; n=3 for Addicted AAV-control TuD; n=12 for Non-Addicted AAV-anti-miR-665-3p TuD mice and n=7 for Addicted AAV-anti-miR-665-3p TuD mice). Statistical details are included in Table S13.

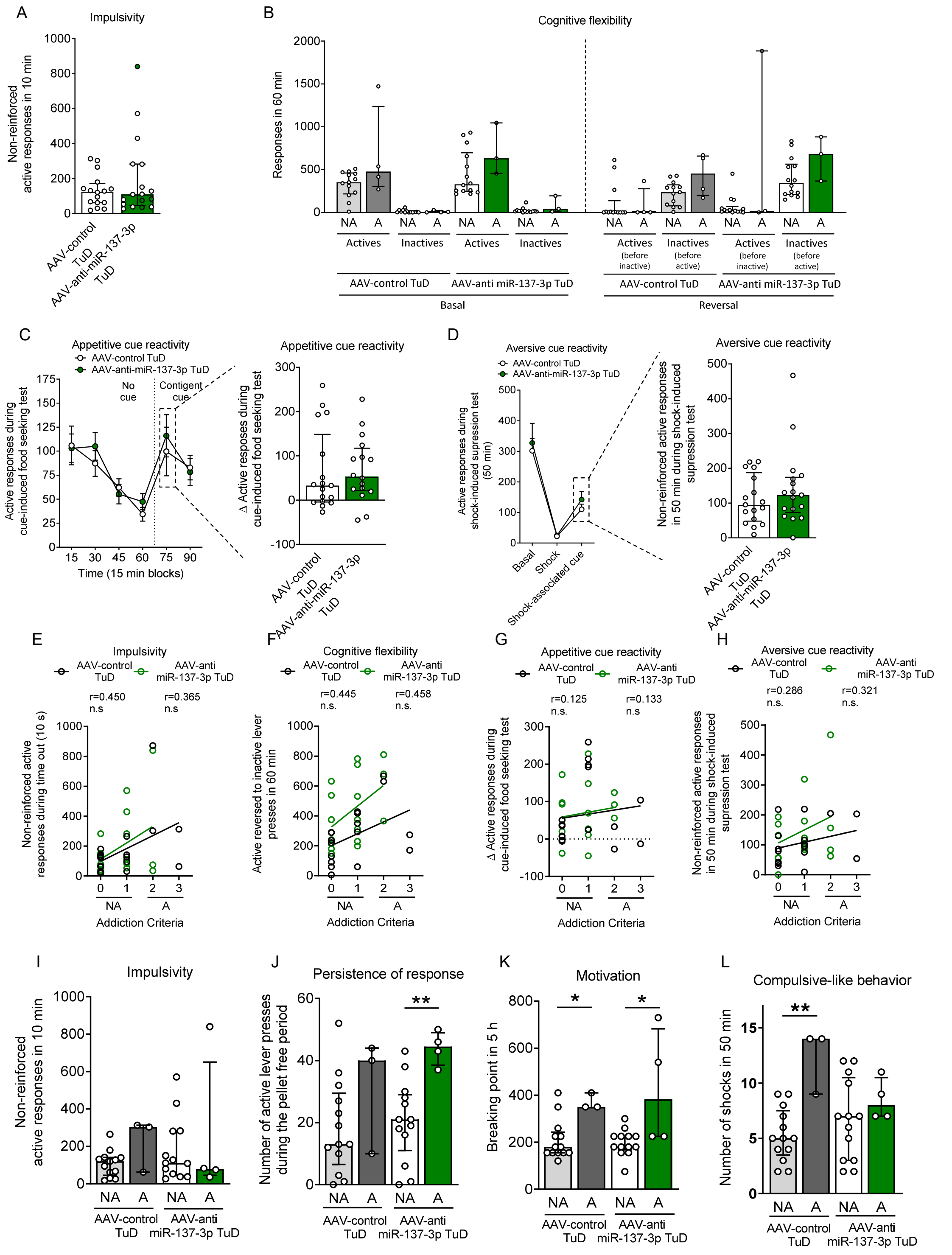

Fig. S7. **Characterization of phenotypic traits related to addiction in AAV-anti-mmu-miR-137-3p TuD mice (A-D).** Behavioral test of AAV-anti-mmu-miR-137-3p TuD mice regarding four phenotypic traits related to addiction. **(A)** Impulsivity. **(B)** Cognitive flexibility. **(C)** Appetitive cue-reactivity. **(D)** Aversive cue-reactivity. **(E-H)** Pearson

correlations between individual addiction-like criteria and impulsivity, **(F)** cognitive flexibility, **(G)** appetitive cue-reactivity, and **(H)** aversive cue-reactivity. **(I-L)** Behavioral tests of **(I)** impulsivity and the three addiction-like criteria **(J)** persistence of response, **(K)** motivation, and **(L)** compulsive-like behavior in non-addicted (NA) and addicted (A) mice in both genotypes. Individual values and bars with median and the interquartile range. (n=13 for Non-Addicted AAV-control TuD; n=3 for Addicted AAV-control TuD; n=13 for Non-Addicted AAV-anti-miR-137-3p TuD mice and n=4 for Addicted AAV-anti-miR-137-3p TuD mice). Statistical details are included in Table S14.

Table S1. Differences in miRNA expression in the mPFC between vulnerable and resilient mice from the discovery sample.

Mature	Chromosome	Start	Stop	Strand	Sequence	log2Fold Change	baseMean	baseMean _control	baseMean _treatment _files_1	lfcSE	Stat	Pvalue	Padj FDR
**mmu-miR-34c-3p**	chr9	51103044	51103065	-	AAUCACUAACCACACAGCCAGG	0.93	112.81	70.21	155.41	0.25	3.67	2.40E-04	0.0991
**mmu-miR-876-5p**	chr4	36645422	36645443	-	UGGAUUUCUCUGUGAAUCACUA	-0.89	18.51	25.15	11.88	0.24	-3.66	2.56E-04	0.0991
**mmu-miR-34b-3p**	chr9	51103574	51103595	-	AAUCACUAACUCCACUGCCAUC	0.81	325.18	216.72	433.64	0.26	3.15	1.63E-03	0.2238
**mmu-miR-218-2-3p**	chr11	35616882	35616903	+	CAUGGUUCUGUCAAGCACCGCG	-0.55	63.80	76.93	50.68	0.17	-3.12	1.82E-03	0.2238
**mmu-miR-211-5p**	chr7	64205831	64205852	+	UUCCCUUUGUCAUCCUUUGCCU	-1.01	74.78	121.20	28.37	0.33	-3.08	2.05E-03	0.2238
**mmu-miR-872-5p**	chr4	94665167	94665187	+	AAGGUUACUUGUUAGUUCAGG	0.29	5410.54	4853.32	5967.75	0.09	3.08	2.07E-03	0.2238
**mmu-miR-140-5p**	chr8	1.08E+08	1.08E+08	+	CAGUGGUUUUACCCUAUGGUAG	0.33	2407.84	2124.48	2691.19	0.11	3.03	2.44E-03	0.2238
**mmu-miR-376a-3p**	chr12	1.1E+08	1.1E+08	+	AUCGUAGAGGAAAAUCCACGU	-0.54	512.10	615.59	408.62	0.18	-2.98	2.89E-03	0.2238
**mmu-miR-137-5p**	chr3	1.18E+08	1.18E+08	+	ACGGGUAUUCUUGGGUGGAUAAU	-0.40	141.45	161.95	120.95	0.14	-2.96	3.12E-03	0.2238
**mmu-miR-128-1-5p**	chr1	1.28E+08	1.28E+08	+	CGGGGCCGUAGCACUGUCUGA	-0.38	447.22	508.58	385.87	0.13	-2.94	3.27E-03	0.2238
**mmu-miR-6538**	chr12	26414970	26414988	-	CGCGGGCUCCGGGGCGGCG	0.75	126.60	87.80	165.39	0.25	2.94	3.31E-03	0.2238
**mmu-miR-329-5p**	chr12	1.1E+08	1.1E+08	+	AGAGGUUUUCUGGGUCUCUGUU	-0.25	8487.62	9238.33	7736.91	0.09	-2.92	3.47E-03	0.2238
**mmu-miR-200a-5p**	chr4	1.56E+08	1.56E+08	-	CAUCUUACCGGACAGUGCUGGA	-0.90	53.50	92.42	14.58	0.32	-2.85	4.42E-03	0.2264
**mmu-miR-200b-5p**	chr4	1.56E+08	1.56E+08	-	CAUCUUACUGGGCAGCAUUGGA	-0.83	33.14	61.59	4.69	0.29	-2.82	4.86E-03	0.2264
**mmu-miR-582-3p**	chr13	1.09E+08	1.09E+08	+	UAACCUGUUGAACAACUGAAC	-0.24	2530.02	2739.95	2320.09	0.08	-2.80	5.07E-03	0.2264
**mmu-miR-126a-3p**	chr2	26591402	26591423	+	UCGUACCGUGAGUAAUAAUGCG	0.28	54193.49	48777.65	59609.33	0.10	2.73	6.25E-03	0.2264
**mmu-miR-200a-3p**	chr4	1.56E+08	1.56E+08	-	UAACACUGUCUGGUAACGAUGU	-0.81	6689.50	12115.47	1263.53	0.30	-2.73	6.36E-03	0.2264
**mmu-miR-182-5p**	chr6	30165962	30165986	-	UUUGGCAAUGGUAGAACUCACACCG	-0.80	2973.41	5424.59	522.23	0.30	-2.72	6.53E-03	0.2264
**mmu-miR-96-5p**	chr6	30169506	30169528	-	UUUGGCACUAGCACAUUUUUGCU	-0.78	349.73	651.72	47.73	0.29	-2.70	6.93E-03	0.2264
**mmu-miR-10b-5p**	chr2	74726074	74726096	+	UACCCUGUAGAACCGAAUUUGUG	0.81	209.23	128.90	289.56	0.30	2.70	6.93E-03	0.2264
**mmu-miR-34b-5p**	chr9	51103610	51103632	-	AGGCAGUGUAAUUAGCUGAUUGU	0.74	350.86	239.21	462.50	0.27	2.69	7.11E-03	0.2264
**mmu-miR-3085-3p**	chr19	42280095	42280115	-	UCUGGCUGCUAUGGCCCCCUC	-0.44	232.02	269.62	194.43	0.16	-2.68	7.31E-03	0.2264
**mmu-miR-467e-5p**	chr2	10505731	10505752	+	AUAAGUGUGAGCAUGUAUAUGU	0.50	43.16	35.11	51.21	0.19	2.68	7.39E-03	0.2264
**mmu-miR-674-5p**	chr2	1.17E+08	1.17E+08	+	GCACUGAGAUGGGAGUGGUGUA	-0.32	724.23	806.81	641.65	0.12	-2.67	7.57E-03	0.2264
**mmu-miR-429-3p**	chr4	1.56E+08	1.56E+08	-	UAAUACUGUCUGGUAAUGCCGU	-0.80	749.37	1336.58	162.16	0.30	-2.67	7.66E-03	0.2264
**mmu-miR-184-3p**	chr9	89802263	89802284	-	UGGACGGAGAACUGAUAAGGGU	-0.66	499.76	634.17	365.35	0.25	-2.66	7.87E-03	0.2264
**mmu-miR-21a-5p**	chr11	86584120	86584141	-	UAGCUUAUCAGACUGAUGUUGA	0.45	79349.18	66221.97	92476.39	0.17	2.63	8.48E-03	0.2264
**mmu-miR-15b-3p**	chr3	69009813	69009834	+	CGAAUCAUUAUUUGCUGCUCUA	0.78	19.39	12.38	26.40	0.30	2.62	8.86E-03	0.2264
**mmu-miR-665-3p**	chr12	1.1E+08	1.1E+08	+	ACCAGGAGGCUGAGGUCCCU	-0.34	337.76	379.45	296.07	0.13	-2.61	8.93E-03	0.2264
**mmu-miR-183-5p**	chr6	30169711	30169732	-	UAUGGCACUGGUAGAAUUCACU	-0.76	2435.61	4422.68	448.55	0.29	-2.61	9.08E-03	0.2264

**mmu-miR-200c-3p**	chr6	1.25E+08	1.25E+08	-	UAAUACUGCCGGGUAAUGAUGGA	-0.75	406.70	743.48	69.91	0.29	-2.60	9.32E-03	0.2264
**mmu-miR-27a-3p**	chr8	84208727	84208747	+	UUCACAGUGGCUAAGUUCCGC	0.37	8858.64	7686.93	10030.36	0.14	2.60	9.36E-03	0.2264
**mmu-miR-200b-3p**	chr4	1.56E+08	1.56E+08	-	UAAUACUGCCUGGUAAUGAUGA	-0.79	2581.44	4435.06	727.83	0.31	-2.55	1.08E-02	0.2459
**mmu-miR-375-3p**	chr1	74900661	74900682	-	UUUGUUCGUUCGGCUCGCGUGA	0.58	821.61	633.42	1009.80	0.23	2.55	1.08E-02	0.2459
**mmu-miR-185-5p**	chr16	18327438	18327459	-	UGGAGAGAAAGGCAGUUCCUGA	-0.18	24353.19	25858.06	22848.33	0.07	-2.48	1.31E-02	0.2897
**mmu-miR-34c-5p**	chr9	51103076	51103098	-	AGGCAGUGUAGUUAGCUGAUUGC	0.71	17937.55	12110.02	23765.09	0.29	2.45	1.42E-02	0.2974
**mmu-miR-29b-2-5p**	chr1	1.95E+08	1.95E+08	+	CUGGUUUCACAUGGUGGCUUAGAU	-0.27	529.49	580.86	478.12	0.11	-2.45	1.45E-02	0.2974
**mmu-miR-5108**	chr10	61774795	61774813	+	GUAGAGCACUGGAUGGUUU	0.80	15.18	7.73	22.63	0.33	2.44	1.46E-02	0.2974

Table S2. Differences in miRNA expression in the mPFC between vulnerable and resilient mice from the replica sample.

Mature	Chromosome	Start	Stop	Strand	Sequence	log2Fold Change	baseMean	baseMean _control	baseMean _treatment _files_1	lfcSE	Stat	Pvalue	Padj FDR
**mmu-miR-764-3p**	chrX	1.47E+08	1.47E+08	+	AGGAGGCCAUAGUGGCAACUGU	-0.79	10.51	14.83	6.19	0.23	-3.41	6.45E-04	0.196
**mmu-miR-322-5p**	chrX	53054306	53054327	-	CAGCAGCAAUUCAUGUUUUGGA	-0.47	102.13	120.23	84.04	0.14	-3.34	8.38E-04	0.196
**mmu-miR-223-3p**	chrX	96242884	96242905	+	UGUCAGUUUGUCAAAUACCCCA	-0.75	34.25	47.45	21.04	0.23	-3.29	1.01E-03	0.196
**mmu-miR-362-3p**	chrX	7241984	7242005	-	AACACACCUGUUCAAGGAUUCA	-0.54	133.06	161.52	104.61	0.16	-3.28	1.05E-03	0.196
**mmu-miR-3085-3p**	chr19	42280095	42280115	-	UCUGGCUGCUAUGGCCCCCUC	-0.55	239.47	292.99	185.95	0.18	-3.11	1.88E-03	0.272
**mmu-miR-30b-5p**	chr15	68337469	68337490	-	UGUAAACAUCCUACACUCAGCU	-0.47	5804.13	6869.27	4738.99	0.15	-3.05	2.32E-03	0.272
**mmu-miR-7a-1-3p**	chr13	58392800	58392821	-	CAACAAAUCACAGUCUGCCAUA	-0.49	181.71	216.30	147.12	0.16	-3.02	2.55E-03	0.272
**mmu-miR-532-5p**	chrX	7248456	7248477	-	CAUGCCUUGAGUGUAGGACCGU	0.29	5703.96	5097.28	6310.64	0.10	2.90	3.68E-03	0.273
**mmu-miR-15a-3p**	chr14	61632038	61632059	-	CAGGCCAUACUGUGCUGCCUCA	-0.56	30.54	37.80	23.27	0.20	-2.89	3.81E-03	0.273
**mmu-miR-3071-3p**	chr12	1.1E+08	1.1E+08	-	AUCAUCAAAACAAAUGGAGUCC	0.25	2216.12	2019.22	2413.01	0.09	2.88	3.92E-03	0.273
**mmu-miR-7015-3p**	chr4	1.21E+08	1.21E+08	-	UCUCACUGUCCUCUGCACUAG	0.42	70.41	59.18	81.64	0.15	2.88	4.03E-03	0.273
**mmu-miR-665-3p**	chr12	1.1E+08	1.1E+08	+	ACCAGGAGGCUGAGGUCCCU	-0.47	300.46	356.86	244.06	0.17	-2.82	4.88E-03	0.291
**mmu-miR-125b-1-3p**	chr9	41581980	41582001	+	ACGGGUUAGGCUCUUGGGAGCU	0.22	1128.14	1038.34	1217.95	0.08	2.77	5.69E-03	0.291
**mmu-miR-205-5p**	chr1	1.94E+08	1.94E+08	-	UCCUUCAUUCCACCGGAGUCUG	-0.58	24.60	31.41	17.79	0.21	-2.74	6.10E-03	0.291
**mmu-miR-324-3p**	chr11	70012095	70012114	+	CCACUGCCCCAGGUGCUGCU	-0.38	344.12	393.42	294.82	0.14	-2.74	6.18E-03	0.291
**mmu-miR-671-5p**	chr5	24592132	24592154	+	AGGAAGCCCUGGAGGGGCUGGAG	-0.40	155.59	179.79	131.40	0.15	-2.62	8.72E-03	0.291
**mmu-miR-1966-3p**	chr8	1.06E+08	1.06E+08	+	UUUCUGACUCAACUCUCCCUUAG	0.57	14.19	10.20	18.18	0.22	2.61	8.98E-03	0.291
**mmu-miR-369-3p**	chr12	1.1E+08	1.1E+08	+	AAUAAUACAUGGUUGAUCUUU	-0.37	9666.76	11044.99	8288.53	0.14	-2.61	9.10E-03	0.291
**mmu-miR-5617-3p**	chrX	20863126	20863146	-	CAGGCGGCCUCAGCUCUCACU	0.58	11.52	8.06	14.97	0.23	2.59	9.57E-03	0.291
**mmu-miR-425-5p**	chr9	1.09E+08	1.09E+08	+	AAUGACACGAUCACUCCCGUUGA	-0.19	765.14	815.25	715.02	0.07	-2.57	1.01E-02	0.291
**mmu-miR-339-5p**	chr5	1.39E+08	1.39E+08	-	UCCCUGUCCUCCAGGAGCUCACG	-0.35	891.08	1011.06	771.09	0.14	-2.55	1.07E-02	0.291
**mmu-miR-532-3p**	chrX	7248419	7248440	-	CCUCCCACACCCAAGGCUUGCA	-0.42	167.92	196.01	139.82	0.17	-2.55	1.09E-02	0.291
**mmu-miR-99b-3p**	chr17	17830232	17830253	+	CAAGCUCGUGUCUGUGGGUCCG	0.19	1148.51	1069.77	1227.25	0.08	2.55	1.09E-02	0.291
**mmu-miR-129-5p**	chr2	94241419	94241439	-	CUUUUUGCGGUCUGGGCUUGC	0.32	631.42	556.69	706.15	0.12	2.54	1.11E-02	0.291
**mmu-miR-15b-5p**	chr3	69009775	69009796	+	UAGCAGCACAUCAUGGUUUACA	-0.40	84.43	97.78	71.08	0.16	-2.54	1.12E-02	0.291
**mmu-miR-107-3p**	chr19	34820700	34820722	-	AGCAGCAUUGUACAGGGCUAUCA	-0.46	1533.63	1831.02	1236.25	0.18	-2.52	1.16E-02	0.291
**mmu-miR-212-5p**	chr11	75173403	75173425	+	ACCUUGGCUCUAGACUGCUUACU	0.31	10166.02	8986.87	11345.17	0.12	2.52	1.16E-02	0.291
**mmu-miR-29b-3p**	chr1	1.95E+08	1.95E+08	+	UAGCACCAUUUGAAAUCAGUGUU	-0.26	3314.85	3629.35	3000.35	0.10	-2.52	1.18E-02	0.291
**mmu-miR-673-3p**	chr12	1.1E+08	1.1E+08	+	UCCGGGGCUGAGUUCUGUGCACC	0.31	198.08	174.62	221.54	0.12	2.52	1.19E-02	0.291
**mmu-miR-301b-3p**	chr16	17124420	17124442	-	CAGUGCAAUGGUAUUGUCAAAGC	-0.54	12.64	15.93	9.36	0.22	-2.51	1.22E-02	0.291
**mmu-miR-344h-3p**	chr7	61742366	61742387	-	GGUAUAACCAAAGCCCGACUGU	-0.59	9.22	12.47	5.98	0.24	-2.50	1.25E-02	0.291
**mmu-miR-384-3p**	chrX	1.05E+08	1.05E+08	-	AUUCCUAGAAAUUGUUCACAAU	-0.26	2062.79	2257.06	1868.51	0.10	-2.50	1.25E-02	0.291

**mmu-miR-3552**	chrX	1.47E+08	1.47E+08	+	AGGCUGCAGGCCCACUUCCCU	-0.48	77.20	93.12	61.29	0.19	-2.48	1.30E-02	0.294
**mmu-miR-29c-3p**	chr1	1.95E+08	1.95E+08	+	UAGCACCAUUUGAAAUCGGUUA	-0.35	2060.52	2336.75	1784.29	0.14	-2.46	1.39E-02	0.303
**mmu-miR-350-3p**	chr1	1.77E+08	1.77E+08	-	UUCACAAAGCCCAUACACUUUC	-0.49	197.39	239.84	154.94	0.20	-2.45	1.42E-02	0.303
**mmu-miR-497a-5p**	chr11	70234730	70234751	+	CAGCAGCACACUGUGGUUUGUA	-0.34	550.41	621.76	479.05	0.14	-2.42	1.56E-02	0.317
**mmu-miR-30a-3p**	chr1	23272315	23272336	+	CUUUCAGUCGGAUGUUUGCAGC	0.23	6632.13	6077.69	7186.58	0.10	2.41	1.58E-02	0.317
**mmu-miR-487b-3p**	chr12	1.1E+08	1.1E+08	+	AAUCGUACAGGGUCAUCCACUU	-0.22	4314.35	4653.00	3975.70	0.09	-2.40	1.62E-02	0.317
**mmu-miR-296-5p**	chr2	1.74E+08	1.74E+08	-	AGGGCCCCCCCUCAAUCCUGU	-0.50	283.77	348.09	219.45	0.21	-2.40	1.66E-02	0.317
**mmu-miR-674-3p**	chr2	1.17E+08	1.17E+08	+	CACAGCUCCCAUCUCAGAACAA	0.39	4375.34	3694.44	5056.24	0.16	2.38	1.73E-02	0.317
**mmu-miR-324-5p**	chr11	70012060	70012082	+	CGCAUCCCCUAGGGCAUUGGUGU	-0.38	253.32	291.29	215.36	0.16	-2.38	1.74E-02	0.317
**mmu-miR-1198-5p**	chrX	7807142	7807163	+	UAUGUGUUCCUGGCUGGCUUGG	0.26	2621.91	2370.06	2873.76	0.11	2.37	1.79E-02	0.318
**mmu-miR-409-3p**	chr12	1.1E+08	1.1E+08	+	GAAUGUUGCUCGGUGAACCCCU	0.22	7198.65	6625.21	7772.10	0.09	2.35	1.85E-02	0.319
**mmu-miR-770-5p**	chr12	1.1E+08	1.1E+08	+	AGCACCACGUGUCUGGGCCACG	-0.38	1906.63	2189.69	1623.57	0.16	-2.35	1.88E-02	0.319
**mmu-miR-434-5p**	chr12	1.1E+08	1.1E+08	+	GCUCGACUCAUGGUUUGAACCA	0.19	27895.26	26053.65	29736.87	0.08	2.34	1.93E-02	0.320
**mmu-miR-5106**	chr4	44221197	44221219	-	AGGUCUGUAGCUCAGUUGGCAGA	0.46	299.82	105.13	494.52	0.20	2.33	2.01E-02	0.325
**mmu-miR-29a-3p**	chr6	31062673	31062694	-	UAGCACCAUCUGAAAUCGGUUA	-0.23	184508.74	199737.34	169280.13	0.10	-2.32	2.05E-02	0.325
**hsa-miR-4443**	chr3	48196572	48196588	+	UUGGAGGCGUGGGUUUU	0.52	1085.89	794.96	1376.81	0.23	2.29	2.19E-02	0.337
**mmu-miR-153-5p**	chr12	1.17E+08	1.17E+08	+	UUUGUGACGUUGCAGCU	0.20	334.92	311.10	358.73	0.09	2.28	2.24E-02	0.337
**mmu-miR-342-3p**	chr12	1.09E+08	1.09E+08	+	UCUCACACAGAAAUCGCACCCGU	-0.34	2793.95	3160.09	2427.81	0.15	-2.28	2.26E-02	0.337
**mmu-miR-874-3p**	chr13	58023135	58023156	-	CUGCCCUGGCCCGAGGGACCGA	-0.20	1956.34	2096.91	1815.77	0.09	-2.27	2.35E-02	0.343
**mmu-miR-154-3p**	chr12	1.1E+08	1.1E+08	+	AAUCAUACACGGUUGACCUAUU	-0.39	300.86	349.12	252.60	0.17	-2.26	2.39E-02	0.343
**mmu-miR-361-5p**	chrX	1.13E+08	1.13E+08	-	UUAUCAGAAUCUCCAGGGGUAC	-0.30	2549.36	2839.91	2258.81	0.13	-2.24	2.49E-02	0.343
**mmu-miR-1981-5p**	chr1	1.85E+08	1.85E+08	-	GUAAAGGCUGGGCUUAGACGUGGC	0.35	1404.92	1215.52	1594.33	0.15	2.24	2.49E-02	0.343
**mmu-miR-100-5p**	chr9	41531437	41531458	+	AACCCGUAGAUCCGAACUUGUG	0.29	59293.02	52784.51	65801.53	0.13	2.22	2.62E-02	0.343
**mmu-miR-1839-3p**	chr7	81529958	81529980	+	AGACCUACUUAUCUACCAACAGC	-0.34	179.94	203.57	156.30	0.15	-2.20	2.78E-02	0.343
**mmu-miR-679-5p**	chr12	1.1E+08	1.1E+08	+	GGACUGUGAGGUGACUCUUGGU	0.37	42.55	36.05	49.05	0.17	2.20	2.80E-02	0.343
**mmu-miR-192-5p**	chr19	6264857	6264877	+	CUGACCUAUGAAUUGACAGCC	0.25	2508.12	2282.71	2733.53	0.11	2.19	2.83E-02	0.343
**mmu-miR-103-3p**	chr2	1.31E+08	1.31E+08	+	AGCAGCAUUGUACAGGGCUAUGA	-0.33	16820.38	18998.17	14642.59	0.15	-2.18	2.95E-02	0.343
**mmu-miR-149-3p**	chr1	92850421	92850442	+	GAGGGAGGGACGGGGGCGGUGC	0.51	7.77	5.49	10.04	0.23	2.17	3.04E-02	0.343
**mmu-miR-190a-5p**	chr9	67236700	67236721	-	UGAUAUGUUUGAUAUAUUAGGU	-0.37	144.31	165.86	122.76	0.17	-2.16	3.05E-02	0.343
**mmu-miR-99b-5p**	chr17	17830194	17830215	+	CACCCGUAGAACCGACCUUGCG	0.21	27910.63	25793.70	30027.57	0.10	2.16	3.05E-02	0.343
**mmu-miR-27b-5p**	chr13	63300718	63300739	+	AGAGCUUAGCUGAUUGGUGAAC	0.27	115.23	103.83	126.63	0.13	2.16	3.09E-02	0.343
**mmu-miR-544-5p**	chr12	1.1E+08	1.1E+08	+	UCUUGUUAAAAAGCAGAGUCU	-0.42	38.91	46.05	31.76	0.19	-2.16	3.10E-02	0.343
**mmu-miR-383-5p**	chr8	38252178	38252199	-	AGAUCAGAAGGUGACUGUGGCU	0.18	11175.54	10461.11	11889.98	0.08	2.16	3.11E-02	0.343
**mmu-miR-30e-3p**	chr4	1.21E+08	1.21E+08	-	CUUUCAGUCGGAUGUUUACAGC	0.24	5572.33	5088.40	6056.26	0.11	2.15	3.12E-02	0.343
**mmu-miR-7224-3p**	chr2	67675496	67675516	+	UCCACUGAGAGGACCACCCAC	0.30	129.25	114.44	144.07	0.14	2.15	3.15E-02	0.343
**mmu-miR-137-3p**	chr3	1.18E+08	1.18E+08	+	UUAUUGCUUAAGAAUACGCGUAG	-0.36	7006.74	8018.38	5995.11	0.17	-2.15	3.15E-02	0.343

**mmu-miR-129-2-3p**	chr2	94241376	94241397	-	AAGCCCUUACCCCAAAAAGCAU	-0.43	4414.95	5263.70	3566.21	0.20	-2.14	3.20E-02	0.343
**mmu-miR-1964-3p**	chr7	29773344	29773365	+	CCGACUUCUGGGCUCCGGCUUU	0.39	119.49	100.33	138.64	0.18	2.14	3.23E-02	0.343
**mmu-miR-124-3p**	chr3	17795723	17795742	+	UAAGGCACGCGGUGAAUGCC	-0.22	77118.81	83266.03	70971.59	0.10	-2.14	3.26E-02	0.343
**mmu-miR-370-5p**	chr12	1.1E+08	1.1E+08	+	CAGGUCACGUCUCUGCAGUU	0.15	2789.59	2641.15	2938.04	0.07	2.13	3.35E-02	0.347
**mmu-miR-145a-5p**	chr18	61647866	61647888	-	GUCCAGUUUUCCCAGGAAUCCCU	-0.47	2541.55	3148.75	1934.35	0.22	-2.11	3.45E-02	0.347
**mmu-miR-3072-3p**	chr12	1.1E+08	1.1E+08	+	UGCCCCCUCCAGGAAGCCUUCU	-0.40	115.77	135.65	95.89	0.19	-2.11	3.47E-02	0.347
**mmu-miR-543-3p**	chr12	1.1E+08	1.1E+08	+	AAACAUUCGCGGUGCACUUCUU	0.26	13502.64	12216.54	14788.73	0.12	2.11	3.49E-02	0.347
**mmu-miR-544-3p**	chr12	1.1E+08	1.1E+08	+	AUUCUGCAUUUUUAGCAAGCUC	-0.40	45.85	53.65	38.05	0.19	-2.08	3.74E-02	0.348
**mmu-miR-211-5p**	chr7	64205831	64205852	+	UUCCCUUUGUCAUCCUUUGCCU	-0.49	67.45	93.08	41.83	0.24	-2.07	3.81E-02	0.348
**mmu-miR-541-5p**	chr12	1.1E+08	1.1E+08	+	AAGGGAUUCUGAUGUUGGUCACAC	0.22	29095.39	26750.92	31439.87	0.11	2.07	3.84E-02	0.348
**mmu-miR-146b-5p**	chr19	46342790	46342811	+	UGAGAACUGAAUUCCAUAGGCU	0.22	8780.42	8066.18	9494.66	0.11	2.07	3.85E-02	0.348
**mmu-miR-574-3p**	chr5	64970364	64970385	+	CACGCUCAUGCACACACCCACA	-0.37	128.61	148.10	109.13	0.18	-2.07	3.87E-02	0.348
**mmu-miR-501-3p**	chrX	7241270	7241291	-	AAUGCACCCGGGCAAGGAUUUG	0.21	1050.86	969.22	1132.50	0.10	2.06	3.91E-02	0.348
**mmu-miR-30d-5p**	chr15	68341257	68341278	-	UGUAAACAUCCCCGACUGGAAG	0.18	113979.10	106465.06	121493.14	0.09	2.06	3.91E-02	0.348
**mmu-miR-876-5p**	chr4	36645422	36645443	-	UGGAUUUCUCUGUGAAUCACUA	-0.44	15.09	18.05	12.12	0.21	-2.06	3.93E-02	0.348
**mmu-miR-7b-3p**	chr17	56243055	56243076	+	CAACAAGUCACAGCCAGCCUCA	-0.40	22.99	26.84	19.14	0.19	-2.06	3.94E-02	0.348
**mmu-miR-23a-3p**	chr8	84208563	84208583	+	AUCACAUUGCCAGGGAUUUCC	-0.28	4456.71	4932.33	3981.08	0.14	-2.05	4.00E-02	0.348
**mmu-miR-664-3p**	chr1	1.85E+08	1.85E+08	+	UAUUCAUUUACUCCCCAGCCUA	-0.31	1066.18	1196.30	936.05	0.15	-2.04	4.14E-02	0.348
**mmu-miR-122-5p**	chr18	65248866	65248887	+	UGGAGUGUGACAAUGGUGUUUG	0.29	114.01	101.51	126.51	0.14	2.04	4.16E-02	0.348
**mmu-miR-326-3p**	chr7	99552328	99552348	+	CCUCUGGGCCCUUCCUCCAGU	-0.32	1126.14	1268.71	983.58	0.16	-2.04	4.18E-02	0.348
**mmu-miR-6540-3p**	chr16	42303386	42303406	-	UCUGAAGCUUGCUUACCUCCA	-0.41	17.45	20.54	14.35	0.20	-2.03	4.21E-02	0.348
**mmu-let-7a-2-3p**	chr9	41536777	41536797	+	CUGUACAGCCUCCUAGCUUUC	-0.43	14.66	17.66	11.67	0.21	-2.03	4.22E-02	0.348
**mmu-miR-376b-3p**	chr12	1.1E+08	1.1E+08	+	AUCAUAGAGGAACAUCCACUU	-0.23	5684.38	6157.18	5211.59	0.11	-2.03	4.24E-02	0.348
**mmu-miR-194-5p**	chr19	6264658	6264679	+	UGUAACAGCAACUCCAUGUGGA	-0.25	1525.31	1666.48	1384.14	0.12	-2.02	4.36E-02	0.354
**mmu-miR-99a-5p**	chr16	77598940	77598961	+	AACCCGUAGAUCCGAUCUUGUG	0.23	168217.84	153641.40	182794.28	0.12	2.01	4.47E-02	0.359
**mmu-miR-126b-5p**	chr2	26591399	26591420	-	AUUAUUACUCACGGUACGAGUU	0.23	171.83	157.54	186.12	0.11	2.00	4.60E-02	0.361
**mmu-miR-344-5p**	chr7	61940073	61940098	-	AGUCAGGCUCCUGGCUAGAUUCCA	-0.47	5.40	7.16	3.64	0.24	-1.99	4.62E-02	0.361
**mmu-miR-154-5p**	chr12	1.1E+08	1.1E+08	+	UAGGUUAUCCGUGUUGCCUUCG	-0.26	1068.28	1174.09	962.46	0.13	-1.99	4.67E-02	0.361
**mmu-miR-325-5p**	chrX	1.05E+08	1.05E+08	-	CCUAGUAGGUGCUCAGUAAGUGU	0.20	1067.44	992.30	1142.58	0.10	1.99	4.69E-02	0.361
**mmu-miR-935**	chr7	3415120	3415143	+	CCCAGUUACCGCUUCCGCUACCGC	-0.30	338.73	378.39	299.07	0.15	-1.96	4.96E-02	0.365
**mmu-miR-195a-5p**	chr11	70235062	70235082	+	UAGCAGCACAGAAAUAUUGGC	-0.25	684.45	749.87	619.04	0.13	-1.96	4.98E-02	0.365
**mmu-miR-101a-5p**	chr4	1.01E+08	1.01E+08	-	UCAGUUAUCACAGUGCUGAUGC	-0.39	21.48	25.28	17.69	0.20	-1.96	4.99E-02	0.365

Table S3. RNA-seq p-values of the target genes of the three selected miRNAs.

Target Genes for miR-665-3p

Target Gene	RNAseq P-Value	
	Discovery	Replica
Iqcg	3.00E-04	0.7583
Slc2a4	5.90E-04	0.2333
Ercc2	8.90E-04	0.7306
Sgms2	1.70E-03	0.7464
Plin3	1.90E-03	0.8734
Ajuba	3.60E-03	0.0227
Trp53inp2	3.90E-03	0.0369
Pla2g16	3.90E-03	Np
Slc15a1	5.90E-03	0.8107
Frmd4b	8.80E-03	0.6809
Micall1	9.20E-03	0.7463
Zbtb7b	9.40E-03	0.4818
Elovl1	0.0102	0.1193
Irgm2	0.011	0.8259
Mroh1	0.0116	0.0596
Tapbp	0.0126	0.0259
Mzt1	0.0126	0.71
Decr2	0.0129	0.4821
Pou2f2	0.014	0.0916
Arhgap28	0.0141	0.9098
Nnat	0.0145	0.0138
Vamp3	0.0149	0.9968

Target genes for miR-29c-3p

Target Gene	RNAseq P-Value	
	Discovery	Replica
Mfap2	8.70E-05	0.8638
Ak3	2.40E-04	0.4251
Zfp36	1.90E-03	0.2193
Prelp	3.00E-03	0.3326
Trp53inp2	3.90E-03	0.0369
Nasp	4.50E-03	0.4786
Ldlrap1	5.00E-03	0.9162
Arrdc3	5.20E-03	0.0682
Pmp22	5.50E-03	0.8493
Lamc1	7.40E-03	0.782
Col3a1	7.70E-03	Np
Trp53inp1	8.00E-03	0.0971
Bmpr1a	8.00E-03	0.7643
Col4a5	8.10E-03	0.0684
Hspg2	8.10E-03	0.1123
Pcdha11	8.30E-03	0.0196
Map4k4	8.40E-03	0.4935
Nckap5l	8.70E-03	0.1517
Ss18l1	9.70E-03	0.8385
Slc38a2	0.0113	0.9096
Nfatc4	0.0121	0.0751
Mlf1	0.0128	0.8472

Target genes for miR-137-3p

Target Gene	RNAseq P-Value	
	Discovery	Replica
March9	3.01E-04	0.5005
Flnc	8.08E-04	0.3575
Prkd3	1.22E-03	0.7184
Igfbp5	1.91E-03	0.2298
Neurod1	2.65E-03	0.4825
Ankrd13a	2.76E-03	0.4012
March8	4.02E-03	0.3812
Tox3	4.24E-03	3.03E-03
Fgl2	6.22E-03	Np
Epha7	6.46E-03	0.2595
Npc1	6.51E-03	0.3037
Ptp4a3	6.86E-03	0.1256
Capn2	6.91E-03	0.9968
Kat2b	7.17E-03	0.7636
Itga9	7.53E-03	0.3914
Fgf7	7.85E-03	0.4546
Tead1	8.30E-03	0.91
Mtus1	8.45E-03	0.3436
Otud7b	8.78E-03	0.5738
Ezh2	9.32E-03	0.1636
Zbtb7b	9.45E-03	0.4818
Svil	9.79E-03	0.2551

Ankrd34a	0.0154	0.4814	*Col5a2*	0.0133	0.038	*Elovl1*	0.0102	0.1193
Fam19a1	0.0161	Np	*Gab1*	0.0136	0.3259	*Ssx2ip*	0.0108	0.234
Acot11	0.0169	0.9658	*Pou2f2*	0.014	0.0916	*Fmnl2*	0.0121	0.9881
Gpcpd1	0.0171	0.97	*Arhgap28*	0.0141	0.9098	*Efna3*	0.0121	0.6134
Adam11	0.0185	0.7286	*Daam2*	0.0147	0.2505	*Ss18*	0.0124	0.5224
Hist1h4i	0.0193	Np	*Stag2*	0.0148	0.2811	*Osbp2*	0.013	0.1093
Gla	0.0198	0.3882	*Vamp3*	0.0149	0.9968	*Serpinf1*	0.0131	0.1735
Gdpgp1	0.0201	0.466	*Sgk1*	0.0149	0.5782	*Dlgap1*	0.0131	0.52
Ston2	0.0219	0.554	*Il1rap*	0.0155	0.1841	*Gramd3*	0.0135	0.1148
Lpp	0.0222	0.0634	*Gng12*	0.016	0.7079	*Specc1l*	0.0144	0.513
Adamts1	0.0223	0.55	*Col4a6*	0.0162	0.0905	*Slc16a9*	0.0146	0.1477
Prdx1	0.0224	0.3332	*Qk*	0.0168	0.8641	*Zmynd11*	0.0147	0.4705
Smtnl2	0.0227	0.9565	*Calu*	0.017	0.451	*Sgk1*	0.0149	0.5782
Rhog	0.0234	0.5905	*Gpcpd1*	0.0171	0.97	*D16ertd472e*	0.0153	0.2719
Abhd4	0.0244	0.8491	*Sh3glb1*	0.0173	0.5456	*Fat3*	0.0156	0.2931
Plekhh2	0.0254	0.534	*Enpp2*	0.0173	0.0324	*Qk*	0.0168	0.8641
Sestd1	0.0255	0.95	*Sms*	0.0178	0.3724	*Pak2*	0.0169	0.5903
R3hdm1	0.028	0.6007	*Itgb1*	0.0183	0.3483	*Cttnbp2nl*	0.017	0.5486
Agap2	0.0287	0.2054	*Frem1*	0.0187	0.2853	*Gpcpd1*	0.0171	0.97
Pigv	0.0292	0.5691	*Sp1*	0.019	0.9186	*Gltp*	0.0171	0.4387
Nxt2	0.0298	0.9802	*Slc30a3*	0.0192	0.8591	*Aldh1a2*	0.0173	Np
Dock3	0.0311	0.9669	*Cdk6*	0.0197	0.6646	*Cdk6*	0.0197	0.6646
Abi2	0.0324	0.3676	*Col19a1*	0.0198	0.7261	*Lbh*	0.0197	0.9106
Tnfaip6	0.033	0.6581	*Col1a2*	0.0204	0.1134	*Col19a1*	0.0198	0.7261
Arpc4	0.0334	0.3871	*Smtnl2*	0.0227	0.9565	*Kcnd1*	0.0201	0.2584
Magi1	0.0336	0.7567	*Xkr4*	0.0232	0.4042	*Plcb1*	0.0216	0.0142

Chrm1	0.0352	0.4402	*Fbxw9*	0.0235	0.2259	*Slc12a2*	0.0249	0.6439
Bcl7a	0.0353	0.9331	*Elf2*	0.0236	0.625	*Fstl1*	0.025	7.45E-03
Kcnk6	0.0356	0.4593	*Emp1*	0.0238	0.1846	*Trim3*	0.0251	0.4046
Cep97	0.0376	0.2125	*Ptpn11*	0.024	0.5829	*Ugcg*	0.0273	0.2365
Tor1aip2	0.0384	0.9228	*Abhd4*	0.0244	0.8491	*Mgat5b*	0.0277	0.5991
Sufu	0.0386	0.2894	*Nfatc3*	0.0249	0.643	*Gls*	0.0291	0.2767
Kl	0.0386	0.0537	*Serpinh1*	0.0249	0.7587	*Abcg4*	0.0294	0.6244
Thap2	0.0409	0.7109	*Fstl1*	0.025	0.0075	*Zfpm2*	0.0296	0.2926
Atad2	0.0411	0.0249	*Sestd1*	0.0255	0.95	*Sox11*	0.0296	0.7993
Trim44	0.0447	0.4883	*Mybl2*	0.0256	0.2905	*Nxt2*	0.0298	0.9802
Rmi2	0.0449	0.594	*Pcdhac2*	0.0262	0.2981	*Oxsr1*	0.0301	0.698
Ranbp3l	0.0456	0.1552	*Hepacam*	0.0266	0.9029	*Zdhhc1*	0.0304	0.5474
Vezf1	0.0469	0.4848	*Slc22a5*	0.0266	0.8398	*Usp28*	0.0306	0.3771
Nrgn	0.0474	0.1729	*Dcaf7*	0.0268	0.3641	*Erbb2ip*	0.0308	Np
Fndc3b	0.0486	0.5035	*Oxtr*	0.0274	0.0268	*Dnlz*	0.0309	0.945
Actl6a	0.0491	0.7466	*Pcdha1*	0.0287	0.5914	*Gulp1*	0.0348	0.3915
Dnase2a	0.0502	0.1149	*Zfp652*	0.0287	0.552	*Robo2*	0.035	0.0711
Slc23a2	0.0535	0.9043	*Ltbr*	0.0294	0.2447	*Pik3r3*	0.0352	0.3098
Scn8a	0.0554	0.0506	*Ncoa4*	0.0294	0.4685	*Fbxl7*	0.0355	0.2769
Tmem201	0.0568	0.955	*Nid2*	0.0295	0.317	*Kcnk6*	0.0356	0.4593
Rbfox1	0.0575	0.1516	*Fermt2*	0.0323	0.8667	*Ikbke*	0.0373	0.5293
9930104l06rik	0.0633	0.9247	*Tnfrsf1a*	0.0327	0.2099	*Cacna1d*	0.0376	0.723
Slc17a7	0.0646	0.956	*Kifc2*	0.0336	0.1395	*Slc45a3*	0.0387	0.8313
Plekhh1	0.065	0.4037	*Adam12*	0.0337	0.0389	*Tspan2*	0.0392	0.5085
Mtf1	0.0653	0.0012	*Pik3r3*	0.0352	0.3098	*Rufy2*	0.0409	0.8328
Kif21b	0.0661	0.5011	*Fign*	0.0353	0.3021	*Slc6a9*	0.0411	0.3921

Nrcam	0.0668	0.3154	*Bcl7a*	0.0353	0.9331	*Tsnax*	0.0416	0.2772
Dmwd	0.0722	0.0459	*Iffo1*	0.0361	0.4469	*Zdhhc21*	0.0431	0.2472
Tbl1xr1	0.0729	0.9606	*Ptp4a1*	0.037	0.0072	*Hic1*	0.0432	0.6354
Exosc4	0.0732	0.1275	*Adamts7*	0.0378	0.9537	*Jag1*	0.0433	0.0923
Lrrn4cl	0.0742	0.148	*Unc13b*	0.0378	0.8802	*Rcor2*	0.0452	0.4361
Ecm2	0.075	0.2213	*Bmf*	0.0403	0.5906	*Ryr2*	0.0453	0.5004
Traf4	0.0761	0.2057	*Hbp1*	0.041	0.7426	*Bhlhe22*	0.0469	0.2305
Dpysl5	0.0765	0.4591	*Zbtb5*	0.0413	0.93	*Bcl11a*	0.0485	0.9195
Tbc1d24	0.0818	0.7809	*Vcl*	0.0413	0.9913	*Yipf6*	0.0487	0.0702
Pclaf	0.0833	0.6576	*Zfp36l1*	0.0426	0.2418	*Myo1d*	0.0501	0.5662
Trim36	0.0835	0.4175	*Zdhhc21*	0.0431	0.2472	*Kcnc1*	0.0516	0.0433
Trim27	0.0845	0.8799	*Kirrel*	0.0439	0.0645	*Nat8l*	0.0523	0.3822
Ptprf	0.0878	0.7381	*Col8a1*	0.0445	0.252	*Nsf*	0.0539	0.2056
Fzd5	0.0903	0.563	*Rnf169*	0.0451	0.8297	*Megf9*	0.0544	0.528
Bcam	0.0918	0.1571	*Ndrg4*	0.0457	0.8237	*Syt1*	0.0554	0.0414
Efr3b	0.0928	0.3639	*Csgalnact2*	0.0475	0.8264	*Kdm1a*	0.0555	0.1499
Ext1	0.0934	0.1178	*Adamts9*	0.0486	0.0434	*Tmpo*	0.0557	0.8875
Kcnma1	0.0973	0.6124	*Kif26a*	0.049	0.5421	*Adam23*	0.057	0.1069
Mark2	0.0993	0.3195	*Slc39a9*	0.0494	0.0884	*Zfp523*	0.0594	0.1888
Rragd	0.1045	0.9956	*Tspan9*	0.051	0.2557	*Setd7*	0.0598	4.24E-03
Cacnb3	0.1081	0.9207	*Tmem183a*	0.0521	0.9314	*Car7*	0.0606	0.5971
Lrrc58	0.1087	0.8778	*Dynlt1b*	0.0529	0.7828	*Alpk3*	0.061	0.5872
Dtna	0.1126	0.5032	*Plp1*	0.0531	0.3612	*Slc43a2*	0.062	0.0537
Ncan	0.1207	0.9851	*Rnf165*	0.0533	0.9267	*Creb5*	0.0623	0.9746
Smarcd2	0.1244	0.2662	*Col24a1*	0.0539	0.0514	*Pappa*	0.0632	0.6818
Grin2b	0.1247	0.5974	*Reps2*	0.0543	0.0439	*Itga5*	0.0636	0.9431

Lix1	0.1248	0.4318
Bpnt1	0.1263	0.0914
Tm9sf4	0.1271	0.2425
Pde4c	0.129	0.1889
Nucks1	0.1348	0.757
sep-03	0.136	Np
Ccnt2	0.1388	0.5125
Impdh1	0.1416	0.1041
Fadd	0.1449	0.4523
Htr5a	0.146	0.115
Xrcc2	0.1497	0.8893
Afap1	0.1535	0.7642
Gtf2a1	0.1549	0.2438
Ube2n	0.1554	0.5646
Cdk16	0.1567	0.7533
Ubac1	0.1568	0.1478
Atxn7l3	0.1667	0.8897
Mrpl11	0.1671	0.2533
Fam96a	0.1691	Np
Ccdc127	0.1715	0.1141
Suds3	0.1719	0.5298
Aak1	0.1733	0.1542
Pnpla3	0.1771	0.2597
Soat1	0.1776	0.3751
Sco1	0.1792	0.7423
Gdap1l1	0.1822	0.4926

Slc16a1	0.0553	0.0105
Tmem201	0.0568	0.955
Mblac2	0.0569	0.3271
Pcdha4	0.0596	0.1386
Sox12	0.0598	0.0213
Ccdc117	0.0604	0.0514
Sparc	0.0608	0.889
Dbt	0.0612	0.8146
Dgkd	0.0616	0.5337
Slc43a2	0.062	0.0537
Creb5	0.0623	0.9746
Rnd3	0.0623	0.7731
Tsc22d3	0.0644	0.9509
Zfp346	0.0684	0.0104
Mmp2	0.0686	0.0839
Snx22	0.0686	0.3546
Fras1	0.0689	0.8767
Gtpbp2	0.069	0.7044
Fam136a	0.0703	0.3821
Arrdc4	0.0711	0.2302
Zfp827	0.0718	0.9112
Nfia	0.0731	0.0383
Calm3	0.0748	0.503
Stard8	0.0753	0.9081
Traf4	0.0761	0.2057
Dpysl5	0.0765	0.4591

Trp63	0.0645	0.6221
Sptlc1	0.0648	0.6839
Dusp4	0.065	0.0236
Ugp2	0.0658	0.8004
Zfp346	0.0684	0.0104
Thpo	0.0716	0.1863
Ppp5c	0.0743	0.5611
Asph	0.0755	0.681
Csmd1	0.076	0.9057
Tiparp	0.0764	0.6169
Cadps	0.0779	0.231
Foxc1	0.0783	0.0625
Ybx1	0.0799	0.6323
Lrp12	0.0804	0.573
Mitf	0.0804	0.0853
Plk2	0.0815	0.7518
Ephb3	0.0823	0.119
Rcan2	0.0831	0.2694
Glrb	0.0839	0.0316
Rell2	0.0845	0.9035
Rnf138	0.0868	0.8273
Prr16	0.0871	0.3995
Rell1	0.089	0.1415
Tle4	0.0897	0.3675
Slc6a8	0.0904	0.2601
Gca	0.0906	0.3021

Lats1	0.1829	0.2296	*Acap3*	0.077	0.8494	*Nck1*	0.093	0.1469
Onecut2	0.1831	0.3022	*Hmgcr*	0.0779	0.1173	*Sez6l2*	0.095	0.0247
Zfp113	0.1857	0.7817	*Eln*	0.0794	0.0394	*Ccny*	0.0962	0.603
Mmp24	0.1887	0.8482	*Nav3*	0.0795	0.6485	*Dusp8*	0.0969	0.7249
Iba57	0.1907	0.3749	*Ephb3*	0.0823	0.119	*B3galt5*	0.098	0.8936
Six4	0.1929	0.0878	*Col4a4*	0.0825	0.7957	*Mtpn*	0.0986	0.0537
Strbp	0.1931	0.2558	*Stx1a*	0.0834	0.3966	*Dclk1*	0.099	0.6141
Mrpl40	0.1943	0.5669	*Nup160*	0.0863	0.681	*Slc4a7*	0.1016	0.1504
Pin1	0.1965	0.8955	*Rnf138*	0.0868	0.8273	*Adcy2*	0.1024	0.1448
Nlgn3	0.1969	0.979	*Adamts18*	0.087	0.7013	*Mphosph9*	0.1028	0.4068
Git2	0.2005	0.401	*Pdgfrb*	0.0872	0.3353	*Kcnc3*	0.1033	0.0729
Bysl	0.2104	0.5014	*Dab1*	0.088	0.3701	*Cdc37l1*	0.1037	0.3191
Nrf1	0.2112	0.8185	*Fzd5*	0.0903	0.563	*Mapk10*	0.1041	0.0442
Cramp1l	0.2115	0.9219	*Paip2*	0.0904	0.9184	*Rragd*	0.1045	0.9956
Arhgap19	0.2183	0.1867	*Strn4*	0.0921	0.9294	*Ankrd44*	0.1061	0.406
Slc39a1	0.2199	0.7509	*Ldlrad3*	0.0922	0.6263	*Zfp217*	0.1065	0.3871
Cbfa2t2	0.2255	0.9224	*Cnr1*	0.093	0.3426	*Hk3*	0.1073	0.3824
Synpo	0.2299	0.276	*Rapgefl1*	0.0931	0.7997	*Ap3s1*	0.1137	0.8524
Fam208a	0.2307	Np	*Ppp1r3d*	0.0939	0.4598	*Tjp1*	0.1162	0.2865
Ercc6	0.2321	0.7811	*Lims1*	0.0941	0.5233	*Stx1b*	0.1214	0.0506
Mapk14	0.234	0.0868	*Trafd1*	0.0947	0.5654	*Serp1*	0.1241	0.9812
Bnip3l	0.2399	0.1451	*Itga11*	0.0947	0.7032	*Acvr1*	0.1249	0.2006
Sc5d	0.2471	0.0047	*Frmd5*	0.0949	0.3022	*Eaf1*	0.1249	0.029
Akt3	0.2516	0.6734	*Atrn*	0.0959	0.109	*Fkbp4*	0.1274	0.1343
Chd3	0.2546	0.0308	*Plekha1*	0.0969	0.7907	*Arl15*	0.1277	0.286
Scamp5	0.256	0.7493	*Ednrb*	0.0994	0.4466	*Elovl2*	0.1317	0.1786

Limch1	0.2617	0.7444	*Prr5l*	0.0998	0.7357	*Kcnab3*	0.1322	0.7022
Arvcf	0.2629	0.5248	*Slc25a22*	0.1001	0.6866	*Ptbp1*	0.1327	0.3406
Tmem55b	0.2641	Np	*Ppic*	0.1003	0.0914	*Nucks1*	0.1348	0.757
Steap4	0.2661	0.8457	*Zhx1*	0.1005	0.337	*sep-03*	0.136	Np
Tgfb2	0.2694	0.7256	*Dcun1d4*	0.1017	0.9605	*Pxn*	0.1378	0.3933
Zfp558	0.2696	0.4602	*Ubtd2*	0.1017	0.2187	*Scn1b*	0.1382	0.3875
Pdxk	0.2715	0.9348	*Gpr37*	0.1032	0.6088	*St18*	0.1394	0.6059
Tubb5	0.275	0.7121	*Kcnc3*	0.1033	0.0729	*Rbms3*	0.1404	0.5519
Eno2	0.277	0.6643	*Mapk10*	0.1041	0.0442	*Cacna1c*	0.1447	0.5042
Eif4a2	0.2773	0.6152	*Megf6*	0.1046	0.0325	*Dstyk*	0.1466	0.2599
Amot	0.2797	0.8563	*Pcyt1b*	0.1052	0.6311	*Kank4*	0.1475	0.2734
Hnrnpc	0.2824	0.8085	*Cep68*	0.1077	0.6027	*Src*	0.1495	0.9781
Luzp1	0.2874	0.0072	*Nrsn1*	0.109	0.2965	*Gorasp2*	0.1519	0.8094
Eid1	0.2878	0.2102	*Kbtbd8*	0.1097	0.871	*Rbm27*	0.152	0.3149
Zfp385c	0.2953	0.1719	*E2f7*	0.1116	0.4889	*Stt3b*	0.1527	0.1215
Ptpn2	0.2978	0.9265	*Pknox2*	0.1121	0.3297	*Ankrd12*	0.1529	0.1419
Dach1	0.3011	0.3804	*Rab6b*	0.1133	0.2357	*Slc12a6*	0.1549	0.1019
Mapre2	0.3057	0.0537	*Tubb2a*	0.1137	0.2047	*Rnf38*	0.1566	2.42E-03
Ifngr2	0.3085	0.9278	*Pdgfc*	0.1138	0.5147	*Slc6a15*	0.1571	0.2054
Frs2	0.3105	0.8557	*Mafb*	0.1165	0.8328	*Rps13*	0.1613	5.54E-03
Pou3f1	0.3121	0.5722	*Adamts16*	0.1174	0.1384	*Nckap5*	0.162	0.1628
Nlk	0.3127	0.0066	*Rsad1*	0.1195	0.4862	*Neurod2*	0.1628	0.9197
Nhsl2	0.3129	0.6094	*Clmn*	0.1224	0.4664	*Fam172a*	0.1634	0.6082
Bbc3	0.3156	0.2457	*Elmo2*	0.1228	0.5446	*Dag1*	0.1637	0.8578
Mkl2	0.3164	Np	*Hpcal4*	0.1238	0.2251	*Bach2*	0.166	0.3758
Kdm5c	0.3192	0.077	*Abcb6*	0.1242	0.5889	*Nbea*	0.1667	0.456

Mlst8	0.323	0.2887	*Stmn2*	0.1294	0.5859	*Ube3c*	0.1683	0.2626
Snx13	0.3242	0.0687	*Lin7a*	0.1307	0.6385	*Rbm24*	0.1695	0.5715
Lekr1	0.3246	0.8438	*Col4a3*	0.1354	0.0456	*Kcna2*	0.17	0.0135
Atrx	0.3272	0.1408	*Grip1*	0.1375	0.2173	*Aak1*	0.1733	0.1542
Tceanc	0.3353	0.8222	*Ccnt2*	0.1388	0.5125	*Mpp1*	0.1773	0.1385
Paxip1	0.3376	0.1626	*Impdh1*	0.1416	0.1041	*Akap2*	0.1784	Np
Gnl3l	0.344	0.481	*Pigt*	0.1439	0.3138	*Plk4*	0.1813	0.323
Capn5	0.3465	0.4212	*Tmod3*	0.1462	0.9446	*Onecut2*	0.1831	0.3022
Kdr	0.3511	0.4476	*Zfp282*	0.1463	0.6939	*Vash2*	0.1843	0.7723
Xbp1	0.3512	0.0805	*Bace1*	0.1482	0.279	*Atpaf1*	0.185	0.9849
Sema4c	0.3557	0.2929	*Src*	0.1495	0.9781	*Naa15*	0.1855	2.29E-03
Rbak	0.3578	0.847	*Dnm3*	0.1505	0.3293	*Eml5*	0.191	0.2241
Ostf1	0.3581	0.2256	*Trim37*	0.1511	0.0272	*Sgpl1*	0.1927	0.2021
Rfxank	0.3619	0.1388	*Abi1*	0.1518	0.305	*Slc46a3*	0.1927	0.7116
Agps	0.365	0.525	*Ccnd2*	0.1543	0.0465	*Oxr1*	0.1943	0.0481
Ing5	0.3662	0.6741	*F11r*	0.1549	0.1018	*Cdh12*	0.1951	0.0598
Hivep2	0.3669	0.106	*Eps15*	0.1559	0.1512	*Ythdc1*	0.1956	0.1149
Gk5	0.3755	0.645	*Pitpnm3*	0.1568	0.8142	*Arid4b*	0.1965	0.0745
Dars2	0.3784	0.6566	*Nckap5*	0.162	0.1628	*Vars*	0.197	0.4571
Emc1	0.3803	0.1405	*Nav1*	0.1622	0.662	*Tmem33*	0.1971	1.22E-03
Psd2	0.3837	0.9497	*Pcdhac1*	0.1622	0.6743	*Pitpna*	0.1975	0.215
Ccl9	0.384	0.1671	*Serinc5*	0.1632	0.6364	*Prkab1*	0.2001	0.4389
Hnrnpab	0.3903	0.859	*Pcdha7*	0.1634	0.396	*Cacna1i*	0.2003	0.0365
Ttc5	0.3914	0.4984	*Dag1*	0.1637	0.8578	*Rbpj*	0.2008	0.102
Zfp874b	0.393	0.1839	*Rab30*	0.1658	0.3435	*Mboat2*	0.2041	0.0428
Tmx4	0.3944	0.9149	*Bach2*	0.166	0.3758	*Aebp2*	0.2078	0.1815

Actr3	0.3981	0.889	*Kdm6b*	0.1709	0.9926	*Ctdsp2*	0.2098	0.8078
Ncl	0.3999	0.7808	*Slc16a2*	0.173	0.7024	*Dclk2*	0.2103	0.0286
Cacna1g	0.402	0.37	*Tmem65*	0.1755	0.097	*Rsbn1*	0.2145	0.1176
Zcchc2	0.4052	0.0009	*Shroom2*	0.1759	0.3737	*Gabra1*	0.2166	0.0371
Gatsl2	0.4084	Np	*Col6a3*	0.176	0.7816	*Atp1b1*	0.2171	0.5436
Srcin1	0.4096	0.6667	*Dnmt3b*	0.1762	0.4148	*Necab3*	0.2179	0.6661
Lasp1	0.4191	0.5481	*Cmpk1*	0.1765	0.9562	*Zfp36l2*	0.219	0.6706
Zbtb34	0.4195	0.2349	*Lysmd1*	0.1774	0.8386	*Lmtk2*	0.22	3.22E-03
Zfp459	0.4197	0.324	*Ric8*	0.1784	Np	*Trmt6*	0.2203	0.7231
Ing3	0.4225	0.0059	*Ttc14*	0.1784	0.3735	*Mtdh*	0.2215	0.9709
Zfp629	0.4248	0.7865	*Cbx2*	0.1818	0.5538	*Map3k14*	0.2232	0.37
Ccdc62	0.4272	0.2038	*Asb11*	0.1828	0.5056	*Dhx40*	0.2233	0.7992
Kcmf1	0.4311	0.3424	*Onecut2*	0.1831	0.3022	*Mef2a*	0.2248	0.7416
Stx17	0.4313	0.3294	*Pik3r1*	0.1831	0.1707	*Ctbp1*	0.2265	0.3418
Xpo1	0.4358	0.2263	*Gxylt2*	0.1839	0.0984	*Lemd3*	0.2266	0.1179
Trappc13	0.4363	0.0515	*Ccdc28b*	0.1841	0.3056	*Tspan33*	0.2305	0.4231
Nup214	0.438	0.7705	*Vash2*	0.1843	0.7723	*Syt9*	0.2306	0.5073
Bbs1	0.4414	0.9449	*Kcnc2*	0.1848	0.3639	*Hip1r*	0.2309	0.7054
Nop9	0.4448	0.5968	*Canx*	0.1851	0.0656	*Tbc1d19*	0.2309	0.177
Cd34	0.4448	0.112	*Camk1d*	0.1862	0.3861	*Rwdd4a*	0.2338	0.1345
Sap18	0.4451	0.567	*Dpp4*	0.1884	0.5902	*Lgi3*	0.2341	0.1059
Nkiras1	0.4458	0.2605	*Mmp24*	0.1887	0.8482	*Inpp5a*	0.2387	0.4842
Ppp1r9a	0.4498	0.1313	*Eml5*	0.191	0.2241	*Bnip3l*	0.2399	0.1451
Mga	0.4529	0.1735	*Erlin2*	0.192	0.3565	*Scn1a*	0.2417	0.0155
Cst6	0.4547	0.058	*Elovl4*	0.1925	0.196	*Bsn*	0.2428	0.31
Slc20a1	0.4619	0.017	*Vash1*	0.1927	0.6959	*Vcpip1*	0.2445	0.0183

Gaa	0.465	0.9947	*Dusp22*	0.1928	0.2826	*Zfp148*	0.2446	0.4569
Mlec	0.4668	0.2931	*Oxr1*	0.1943	0.0481	*Nacc2*	0.2454	0.5379
Hdac8	0.474	0.9331	*Spry4*	0.195	0.2089	*Chordc1*	0.2474	0.0231
Ap1g1	0.4764	0.0025	*Nlgn3*	0.1969	0.979	*Phf20*	0.2477	0.7242
Zcchc17	0.4771	0.568	*Setd5*	0.1969	0.9423	*Ahcyl2*	0.2504	0.5982
Ppp2r2a	0.4937	0.2609	*Nktr*	0.197	0.532	*Pip4k2c*	0.2505	0.0763
Ulk1	0.4938	0.0859	*Pitpna*	0.1975	0.215	*Gde1*	0.253	0.5553
Pycr1	0.497	0.3416	*Fbn1*	0.1978	0.0821	*Sipa1l2*	0.259	9.68E-03
Slc8b1	0.4995	0.1617	*Senp1*	0.198	0.8287	*Limch1*	0.2617	0.7444
Phf13	0.5038	0.1917	*Atp6v1a*	0.1992	0.4089	*Fnip1*	0.2625	0.0619
Slc4a4	0.505	0.3789	*Cx3cl1*	0.2066	0.107	*Max*	0.2627	0.5087
Tex9	0.5052	0.4685	*Col22a1*	0.2113	0.136	*Ints2*	0.2649	0.1634
Ing2	0.5101	0.1932	*Tdg*	0.2139	0.5728	*Grb7*	0.2658	0.1271
Zfp354a	0.5179	0.5553	*Maz*	0.2164	0.2486	*Man2a1*	0.267	1.36E-04
Usp45	0.5206	0.1245	*Atp1b1*	0.2171	0.5436	*Prdm1*	0.2671	0.2198
Slc27a1	0.5254	0.4195	*Gpr85*	0.2183	0.9753	*Cep55*	0.2673	0.437
Tstd2	0.5255	0.8239	*Pcdha3*	0.2183	0.2448	*Tmtc4*	0.2677	0.1232
Srf	0.528	0.551	*Cd276*	0.2199	0.0863	*Rgs7bp*	0.2691	0.0133
Rrp1b	0.5299	0.312	*Lmtk2*	0.22	0.0032	*Srgap3*	0.2704	0.2683
Efnb1	0.5322	0.7797	*Hexa*	0.2217	0.5771	*Tmem229b*	0.2712	0.5418
Serf2	0.5328	0.9061	*Usp42*	0.2249	0.6088	*Srsf1*	0.2782	0.2339
Aplp2	0.5332	0.0217	*Med26*	0.225	0.2526	*Plekho2*	0.2784	0.9355
Dhx37	0.539	0.5301	*Rybp*	0.2257	0.1732	*Ptprd*	0.2817	0.0775
Cops7b	0.5444	0.3668	*Agtr2*	0.2261	0.7046	*Phtf2*	0.2846	2.25E-03
Tox4	0.5449	0.395	*Zfp641*	0.2262	0.4407	*Appl2*	0.2871	0.2105
Knop1	0.5464	0.3036	*Lemd3*	0.2266	0.1179	*Luzp1*	0.2874	7.22E-03

Zbtb6	0.5483	0.9267	*Ankrd13b*	0.2275	0.7064	*Pitpnm2*	0.2875	0.6314
Eri2	0.553	0.2739	*Lactb2*	0.2278	0.5889	*Api5*	0.2879	0.1179
Scrt1	0.5566	0.0536	*Syt9*	0.2306	0.5073	*Sh3pxd2b*	0.291	0.6029
9130011e15rik	0.5698	Np	*Ercc6*	0.2321	0.7811	*Zc3h11a*	0.2934	0.283
Gtpbp3	0.5825	0.7047	*Ppp2ca*	0.235	0.4042	*Ncoa3*	0.2957	0.4109
Atxn1l	0.5894	0.2692	*Camk2g*	0.237	0.1363	*Ptpn2*	0.2978	0.9265
Osbpl8	0.5899	0.0466	*Dpysl2*	0.2373	0.1038	*Spty2d1*	0.2998	0.0436
Timmdc1	0.5902	0.7772	*Aff4*	0.2389	0.2591	*St3gal3*	0.3024	0.0867
Tmem263	0.5939	0.2438	*Mat2a*	0.2462	0.9118	*8030462n17rik*	0.3059	0.1415
Xpo7	0.5968	0.0584	*Akt3*	0.2516	0.6734	*Pdlim3*	0.3067	0.1875
Commd8	0.5998	0.5154	*Rcor1*	0.2536	0.2406	*Cxcl12*	0.3115	0.7447
Ifnar1	0.6041	0.9411	*Cyb561d1*	0.2547	0.3926	*Kpnb1*	0.313	0.2613
Ddhd2	0.6058	0.6303	*Arhgap5*	0.2551	0.7138	*Erg*	0.3132	0.2569
Plxna2	0.6091	0.8531	*Daam1*	0.2552	0.0119	*Ube2g2*	0.3157	0.4271
Srxn1	0.6124	0.6038	*Efna5*	0.2558	0.0459	*Sos1*	0.3174	0.0178
Pex13	0.6126	0.0471	*Scamp5*	0.256	0.7493	*Tmem55a*	0.3207	Np
Pctp	0.6127	0.8126	*Arpp19*	0.2569	0.021	*Plekha5*	0.322	0.9284
Cercam	0.6148	0.2327	*Pcdha2*	0.257	0.9926	*Xpo4*	0.3255	0.8781
Ankrd13c	0.6246	0.8007	*Fbn2*	0.2591	0.8017	*Srpr*	0.3269	0.4632
Tube1	0.6267	0.6358	*Setd8*	0.2593	Np	*St13*	0.3276	3.01E-04
Mab21l1	0.6271	0.6931	*Crispld1*	0.2594	0.4754	*Tbx15*	0.3283	0.0726
Smad9	0.6289	0.2326	*Ncor2*	0.2601	0.5021	*Pde7a*	0.3344	0.3313
Bloc1s3	0.6341	0.0623	*Slc31a1*	0.2617	0.4137	*Lonrf3*	0.337	0.2518
Arf4	0.6375	0.919	*Arvcf*	0.2629	0.5248	*Kcnd2*	0.3388	0.0996
Isg20l2	0.6413	0.8175	*Tmem55b*	0.2641	Np	*Grk6*	0.3408	0.945
Pmaip1	0.6431	0.5337	*Cdc42bpa*	0.2669	0.0129	*Myo1b*	0.3429	0.3162

Tfcp2	0.6433	0.5921	*Mafg*	0.2691	0.7709	*Taf12*	0.3429	0.051
Rrp36	0.6511	0.766	*Rab40c*	0.2697	0.2618	*Fry*	0.3449	0.0625
Dynll1	0.6631	0.872	*Scap*	0.2699	0.8086	*Gpr137b*	0.349	0.9969
Taf5l	0.6665	0.9622	*Tmem229b*	0.2712	0.5418	*B4galt5*	0.3495	0.1931
Luc7l2	0.6684	0.2009	*Suv420h2*	0.2725	Np	*Kit*	0.3503	0.1206
Arc	0.6778	0.1071	*Zfp384*	0.273	0.7914	*Rlim*	0.3512	0.354
Smg6	0.6792	0.6821	*Amot*	0.2797	0.8563	*Neurod4*	0.3542	0.37
Slc35e1	0.6798	0.2671	*Ywhae*	0.2803	0.492	*Lingo2*	0.3546	0.3475
Cdnf	0.6812	0.6739	*Erp44*	0.2812	0.2029	*Plxna3*	0.3558	0.6105
Cracr2b	0.6853	0.7214	*Klhdc3*	0.2814	0.2694	*Rnf150*	0.3558	0.8276
Rad1	0.6856	0.9004	*Ptprd*	0.2817	0.0775	*Aldh1a3*	0.356	0.8571
Hp1bp3	0.6875	0.7313	*Snx24*	0.284	0.5527	*Gxylt1*	0.3561	0.4006
Prr3	0.6887	0.4067	*Kdelc1*	0.2868	Np	*Dcp1a*	0.3566	7.37E-03
Ppih	0.6889	0.7222	*Luzp1*	0.2874	0.0072	*Rac1*	0.357	0.8657
Rarb	0.6932	0.656	*Pitpnm2*	0.2875	0.6314	*Slc17a6*	0.3572	0.3787
Zmat3	0.7043	0.0176	*Zfp366*	0.2881	0.7767	*Taf15*	0.3573	0.2889
Pofut1	0.7112	0.0153	*Fbxl20*	0.2902	0.116	*Wsb1*	0.3592	0.2018
March2	0.7148	0.8073	*Gjd2*	0.2925	0.3314	*En2*	0.3596	0.513
Zfp444	0.7157	0.6333	*Bak1*	0.2939	0.7623	*Zcchc7*	0.3597	0.4231
Suz12	0.7222	0.6103	*Dpp3*	0.2953	0.3468	*Pdcd6*	0.3618	0.5091
Tgfbr1	0.728	0.2906	*Zfp385c*	0.2953	0.1719	*Ptpn4*	0.3635	0.0669
Polr3gl	0.7337	0.4439	*Htr7*	0.2957	0.9681	*Atg14*	0.3638	0.674
Mrpl35	0.7349	0.133	*Metap2*	0.2986	0.6158	*Nr3c1*	0.3648	0.0242
Rinl	0.7382	0.6354	*Pten*	0.2995	0.0047	*Cd2ap*	0.3649	0.1179
Gnao1	0.7465	0.065	*Pole3*	0.3003	0.6692	*Kdm6a*	0.3656	0.9735
Tnrc6b	0.7481	0.6574	*Pdhx*	0.3021	0.4846	*Dab2ip*	0.3674	0.1724

Ebna1bp2	0.7484	0.8048	*Ctnnbip1*	0.3023	0.8977	*Dusp10*	0.3724	0.1714
Man2a2	0.7503	0.7848	*Prpf3*	0.3023	0.2691	*Zc3h6*	0.3729	0.1167
Gng2	0.7519	0.7651	*Mapre2*	0.3057	0.0537	*Tdrd7*	0.3798	0.8753
Sumo1	0.7608	0.5575	*Dlg2*	0.3108	0.7865	*Pdgfra*	0.38	0.0691
Slc25a51	0.762	0.2843	*Zhx3*	0.3116	0.2433	*Stc1*	0.3826	8.02E-03
Epm2aip1	0.7718	0.3217	*Bbc3*	0.3156	0.2457	*Klf11*	0.3909	0.2409
Mrpl17	0.7723	0.7168	*Cfl2*	0.3171	0.9368	*Edil3*	0.3924	0.0108
Klhl24	0.7779	0.6614	*Bahd1*	0.318	0.8311	*Fam196b*	0.3926	Np
Pkdcc	0.7845	0.1026	*Josd1*	0.3184	0.1951	*Tmx4*	0.3944	0.9149
Atad2b	0.7848	0.8929	*Kdm5c*	0.3192	0.077	*Msi2*	0.3944	0.7745
Map3k7	0.7899	0.7413	*Has2*	0.3218	0.1598	*Mark3*	0.3946	0.6895
Sema4b	0.7976	0.0917	*Larp4b*	0.3228	0.8902	*Kcnmb2*	0.3992	0.5074
Sema4g	0.8007	0.2769	*Vegfa*	0.3236	0.6414	*Gad2*	0.4003	0.9105
Prx	0.8008	0.1908	*Palm*	0.3266	0.4854	*Cacna1g*	0.402	0.37
Rpp14	0.8079	0.9773	*Atrx*	0.3272	0.1408	*Myo1c*	0.4041	0.4138
Ttn	0.8098	0.4326	*Dolpp1*	0.3295	0.3632	*Kdm2a*	0.4049	0.0497
Plk3	0.8107	0.1721	*Cdkl2*	0.3296	0.9597	*Zcchc2*	0.4052	8.89E-04
Trmt10a	0.8112	0.3917	*Edc3*	0.3313	0.2975	*Ube2k*	0.4066	0.1659
Mcc	0.8265	0.1413	*Dgkk*	0.3321	0.959	*Sik1*	0.4066	0.5614
Gmeb1	0.8301	0.6892	*Sidt1*	0.3337	0.0408	*Glis2*	0.408	0.1192
Supt7l	0.8313	0.1734	*Tanc1*	0.3343	0.0986	*Gatsl2*	0.4084	Np
Senp5	0.8342	0.2399	*Tet2*	0.3344	0.2849	*Necap1*	0.4102	0.7436
Nosip	0.845	0.9108	*Cnot6*	0.3347	0.7114	*Zzef1*	0.4107	0.2877
Esrrg	0.8458	0.2476	*Hip1*	0.3351	0.8762	*Cpsf6*	0.4113	0.5788
Bcs1l	0.8461	0.6246	*Pan2*	0.3381	0.5452	*Nfatc2*	0.4128	0.2687
Zfp780b	0.8463	0.8199	*Ret*	0.3389	0.8834	*Zbtb44*	0.4157	0.1098

N4bp2l2	0.8488	0.7671	*Mycn*	0.339	0.3334	*Sp4*	0.4193	0.9153
Rbm34	0.8595	0.9329	*Zbtb40*	0.3395	0.0083	*Zbtb34*	0.4195	0.2349
Slc9a1	0.8641	0.4737	*Ankrd49*	0.3412	0.4866	*Klf12*	0.4216	0.8291
Ldb2	0.8663	0.6041	*Hecw1*	0.3424	0.0053	*Ing3*	0.4225	5.90E-03
Zfhx2	0.8669	0.3699	*Fbxo42*	0.3429	0.5884	*Rrm2b*	0.4273	0.8939
Slc35e2	0.8753	0.0087	*Chsy1*	0.3438	0.249	*Pias2*	0.4288	1.99E-03
Kdm3b	0.8776	0.7805	*Ide*	0.3494	0.3136	*Klf4*	0.4301	0.9002
Ttc26	0.892	0.8388	*Rlim*	0.3512	0.354	*Stx17*	0.4313	0.3294
Gmfb	0.8957	0.4287	*Pxdn*	0.3588	0.8706	*Ppp1r14b*	0.4348	0.1012
Fgf9	0.9052	0.1593	*Prkg1*	0.3623	0.3902	*Pptc7*	0.4396	0.5962
Akap8	0.9059	0.7992	*Blmh*	0.3627	0.5675	*Mier3*	0.4407	0.3751
Rac2	0.9071	0.2906	*Wdfy1*	0.3633	0.4498	*Ccni*	0.4442	0.6012
Kdelc2	0.9115	Np	*Atg9a*	0.3634	0.2001	*Smc6*	0.4462	0.8265
Fndc5	0.9162	0.8211	*Tgfb3*	0.3636	0.6892	*Ppp1r9a*	0.4498	0.1313
Mtmr4	0.9182	0.036	*Dab2ip*	0.3674	0.1724	*Fam76b*	0.4505	0.9612
Azi2	0.9184	0.3464	*Tnfaip1*	0.3683	0.673	*Gigyf1*	0.4518	0.9844
Cyb5d2	0.9208	0.9559	*Shprh*	0.3687	0.0188	*Fundc1*	0.4567	0.5386
Shh	0.9216	0.1327	*Ppp1r13b*	0.3692	0.8485	*Baz1a*	0.4569	0.0868
Fam118a	0.9272	0.9201	*Col15a1*	0.3766	0.508	*Insig1*	0.4622	0.5135
Robo1	0.9335	0.5327	*Pdgfra*	0.38	0.0691	*Repin1*	0.4631	0.9434
Zbtb8a	0.9374	0.2371	*L3mbtl4*	0.38	0.4872	*Calcoco1*	0.4637	0.5521
Thoc2	0.9391	0.8661	*Dusp2*	0.3801	0.8683	*Dmrt2*	0.4639	0.8154
Itsn1	0.9509	0.8362	*Nfic*	0.3815	0.1648	*Med1*	0.4703	0.1052
Txnl1	0.9527	0.8511	*Suv420h1*	0.3823	Np	*Ptpn5*	0.4708	0.3193
Stam	0.9539	0.3669	*Baiap2*	0.3829	0.4691	*Celf2*	0.4728	0.6619
Fnbp1l	0.9564	0.4069	*Spen*	0.3874	0.2244	*Dexi*	0.4766	0.4968

Ncam1	0.9571	0.8507	*Tiam1*	0.3892	0.1171	*Satb2*	0.4781	0.0396
Akap5	0.9586	0.2878	*Ythdf1*	0.3914	0.2387	*Fam134c*	0.4785	Np
Dnmt3a	0.9639	0.821	*Mark3*	0.3946	0.6895	*Slc19a2*	0.4791	0.2465
Pfas	0.9669	0.7593	*Rlf*	0.3957	0.5317	*Luzp2*	0.487	0.1069
Xkrx	0.9679	0.9723	*Ireb2*	0.3964	0.6265	*Wdr12*	0.4891	0.9503
Dusp3	0.9693	0.0336	*Plekhf2*	0.397	0.2879	*Ube2z*	0.4924	0.7939
Klhl41	0.9695	0.9177	*Nebl*	0.4005	0.177	*Btaf1*	0.4927	0.2803
Inpp5e	0.9767	0.4617	*Phf21a*	0.4011	0.8672	*E2f6*	0.4952	0.0995
Zfp317	0.9788	0.3805	*Birc6*	0.4041	0.8527	*Smek1*	0.4995	Np
Plagl2	0.9794	0.1878	*Kdm2a*	0.4049	0.0497	*Dcx*	0.4995	0.1623
Wdr82	0.9801	0.3499	*Ube2k*	0.4066	0.1659	*Trhr*	0.5054	0.9477
Zdhhc3	0.9807	0.6435	*Sypl2*	0.4073	0.9338	*Hmgn3*	0.5056	0.0892
Tigd3	0.9819	0.1252	*Glis2*	0.408	0.1192	*Gpr88*	0.5101	0.5118
Zfp830	0.9824	0.8655	*Ccdc80*	0.4143	0.723	*Igf1*	0.5112	0.7392
Psmd11	0.9825	0.1811	*Vps26b*	0.4146	0.6504	*Sphk2*	0.5117	0.0246
Ctsa	0.9908	0.63	*Spry1*	0.4178	0.452	*Dock4*	0.5143	0.3169
Sfxn2	0.9943	0.1233	*Zbtb34*	0.4195	0.2349	*Jdp2*	0.5148	0.1072
Cwc25	0.9962	0.3294	*Insrr*	0.4196	0.8502	*Syncrip*	0.5149	0.1294
Cpeb3	0.9969	0.0159	*Mllt11*	0.42	0.7912	*Wwp2*	0.5172	0.6414
Sema3e	0.9972	0.1692	*Kpna4*	0.4211	0.465	*Baz2a*	0.5186	0.5403
D630023f18rik	Np	0.0013	*Klf12*	0.4216	0.8291	*Fam117b*	0.5213	0.5143
Ntf5	Np	0.1303	*Ahr*	0.4222	0.1354	*Ctdspl*	0.522	0.6534
Foxp3	Np	0.1623	*Pcdha5*	0.4255	0.7311	*Ube2h*	0.5232	7.22E-03
9330159f19rik	Np	0.1946	*Klf4*	0.4301	0.9002	*Megf11*	0.5255	0.2998
Foxl1	Np	0.2865	*Stx17*	0.4313	0.3294	*Elmod2*	0.5333	0.3966
Disp3	Np	0.4549	*Dot1l*	0.4325	0.8738	*Brd1*	0.5356	0.833

Slc6a20b	Np	0.4574	*Zer1*	0.4331	0.8912	*Maml1*	0.5366	0.5554
D17h6s53e	Np	0.5328	*Trib2*	0.4337	0.037	*Slc35a4*	0.54	0.3367
Smco1	Np	0.5895	*Smpd3*	0.4372	0.5774	*Kcna1*	0.5411	0.1158
Fbxo43	Np	0.6381	*Xkr7*	0.4393	0.5984	*Ppp2r5c*	0.5448	0.0441
Mybphl	Np	0.7471	*Cldn1*	0.4405	0.093	*Naa50*	0.5448	0.1595
Hoxa1	Np	0.7561	*Chfr*	0.4406	0.3868	*Ulk2*	0.5457	0.2838
Slc6a2	Np	0.8172	*Mier3*	0.4407	0.3751	*Eml6*	0.5458	0.4469
Zfp345	Np	0.851	*Kdm4a*	0.4409	0.6034	*Rhobtb1*	0.5486	0.6156
Gdf3	Np	0.8568	*Tubb2b*	0.441	0.1108	*Map3k1*	0.5512	0.223
P2ry4	Np	0.8771	*Gcsh*	0.4418	0.6121	*Agpat3*	0.5525	0.4481
Nmrk2	Np	0.8772	*Brwd3*	0.4423	0.2106	*Hnrnpa3*	0.5538	0.5826
Mrgprb1	Np	0.898	*Col2a1*	0.4439	0.9645	*Kctd12*	0.5542	0.3516
1110018g07rik	Np	Np	*Schip1*	0.4455	0.2217	*Scrt1*	0.5566	0.0536
2410066e13rik	Np	Np	*Fam76b*	0.4505	0.9612	*Tef*	0.5573	0.3367
3110035e14rik	Np	Np	*Mga*	0.4529	0.1735	*Slc30a4*	0.5574	5.10E-03
5133401n09rik	Np	Np	*Nanp*	0.4556	0.4463	*Stk38l*	0.558	0.4048
Bc023814	Np	Np	*Insig1*	0.4622	0.5135	*Map3k8*	0.5581	0.3268
D4ertd22e	Np	Np	*Lpl*	0.4633	0.8937	*Psmc6*	0.5585	0.4668
Galntl1	Np	Np	*Amfr*	0.4704	0.4782	*Zfp280d*	0.5614	0.0539
Gm11818	Np	Np	*Slc25a44*	0.4707	0.3067	*Csnk1g3*	0.5642	0.0185
Gm1587	Np	Np	*Celf2*	0.4728	0.6619	*Dip2c*	0.5706	0.6982
Gm749	Np	Np	*Jazf1*	0.4732	0.9184	*Clint1*	0.5802	0.705
Hoxb6	Np	Np	*Ap1g1*	0.4764	0.0025	*Ncoa2*	0.5812	6.80E-03
Myst3	Np	Np	*Morf4l2*	0.4767	0.9248	*Zmat4*	0.5822	0.0462
Pax9	Np	Np	*Fam134c*	0.4785	Np	*Trim33*	0.5864	0.0361
Tceb1	Np	Np	*Thoc1*	0.4822	0.237	*Snrk*	0.5888	0.1207

Tcfap4	Np	Np	*Ppard*	0.484	0.4151	*Tsn*	0.5891	0.9903
Cts8	Np	Np	*Hmcn1*	0.4862	0.1138	*Rictor*	0.59	0.2085
Gcnt3	Np	Np	*Mcl1*	0.4904	0.5499	*Pnkd*	0.5952	0.6854
Gata5	Np	Np	*Rab15*	0.4915	0.4419	*Pafah1b2*	0.597	0.0909
Aqp7	Np	Np	*Atp2b4*	0.4969	0.469	*Stk38*	0.5971	0.5326
Acer1	Np	Np	*Rnd1*	0.4969	0.096	*Slc25a5*	0.5984	0.5914
2810474o19rik	Np	Np	*Dcx*	0.4995	0.1623	*Raver2*	0.5988	0.4448
			Foxo3	0.5025	0.2464	*Kif3b*	0.6025	0.3937
			Rala	0.5032	0.0242	*Gins2*	0.6041	0.8816
			Chic2	0.5041	0.1486	*Ncf2*	0.6061	0.4491
			Hmgn3	0.5056	0.0892	*Maf*	0.6084	0.7132
			Arhgap36	0.5095	0.6416	*Plxna2*	0.6091	0.8531
			Ing2	0.5101	0.1932	*Zeb2*	0.6104	0.5947
			Igf1	0.5112	0.7392	*Snx25*	0.6129	0.4274
			Tbc1d13	0.5112	0.4444	*Rpgrip1l*	0.6201	0.0122
			Col4a1	0.5118	0.1218	*2810403a07rik*	0.6233	Np
			Sfpq	0.5122	0.3141	*Stac*	0.624	0.1658
			Syncrip	0.5149	0.1294	*Slc8a1*	0.6305	0.8715
			Frat2	0.5173	0.8629	*Unc79*	0.6317	0.1939
			Hnrnpul1	0.5195	0.5711	*Rpp40*	0.6343	0.4537
			Per1	0.5257	0.1055	*Ppp1cb*	0.6347	0.1017
			Frmd4a	0.5279	0.1695	*Mettl9*	0.6347	0.2645
			Slco5a1	0.5295	0.2002	*Mpzl3*	0.636	0.485
			Ccnl2	0.5333	0.7513	*Arf4*	0.6375	0.919
			Socs1	0.5334	0.1202	*Trpc3*	0.6389	0.0216
			Gpx7	0.5343	0.6992	*Tmem56*	0.6422	0.0928

			Thsd4	0.5371	0.1875	*Rap2c*	0.645	0.6976
			Scn3b	0.5404	0.5723	*Slc35a1*	0.6462	0.4365
			Dcaf12	0.5411	0.0353	*Phyhipl*	0.6467	0.1939
			Igsf1	0.5441	0.9156	*Ptgfrn*	0.6471	2.64E-03
			Eml6	0.5458	0.4469	*Diras2*	0.6474	0.1782
			Tnrc18	0.5465	0.6703	*Tbr1*	0.6522	0.295
			Rhobtb1	0.5486	0.6156	*Chm*	0.6528	0.1378
			Diablo	0.5516	0.4851	*Esrra*	0.654	0.1602
			Has3	0.5522	0.8717	*Ythdf3*	0.6543	0.0325
			Slc30a10	0.5544	0.1411	*Rab8a*	0.6592	0.1756
			Zfp26	0.5565	0.7699	*Tardbp*	0.6603	0.5435
			Kctd21	0.5569	0.0859	*Ccng2*	0.6607	0.0412
			Lif	0.5622	0.1483	*Papd7*	0.667	Np
			Ski	0.5624	0.6659	*Traf3*	0.6726	0.5687
			Kctd15	0.5679	0.5292	*Rnf4*	0.6748	0.0975
			Tpm1	0.5683	0.7418	*Asxl2*	0.6752	0.5741
			Pcgf3	0.5694	0.1474	*Thrb*	0.6768	0.3179
			Dip2c	0.5706	0.6982	*Nova1*	0.6772	0.1356
			Col27a1	0.5718	0.9228	*Ndst1*	0.68	0.9423
			Hdac4	0.5726	0.34	*Glyctk*	0.6838	0.3603
			Socs7	0.5754	0.802	*B3galt2*	0.6888	0.2177
			Atad5	0.5795	0.0799	*Styx*	0.689	0.5102
			Col4a2	0.5797	0.5164	*Cul4a*	0.6894	0.7284
			Ddx1	0.5841	0.1597	*Eps8*	0.6913	0.1382
			Nudt11	0.5849	0.1439	*Adcy1*	0.6945	0.0706
			Rictor	0.59	0.2085	*Slc24a3*	0.6954	0.6441

			Isl1	0.5916	0.9599	*Dtx2*	0.6966	0.9235
			Ctdspl2	0.5932	0.0672	*Fxyd6*	0.6993	0.2904
			Ing4	0.5932	0.3851	*Zfp704*	0.7049	0.9354
			Nras	0.5945	0.4833	*Lin7c*	0.7049	0.4515
			Spns1	0.5955	0.1371	*Coch*	0.7061	0.1402
			Adcyap1r1	0.5989	0.616	*Akt2*	0.7085	0.1242
			Nkiras2	0.5992	0.3132	*Rbm12*	0.7092	0.0499
			Adamts6	0.5994	0.5215	*Angpt1*	0.7093	0.0137
			Abce1	0.5994	0.4203	*Egr2*	0.7101	0.0367
			Bicd1	0.5994	0.209	*Med14*	0.7198	0.3377
			Capn7	0.6064	0.0127	*Clasp2*	0.7204	0.0555
			Strn3	0.6069	0.147	*Ahcyl1*	0.7247	0.3847
			Kctd5	0.6107	0.255	*Map2k4*	0.726	0.7314
			Nsd1	0.616	0.2436	*Azin1*	0.7295	0.0905
			Zfp91	0.6166	0.2407	*Sh3bp5*	0.7337	0.061
			Rnf214	0.6196	0.889	*Ank*	0.7359	0.1349
			Ina	0.621	0.0301	*Ltn1*	0.7432	0.0996
			Tspan14	0.6242	0.5425	*Zdhhc5*	0.7467	8.16E-03
			Ankrd13c	0.6246	0.8007	*Tnrc6b*	0.7481	0.6574
			Slc25a36	0.6263	0.5839	*Prickle1*	0.7487	0.07
			Eml4	0.628	0.4349	*Bcorl1*	0.7517	0.0845
			Ppp1cb	0.6347	0.1017	*Slc6a1*	0.7535	0.7474
			Rfx7	0.6382	0.5172	*Pla2g15*	0.7561	0.5826
			Etv4	0.6385	0.1035	*Stk11*	0.7589	0.9613
			Osbp	0.6444	0.3662	*Taok1*	0.7595	0.1997
			Pcdha6	0.6507	0.4471	*Rpl28*	0.7621	0.2406

			Hdgf	0.6534	0.2425	*Cops2*	0.767	0.1024
			Smarcc1	0.6542	0.0232	*Mxd1*	0.7692	0.0719
			Ythdf3	0.6543	0.0325	*Gjc1*	0.7699	0.5118
			Ank1	0.655	0.3392	*Paip1*	0.7752	0.5279
			Mest	0.6566	0.2819	*Zfp710*	0.7767	0.3143
			Rap1gds1	0.6579	0.2987	*Sv2a*	0.7791	0.2101
			Tll1	0.6585	0.0819	*Rims3*	0.7813	5.27E-03
			Tspan4	0.6608	0.1574	*Idh1*	0.7824	0.9207
			Col16a1	0.6612	0.0442	*Wnt7a*	0.7831	0.4356
			Nfat5	0.6666	0.9295	*Gpam*	0.7834	0.8206
			Cdc7	0.667	0.5367	*Stk35*	0.784	0.3639
			Col9a1	0.6695	0.7765	*Epha4*	0.785	1.99E-06
			Ctnnd1	0.6697	0.1018	*Chst10*	0.7873	0.7064
			Camta1	0.6726	0.0088	*Dnajb5*	0.7884	0.0691
			Traf3	0.6726	0.5687	*Furin*	0.7905	0.4408
			Bcl2l2	0.676	0.9946	*Zc3h3*	0.7908	0.0572
			Palm2	0.6788	Np	*Neurl3*	0.7969	0.4242
			Ndst1	0.68	0.9423	*Ppp4r2*	0.799	0.0854
			Ccdc88a	0.6813	0.5501	*Mbd6*	0.8008	0.3856
			Cspg4	0.6837	0.0802	*Prkaa1*	0.8071	0.9042
			Commd2	0.6841	0.5347	*Tcf4*	0.8073	0.1086
			Cps1	0.687	0.557	*Abhd6*	0.8139	0.2465
			Prr3	0.6887	0.4067	*Zfp326*	0.8177	0.1331
			Erp29	0.6924	0.3879	*Arf6*	0.8192	0.3895
			Snx4	0.6925	0.6493	*Slc39a5*	0.8202	0.934
			Rarb	0.6932	0.656	*Ash1l*	0.8218	0.1159

			Nkrf	0.6948	0.2534	*B3galnt2*	0.8269	0.3186
			Ppm1d	0.7034	0.4591	*Otud4*	0.8386	0.1517
			Zfp704	0.7049	0.9354	*Mbnl2*	0.8387	0.0159
			Fos	0.7049	0.0739	*Pde1b*	0.8424	0.7342
			Vps25	0.7059	0.1349	*Tab3*	0.8425	0.0378
			Mapkbp1	0.7066	0.7862	*Srsf6*	0.8426	0.276
			Cbx5	0.7097	0.4424	*Ppm1e*	0.8434	0.7212
			Pcdha10	0.7114	Np	*Fam78a*	0.8441	0.2865
			Zfyve26	0.7141	0.3107	*Nab2*	0.8452	0.1434
			Ibsp	0.7213	0.4849	*Fam126b*	0.8453	8.27E-03
			Arfgef2	0.7216	0.0683	*Esrrg*	0.8458	0.2476
			Ubfd1	0.7219	0.0721	*Ddx3x*	0.8464	0.7868
			Ankrd52	0.7221	0.1559	*Zbtb37*	0.8485	0.7076
			Adamts17	0.7249	0.849	*Rgs8*	0.8516	1.34E-03
			Map2k4	0.726	0.7314	*Gsk3b*	0.8548	0.053
			Mfap3	0.7268	0.139	*Cdc42*	0.8564	0.6928
			Nap1l3	0.7297	0.1302	*Dr1*	0.8581	0.401
			Ptprk	0.7298	0.0189	*Acsl6*	0.8598	0.7496
			Parg	0.7341	0.0796	*Arhgef18*	0.8614	0.0663
			Mrpl35	0.7349	0.133	*Dnajb1*	0.863	0.1488
			Bsdc1	0.741	0.31	*Glce*	0.8646	0.6269
			Zdhhc5	0.7467	0.0082	*Esf1*	0.8668	0.9918
			Tnrc6b	0.7481	0.6574	*Cpne8*	0.8718	0.9158
			Mbtd1	0.7504	0.9139	*Stk40*	0.8767	0.181
			Dicer1	0.7506	0.6452	*Tbx3*	0.8785	0.2969
			Gng2	0.7519	0.7651	*Wif1*	0.8834	0.1627

			Rev3l	0.7555	0.0729	*Grm5*	0.8849	0.9821
			Iffo2	0.7561	0.9178	*Ankrd28*	0.8877	0.5869
			Tmem135	0.7565	0.3394	*Herpud2*	0.8939	0.0463
			Cav2	0.7596	0.2998	*Smurf1*	0.8949	0.5934
			Hcn1	0.7609	0.5502	*Fam98a*	0.9004	0.7439
			Islr2	0.7629	0.6905	*Sox6*	0.901	0.8795
			Nufip2	0.7659	0.0679	*Ube2g1*	0.9037	0.0127
			Tmem86a	0.7692	0.0931	*Csdc2*	0.9061	0.4211
			Tmem132a	0.7735	0.498	*Cse1l*	0.9084	0.0438
			Dtx4	0.779	0.4934	*Fam168a*	0.9095	0.4431
			Col7a1	0.7803	0.4013	*Entpd7*	0.9115	0.1226
			Stk35	0.784	0.3639	*Nf1*	0.9145	0.8261
			Kcnip2	0.7842	0.5592	*Fndc5*	0.9162	0.8211
			Slc7a1	0.7847	0.0316	*Bcl11b*	0.9167	0.6817
			Atad2b	0.7848	0.8929	*Lmbr1l*	0.9177	0.8602
			Ypel2	0.785	0.0637	*Mtmr4*	0.9182	0.036
			Sh3rf3	0.7887	0.6457	*Htr2c*	0.9226	0.5208
			Adam19	0.7901	0.022	*Hlf*	0.9241	4.90E-03
			Phc1	0.7929	0.9143	*Zmym5*	0.9242	0.6436
			Pcdha8	0.7952	0.1689	*Vegfc*	0.9293	0.4618
			Epc1	0.7991	0.5859	*Impa2*	0.9346	0.3835
			Kctd20	0.8025	0.3273	*Tmem218*	0.9429	0.2061
			Tcf4	0.8073	0.1086	*Prkar2b*	0.9439	0.6809
			Zfp532	0.8113	0.4505	*Zbtb4*	0.9495	0.4113
			Ap4e1	0.8132	0.9372	*Cep170*	0.9558	0.0904
			Cdkn1a	0.8138	0.5044	*Fnbp1l*	0.9564	0.4069

			Scml2	0.8194	0.4096	*Dnmt3a*	0.9639	0.821
			Btg2	0.8252	0.6952	*Tsc22d2*	0.9687	0.1633
			Fam131b	0.8254	0.3487	*Kdm5b*	0.9781	0.2609
			Tmem127	0.827	0.0763	*Mapkapk2*	0.9789	0.736
			Npas4	0.8287	0.0689	*Zbtb7a*	0.9804	0.0443
			Unk	0.8315	0.8024	*Zdhhc3*	0.9807	0.6435
			Jarid2	0.8346	0.0231	*Lhfpl2*	0.9822	0.9773
			Otud4	0.8386	0.1517	*Chd9*	0.9894	0.5223
			Yy1	0.8389	0.1308	*Lrrn3*	0.9922	0.9394
			Lsm11	0.839	0.0496	*Zfp385a*	0.9923	0.3742
			Fem1b	0.8426	0.1764	*Hmgcll1*	0.9955	0.3899
			Ppm1e	0.8434	0.7212	*Gnat1*	Np	0.0278
			Fam126b	0.8453	0.0083	*Klhl10*	Np	0.2388
			Ddx3x	0.8464	0.7868	*Otc*	Np	0.268
			Morf4l1	0.8465	0.5674	*Slc17a3*	Np	0.3497
			Arnt	0.8518	0.8303	*Esrp1*	Np	0.3732
			N4bp2l1	0.8518	0.4809	*Spata16*	Np	0.3905
			Gsk3b	0.8548	0.053	*Aqp2*	Np	0.554
			Cdc42	0.8564	0.6928	*Nr1i3*	Np	0.5901
			Gfod1	0.858	0.033	*Gdf6*	Np	0.6244
			Trip12	0.8589	0.0669	*Afm*	Np	0.8212
			Ubtf	0.8599	0.3963	*Cngb3*	Np	0.8285
			Lcorl	0.8608	0.5537	*Dmrt3*	Np	0.8568
			Taf5	0.8609	0.3829	*Cd69*	Np	0.8568
			Prpf40a	0.8618	0.4094	*Sgcg*	Np	0.9391
			Nrbp1	0.863	0.3383	*Phf15*	Np	Np

			Hif3a	0.8679	0.1066	*Phf17*	Np	Np
			Cisd2	0.8703	0.3448	*4732418c07rik*	Np	Np
			Cpne8	0.8718	0.9158	*Csda*	Np	Np
			Slmap	0.8748	0.2353	*Jhdm1d*	Np	Np
			Hbegf	0.8753	0.46	*Mll1*	Np	Np
			Arl4a	0.876	0.0297	*Pppde1*	Np	Np
			Setdb1	0.883	0.5868	*Prosapip1*	Np	Np
			Col5a1	0.8853	0.0349	*Zfp295*	Np	Np
			Sirt1	0.8866	0.6204	*Au019823*	Np	Np
			Fam57b	0.8872	0.1797	*Au022870*	Np	Np
			Prkra	0.8886	0.1782	*Bai3*	Np	Np
			Cenpb	0.8907	0.1608	*Bc024479*	Np	Np
			Pigm	0.8932	0.8269	*D0h4s114*	Np	Np
			Zbtb43	0.8949	0.6454	*D19wsu162e*	Np	Np
			Gmfb	0.8957	0.4287	*D4bwg0951e*	Np	Np
			Rbfox2	0.9108	0.5495	*D4ertd22e*	Np	Np
			Entpd7	0.9115	0.1226	*Obfc2a*	Np	Np
			Bcl11b	0.9167	0.6817	*Eif3j*	Np	Np
			Dgkh	0.917	0.269	*Hnrpdl*	Np	Np
			Arf2	0.9174	0.2316	*1190002n15rik*	Np	Np
			Mtmr4	0.9182	0.036	*1200016b10rik*	Np	Np
			Htr2c	0.9226	0.5208	*2610030h06rik*	Np	Np
			Stau1	0.9256	0.0854	*2900011o08rik*	Np	Np
			Fubp1	0.9277	0.8239	*Bc017647*	Np	Np
			Srgap2	0.9304	0.6118	*Bc032203*	Np	Np
			Ank3	0.9311	0.2258	*Btbd12*	Np	Np

			Pdik1l	0.9328	0.9887	*D18ertd653e*	Np	Np
			Dennd1b	0.933	0.3013	*Dos*	Np	Np
			Robo1	0.9335	0.5327	*Eif2c4*	Np	Np
			Fam184b	0.9369	0.3143	*Fam123b*	Np	Np
			Col5a3	0.9384	0.8099	*Fam18b*	Np	Np
			Pitpnb	0.9384	0.9743	*Hoxd10*	Np	Np
			Pdgfb	0.9391	0.9533	*Pppde2*	Np	Np
			Thoc2	0.9391	0.8661	*Tcfap2a*	Np	Np
			Col11a1	0.951	0.2577	*Tcfap2c*	Np	Np
			Csnk1g1	0.9525	0.6176	*Tmem188*	Np	Np
			Eif4e2	0.9572	0.3401	*Zfp238*	Np	Np
			Ppip5k2	0.9576	0.123	*Au040829*	Np	Np
			Purg	0.9581	0.759	*Bc065085*	Np	Np
			Urm1	0.959	0.2577			
			Kif26b	0.9626	0.5019			
			Dnmt3a	0.9639	0.821			
			Sh3bp5l	0.967	0.4563			
			Rab14	0.9686	0.1631			
			Pex5	0.9691	0.1515			
			Dio2	0.9703	0.6056			
			Hapln1	0.976	0.6748			
			Pdgfa	0.9761	0.9096			
			Kdm5b	0.9781	0.2609			
			Plagl2	0.9794	0.1878			
			Klhl28	0.9805	0.0012			
			Gcc2	0.989	0.1167			

			Tmem151b	0.9907	0.4088			
			Foxj2	0.9928	0.303			
			Cpeb3	0.9969	0.0159			
			Rnf19a	0.9996	0.6797			
			Ptx3	Np	0.013			
			4931428f04rik	Np	0.0775			
			Tbx21	Np	0.1284			
			Trim63	Np	0.1319			
			Fasl	Np	0.1332			
			Pou4f1	Np	0.1482			
			D630045j12rik	Np	0.1853			
			Gm14137	Np	0.2324			
			Icos	Np	0.2709			
			Mat1a	Np	0.3263			
			Loxl4	Np	0.445			
			Calcr	Np	0.4611			
			Tectb	Np	0.5287			
			B230219d22rik	Np	0.5304			
			Phldb3	Np	0.5757			
			G6pc	Np	0.681			
			4921536k21rik	Np	0.6941			
			4931406p16rik	Np	0.7262			
			9530068e07rik	Np	0.7906			
			Lmx1a	Np	0.8145			
			Slc6a2	Np	0.8172			
			Atp1b4	Np	0.8568			

			Ifng	Np	0.8768			
			Hnf4g	Np	0.898			
			Tmc5	Np	0.9005			
			4921524j17rik	Np	0.9443			
			En1	Np	0.968			
			Hoxc13	Np	0.9792			
			4833424o15rik	Np	Np			
			Csda	Np	Np			
			Drd1a	Np	Np			
			Pvrl1	Np	Np			
			Rod1	Np	Np			
			Smek2	Np	Np			
			Tcfec	Np	Np			
			2310046o06rik	Np	Np			
			2400001e08rik	Np	Np			
			2810046l04rik	Np	Np			
			4933439f18rik	Np	Np			
			C77370	Np	Np			
			D0h4s114	Np	Np			
			D19wsu162e	Np	Np			
			D3bwg0562e	Np	Np			
			Eomes	Np	Np			
			Fam123b	Np	Np			
			Fam70a	Np	Np			
			Ifi30	Np	Np			
			Smcr7l	Np	Np			

			Spnb2	Np	Np			
			Srcrb4d	Np	Np			
			Tcfeb	Np	Np			
			6720456h20rik	Np	Np			
			8030411f24rik	Np	Np			
			8430427h17rik	Np	Np			
			Au019823	Np	Np			
			Bat2l2	Np	Np			
			C030046e11rik	Np	Np			
			C030046i01rik	Np	Np			
			Epb4.1l4b	Np	Np			
			Fam116a	Np	Np			
			Gcap14	Np	Np			
			Hiatl1	Np	Np			
			Lep	Np	Np			
			Orf61	Np	Np			
			Spna2	Np	Np			
			Tcfap2c	Np	Np			
			Tmem48	Np	Np			
			Zfp191	Np	Np			
			Bc035295	Np	Np			
			Bc060632	Np	Np			
			Bc065085	Np	Np			
			C330019g07rik	Np	Np			
			Mtap6	Np	Np			

Table S4. Significantly enriched KEGG pathways from all downregulated miRNAs target genes

Description	Overlap	pValue	FDR
Focal adhesion	106	8.94E-10	8.94E-08
Pathways in cancer	177	3.18E-09	1.06E-07
FoxO signaling pathway	74	3.71E-09	1.06E-07
Proteoglycans in cancer	103	4.23E-09	1.06E-07
MAPK signaling pathway	120	1.81E-08	3.62E-07
Axon guidance	70	2.59E-08	4.32E-07
Regulation of actin cytoskeleton	104	8.47E-08	1.21E-06
PI3K-Akt signaling pathway	153	2.27E-07	2.84E-06
Ras signaling pathway	106	7.35E-07	8.17E-06
Thyroid hormone signaling pathway	61	1.27E-06	1.27E-05
Signaling pathways regulating pluripotency of stem cells	70	1.63E-06	1.48E-05
ErbB signaling pathway	47	5.92E-06	4.93E-05
Neurotrophin signaling pathway	61	7.53E-06	5.79E-05
Cell cycle	62	8.85E-06	5.91E-05
TNF signaling pathway	56	8.86E-06	5.91E-05
Adherens junction	41	1.01E-05	6.31E-05
Prostate cancer	47	1.33E-05	7.82E-05
HTLV-I infection	120	1.53E-05	0.000085
Endometrial cancer	31	1.72E-05	9.05E-05
Hippo signaling pathway	72	0.000024	0.00012
HIF-1 signaling pathway	54	3.45E-05	0.000164
Renal cell carcinoma	37	4.65E-05	0.000211
Colorectal cancer	35	6.45E-05	0.000272
Insulin resistance	54	6.52E-05	0.000272
DNA replication	22	9.53E-05	0.000381
Dorso-ventral axis formation	17	0.000148	0.000563
Choline metabolism in cancer	49	0.000152	0.000563
Rap1 signaling pathway	91	0.000192	0.000686
Toxoplasmosis	53	0.000249	0.000859
Sphingolipid signaling pathway	57	0.000287	0.000957
Fanconi anemia pathway	28	0.000314	0.001006
Bacterial invasion of epithelial cells	39	0.000322	0.001006
Hepatitis B	65	0.000343	0.001009
Non-small cell lung cancer	30	0.000343	0.001009
Pancreatic cancer	34	0.000377	0.001077
Epstein-Barr virus infection	90	0.000396	0.0011
AMPK signaling pathway	58	0.000516	0.001395
Ubiquitin mediated proteolysis	63	0.000592	0.001558
VEGF signaling pathway	31	0.000632	0.001605
Glioma	33	0.000642	0.001605
Salmonella infection	38	0.000733	0.001788
Endocytosis	111	0.000764	0.001819
Platelet activation	58	0.000827	0.001923
Steroid biosynthesis	13	0.000849	0.00193
Phosphatidylinositol signaling system	45	0.000949	0.002109
N-Glycan biosynthesis	26	0.00102	0.002217
Lysosome	55	0.00106	0.002255

Insulin signaling pathway	61	0.00125	0.002592
Small cell lung cancer	40	0.00127	0.002592
ECM-receptor interaction	41	0.00141	0.002765
Fc gamma R-mediated phagocytosis	41	0.00141	0.002765
Osteoclast differentiation	55	0.00166	0.003151
Gap junction	40	0.00167	0.003151
Adrenergic signaling in cardiomyocytes	64	0.00218	0.004037
Toll-like receptor signaling pathway	45	0.0026	0.004727
p53 signaling pathway	32	0.0028	0.004965
Progesterone-mediated oocyte maturation	40	0.00283	0.004965
Regulation of lipolysis in adipocytes	28	0.00302	0.005186
Melanogenesis	44	0.00306	0.005186
Leishmaniasis	31	0.00333	0.00555
Chronic myeloid leukemia	34	0.00351	0.005754
Apoptosis	37	0.00361	0.005823
Wnt signaling pathway	60	0.00392	0.006222
NOD-like receptor signaling pathway	28	0.00414	0.006469
cAMP signaling pathway	79	0.00424	0.0065
Alanine, aspartate and glutamate metabolism	20	0.00429	0.0065
Central carbon metabolism in cancer	31	0.00444	0.006627
mTOR signaling pathway	29	0.0047	0.006912
GnRH signaling pathway	39	0.00543	0.007786
Adipocytokine signaling pathway	33	0.00545	0.007786
RNA degradation	37	0.00593	0.008352
Notch signaling pathway	24	0.00612	0.0085
Oxytocin signaling pathway	64	0.00639	0.008753
Phospholipase D signaling pathway	59	0.00648	0.008757
Jak-STAT signaling pathway	65	0.00657	0.00876
B cell receptor signaling pathway	33	0.00704	0.009263
Estrogen signaling pathway	42	0.00817	0.01061
Rheumatoid arthritis	36	0.00882	0.011111
Glutamatergic synapse	48	0.00889	0.011111
Prolactin signaling pathway	33	0.009	0.011111
Pertussis	33	0.009	0.011111
cGMP-PKG signaling pathway	68	0.00966	0.01178
Sphingolipid metabolism	23	0.00999	0.012036
Peroxisome	36	0.011	0.013095
Fc epsilon RI signaling pathway	30	0.0116	0.013647
Bladder cancer	20	0.0125	0.014368
Inositol phosphate metabolism	31	0.0125	0.014368
Synaptic vesicle cycle	28	0.0127	0.014432
Other types of O-glycan biosynthesis	16	0.013	0.014607
One carbon pool by folate	11	0.0134	0.014889
Hypertrophic cardiomyopathy (HCM)	36	0.0137	0.015055
TGF-beta signaling pathway	36	0.0168	0.018261
Mismatch repair	12	0.018	0.019355
Influenza A	66	0.0191	0.019896
Glycerophospholipid metabolism	39	0.0191	0.019896
Long-term depression	27	0.0191	0.019896
Glycosylphosphatidylinositol(GPI)-anchor biosynthesis	13	0.0226	0.023232

Vasopressin-regulated water reabsorption	20	0.0228	0.023232
Fatty acid metabolism	23	0.023	0.023232
Cholinergic synapse	45	0.0273	0.0273

Table S5. Results of gene-based association analysis on BMI for the target genes of miR-665-3p and miR-29c-3p.

Gene symbol	Target genes miR-665-3p	Target genes miR-29c-3p	p-value BMI
EFR3B	x		**1.57E-43**
RBFOX1	x		**9.40E-32**
NUP160		x	**3.67E-31**
NCKAP5L		x	**8.28E-27**
SPNS1		x	**8.24E-25**
PTPRD		x	**1.40E-21**
HMGCR		x	**5.58E-20**
DLG2		x	**9.62E-20**
EFNA5		x	**3.81E-17**
CAMTA1		x	**9.80E-17**
FAM57B		x	**3.60E-16**
FOXO3		x	**7.20E-16**
TRAF3		x	**1.53E-15**
PRKG1		x	**4.27E-15**
NAV1		x	**1.55E-14**
NFAT5		x	**2.35E-14**
TNRC6B	x	x	**3.34E-14**
ROBO1	x	x	**5.47E-14**
BCL7A	x	x	**5.68E-14**
TRIP12		x	**1.02E-13**
DNMT3A	x	x	**1.10E-13**
CALCR		x	**1.24E-13**
KNOP1	x		**1.72E-13**
HIP1		x	**4.10E-13**
COL16A1		x	**4.31E-13**
RARB	x	x	**4.71E-13**
MARK3		x	**5.55E-13**
PURG		x	**8.72E-13**
BCL11B		x	**1.52E-12**
BAIAP2		x	**1.54E-12**
HIVEP2	x		**2.74E-12**
N4BP2L2	x		**2.99E-12**
DCAF7		x	**7.49E-12**
DIABLO		x	**1.01E-11**
MCC	x		**1.09E-11**
PIK3R3		x	**1.56E-11**
LOXL4		x	**1.58E-11**
PPARD		x	**1.68E-11**
LDB2	x		**2.20E-11**
MLLT11		x	**3.15E-11**
EXT1	x		**4.49E-11**
KCNMA1	x		**4.96E-11**
HBEGF		x	**6.77E-11**
SMPD3		x	**6.93E-11**
FIGN		x	**7.45E-11**

GALNTL1	x		**7.88E-11**
CBX5		x	**8.47E-11**
AKT3	x	x	**9.02E-11**
NCAM1	x		**9.37E-11**
TSPAN4		x	**1.01E-10**
LUZP1	x	x	**1.76E-10**
SMG6	x		**1.92E-10**
BAK1		x	**2.01E-10**
URM1		x	**2.03E-10**
PPP1CB		x	**3.16E-10**
BAT2L2		x	**4.26E-10**
PHF13	x		**4.51E-10**
FNDC5	x		**4.96E-10**
MFAP3		x	**5.17E-10**
CHD3	x		**5.96E-10**
UBTF		x	**6.19E-10**
DAB1		x	**6.55E-10**
DAG1		x	**8.15E-10**
N4BP2L1		x	**8.95E-10**
MGA	x	x	**9.02E-10**
FREM1		x	**9.36E-10**
DOCK3	x		**2.13E-09**
ERP44		x	**2.26E-09**
SLC25A22		x	**2.30E-09**
SMARCC1		x	**2.47E-09**
POFUT1	x		**3.03E-09**
KCTD15		x	**3.10E-09**
ANK3		x	**3.23E-09**
MYBPHL	x		**4.12E-09**
ZDHHC21		x	**4.83E-09**
BCS1L	x		**5.44E-09**
MFAP2		x	**5.55E-09**
TMOD3		x	**5.63E-09**
HAS3		x	**5.95E-09**
DCAF12		x	**6.86E-09**
DCUN1D4		x	**6.98E-09**
NASP		x	**7.31E-09**
HNF4G		x	**1.14E-08**
EDNRB		x	**1.21E-08**
ABCE1		x	**1.69E-08**
NRAS		x	**1.78E-08**
DAB2IP		x	**1.85E-08**
VCL		x	**1.93E-08**
HOXB6	x		**2.09E-08**
KDM3B	x		**3.16E-08**
ARFGEF2		x	**3.45E-08**
PPP1R13B		x	**3.60E-08**
PLAGL2	x	x	**3.81E-08**
POU2F2	x	x	**3.93E-08**
TANC1		x	**4.89E-08**

DNASE2	x		**5.03E-08**
KIAA0408	x		**5.09E-08**
SP1		x	**5.42E-08**
DACH1	x		**9.53E-08**
DGKH		x	**1.01E-07**
MICALL1	x		**1.11E-07**
ABCB6		x	**1.12E-07**
STK35		x	**1.13E-07**
SUFU	x		**1.28E-07**
NCAN	x		**1.37E-07**
CRACR2B	x		**1.90E-07**
CHFR		x	**2.05E-07**
CYB561D1		x	**2.06E-07**
FRMD5		x	**2.17E-07**
SPRY4		x	**2.78E-07**
ARVCF	x	x	**2.91E-07**
JAZF1		x	**2.93E-07**
KLHL24	x		**3.49E-07**
AHR		x	**3.63E-07**
BYSL	x		**3.73E-07**
CCNT2	x	x	**3.83E-07**
SYPL2		x	**3.89E-07**
SYNCRIP		x	**4.02E-07**
MMP24	x	x	**4.13E-07**
UNK		x	**4.43E-07**
OTUD4		x	**4.46E-07**
TNRC18		x	**4.97E-07**
ARHGAP19	x		**5.15E-07**
CACNB3	x		**5.53E-07**
ATG9A		x	**6.23E-07**
TMEM263	x		**6.87E-07**
GSK3B		x	**7.03E-07**
CISD2		x	**1.02E-06**
ZBTB7B	x		**1.26E-06**
CELF2		x	**1.33E-06**
CPS1		x	**1.35E-06**
ISL1		x	**1.38E-06**
L3MBTL4		x	**1.43E-06**
NUCKS1	x		**1.61E-06**
MAP4K4		x	**1.66E-06**
KDM5B		x	**2.52E-06**
CCDC62	x		3.08E-06
ABI2	x		3.38E-06
FERMT2		x	3.60E-06
PPIP5K2		x	3.64E-06
TMC5		x	5.00E-06
GNAO1	x		5.17E-06
COL4A4		x	5.85E-06
ADAMTS6		x	5.94E-06
MRTFB	x		6.08E-06

FAM134C		x	6.60E-06
TIAM1		x	6.79E-06
SUPT7L	x		7.51E-06
SRC		x	7.63E-06
ZBTB43		x	7.82E-06
AAK1	x		8.04E-06
SETD5		x	8.43E-06
SFXN2	x		8.85E-06
ANKRD13B		x	9.16E-06
TM9SF4	x		1.00E-05
RND1		x	1.06E-05
SMARCD2	x		1.09E-05
QK		x	1.17E-05
KLF12		x	1.19E-05
TSPAN9		x	1.19E-05
LPP	x		1.20E-05
JARID2		x	1.35E-05
PDHX		x	1.35E-05
MYST3	x		1.46E-05
RFXANK	x		1.47E-05
ZBTB34	x	x	1.56E-05
COL1A2		x	1.91E-05
PTPN2	x		1.92E-05
VEGFA		x	1.95E-05
NEBL		x	2.12E-05
CPEB3	x	x	2.16E-05
ARHGAP28	x	x	2.20E-05
RAB15		x	2.28E-05
SGK1		x	2.28E-05
MEST		x	2.44E-05
SLC31A1		x	2.48E-05
SUZ12	x		2.53E-05
DPYSL5	x	x	2.57E-05
RAPGEFL1		x	2.58E-05
EML6		x	2.75E-05
DNM3		x	2.78E-05
BCL2L2		x	2.81E-05
NFIA		x	2.99E-05
COPS7B	x		3.64E-05
PNPLA3	x		4.02E-05
PRPF3		x	4.03E-05
TAPBP	x		4.10E-05
RFX7		x	4.16E-05
STAU1		x	4.37E-05
FRMD4A		x	4.43E-05
XKR4		x	4.49E-05
ECM2	x		4.77E-05
HDAC4		x	5.12E-05
SLC6A20	x		5.53E-05
RYBP		x	5.62E-05

BLMH		x	6.05E-05
TRAF4	x	x	6.50E-05
RRAGD	x		6.70E-05
SCHIP1		x	6.86E-05
ZFP26		x	7.69E-05
MIER3		x	9.01E-05
BMF		x	9.80E-05
PTPN11		x	9.94E-05
ANKRD49		x	1.01E-04
TRIM37		x	1.04E-04
ZBTB6	x		1.06E-04
DECR2	x		1.08E-04
TBL1XR1	x		1.09E-04
PSD2	x		1.11E-04
ETV4		x	1.13E-04
MTMR4	x	x	1.18E-04
VASH2		x	1.39E-04
POU4F1		x	1.40E-04
ESRRG	x		1.41E-04
TNFAIP1		x	1.42E-04
RHOBTB1		x	1.45E-04
NDRG4		x	1.55E-04
ZNF3	x		1.56E-04
KDM6B		x	1.58E-04
BICD1		x	1.66E-04
DUSP2		x	1.80E-04
WDR82	x		1.90E-04
LPL		x	1.92E-04
IL1RAP		x	1.96E-04
SETDB1		x	2.02E-04
SLC2A4	x		2.03E-04
FAM118A	x		2.03E-04
SOX12		x	2.12E-04
NLK	x		2.24E-04
RPP14	x		2.29E-04
PMAIP1	x		2.29E-04
CMPK1		x	2.39E-04
PCDHA1		x	2.66E-04
NANP		x	2.92E-04
LCORL		x	3.04E-04
MAPKBP1		x	3.44E-04
STRN3		x	3.79E-04
SLC38A2		x	3.82E-04
SEMA3E	x		3.88E-04
TIMMDC1	x		4.16E-04
NCL	x		4.28E-04
PPM1E		x	4.46E-04
SCAMP5	x	x	4.53E-04
REV3L		x	4.55E-04
GRIN2B	x		4.76E-04

IFI30		x	4.96E-04
CERCAM	x		5.02E-04
SERF2	x		5.03E-04
CRAMP1	x		5.08E-04
BACH2		x	5.09E-04
PCDHA2		x	5.15E-04
RND3		x	5.33E-04
CAMK1D		x	5.50E-04
SUMO1	x		5.69E-04
SNX24		x	5.72E-04
EML5		x	5.73E-04
FBXO42		x	5.73E-04
DPP4		x	5.99E-04
SYT9		x	6.03E-04
MAGI1	x		6.18E-04
ARNT		x	6.18E-04
LIMCH1	x		6.29E-04
KLHL41	x		6.35E-04
PCDHA3		x	6.36E-04
CCDC127	x		7.56E-04
F11R		x	7.59E-04
DIO2		x	7.77E-04
DPP3		x	7.82E-04
SIRT1		x	7.90E-04
RET		x	8.12E-04
LACTB2		x	8.28E-04
NCKAP5		x	8.42E-04
SNX4		x	8.56E-04
SMCO1	x		8.59E-04
PIK3R1		x	9.39E-04
DAAM1		x	9.41E-04
CD34	x		9.61E-04
KCTD5		x	9.77E-04
PTP4A1		x	9.88E-04
TEX9	x		9.91E-04
TBX21		x	1.07E-03
RLF		x	1.08E-03
IFNAR1	x		1.08E-03
STRBP	x		1.11E-03
TLL1		x	1.13E-03
TCF4		x	1.17E-03
ADAM12		x	1.18E-03
TRIB2		x	1.21E-03
PKNOX2		x	1.21E-03
PPP1R9A	x		1.22E-03
PRELP		x	1.27E-03
EPS15		x	1.46E-03
DENND1B		x	1.49E-03
CANX		x	1.56E-03
SH3BP5L		x	1.59E-03

MRPL11	x		1.60E-03
PCDHA4		x	1.65E-03
CHSY1		x	1.80E-03
ANKRD34A	x		1.80E-03
BSDC1		x	2.00E-03
EML4		x	2.03E-03
COL4A2		x	2.04E-03
GFOD1		x	2.09E-03
ELF2		x	2.16E-03
CAPN7		x	2.19E-03
CYB5D2	x		2.25E-03
CEP97	x		2.27E-03
CAMK2G		x	2.38E-03
STX17	x	x	2.41E-03
SLC22A5		x	2.42E-03
SEPT3	x		2.44E-03
XPO7	x		2.57E-03
NFIC		x	2.63E-03
BIRC6		x	2.65E-03
CSPG4		x	2.74E-03
CAPN5	x		2.79E-03
GTF2A1	x		2.96E-03
MAPK14	x		2.99E-03
PFAS	x		3.04E-03
MBTD1		x	3.04E-03
IDE		x	3.05E-03
GMEB1	x		3.05E-03
COL7A1		x	3.13E-03
TOX4	x		3.16E-03
ADAMTS17		x	3.17E-03
SAP18	x		3.18E-03
NRSN1		x	3.33E-03
AZI2	x		3.41E-03
PEX13	x		3.47E-03
MROH1	x		3.61E-03
ZCCHC17	x		3.83E-03
PPIC		x	3.85E-03
LIN7A		x	3.87E-03
LSM11		x	3.89E-03
APLP2	x		3.91E-03
HPCAL4		x	4.19E-03
DYNLL1	x		4.27E-03
PCDHA5		x	4.33E-03
ATXN7L3	x		4.50E-03
ITGB1		x	4.56E-03
ITGA11		x	4.66E-03
NSD1		x	4.81E-03
TMEM127		x	5.02E-03
PCDHAC2		x	5.14E-03
FRAS1		x	5.37E-03

SCAP		x	5.43E-03
CAV2		x	5.45E-03
METAP2		x	5.64E-03
STON2	x		5.85E-03
RAB30		x	5.87E-03
TECTB		x	6.02E-03
PSMD11	x		6.41E-03
NCOA4		x	6.43E-03
AMFR		x	6.75E-03
MAT1A		x	6.83E-03
CDKL2		x	7.00E-03
PCLAF	x		7.10E-03
GTPBP3	x		7.27E-03
EMC1	x		7.32E-03
KIF26A		x	7.39E-03
COL4A3		x	7.53E-03
SPEN		x	7.56E-03
FNDC3B	x		7.58E-03
PXDN		x	7.63E-03
GPR85		x	7.66E-03
TMEM151B		x	7.72E-03
PCDHA6		x	7.78E-03
LDLRAD3		x	7.86E-03
CPNE8		x	7.90E-03
AP1G1	x	x	8.07E-03
THOC1		x	8.46E-03
KCNK6	x		8.54E-03
HEPACAM		x	8.58E-03
ZNF420	x		8.59E-03
PITPNM3		x	8.83E-03
MAPRE2	x	x	8.89E-03
SPRY1		x	9.20E-03
RAP1GDS1		x	9.39E-03
SKI		x	9.51E-03
MLST8	x		9.52E-03
ATAD2B	x	x	9.61E-03
PAXIP1	x		9.67E-03
HIF3A		x	9.83E-03
MORF4L1		x	9.94E-03
GPX7		x	1.00E-02
COL15A1		x	1.07E-02
MLF1		x	1.09E-02
TRIM44	x		1.09E-02
PLEKHF2		x	1.09E-02
BLOC1S3	x		1.10E-02
KCNC2		x	1.11E-02
COL2A1		x	1.12E-02
ZFYVE26		x	1.15E-02
CDKN1A		x	1.16E-02
PCDHAC1		x	1.18E-02

SEMA4G	x		1.21E-02
PRDX1	x		1.22E-02
MTAP6		x	1.23E-02
CNOT6		x	1.32E-02
PLEKHA1		x	1.35E-02
RAB14		x	1.37E-02
PLXNA2	x		1.37E-02
NRF1	x		1.38E-02
KIF26B		x	1.42E-02
KCNIP2		x	1.44E-02
FRMD4B	x		1.52E-02
AGPS	x		1.62E-02
VEZF1	x		1.65E-02
SLMAP		x	1.65E-02
GNG12		x	1.67E-02
STX1A		x	1.69E-02
PLEKHH1	x		1.70E-02
AKAP5	x		1.70E-02
DPYSL2		x	1.71E-02
IBSP		x	1.72E-02
FADD	x		1.75E-02
PIN1	x		1.78E-02
MAT2A		x	1.83E-02
GPR37		x	1.84E-02
LEMD3		x	1.85E-02
MBLAC2		x	1.87E-02
SHPRH		x	1.87E-02
SENP5	x		1.88E-02
CD276		x	1.92E-02
COL27A1		x	1.93E-02
NKTR		x	1.94E-02
KL	x		1.94E-02
SERINC5		x	1.98E-02
CTSA	x		2.01E-02
MRPL35	x	x	2.02E-02
STRN4		x	2.06E-02
PCDHA8		x	2.11E-02
PCDHA11		x	2.11E-02
ZBTB8A	x		2.12E-02
PCDHA7		x	2.13E-02
SLC6A2	x	x	2.15E-02
PAX9	x		2.15E-02
SIDT1		x	2.23E-02
PCDHA10		x	2.32E-02
ZDHHC5		x	2.36E-02
PRR5L		x	2.41E-02
DTX4		x	2.42E-02
GIT2	x		2.45E-02
NRCAM	x		2.45E-02
YWHAE		x	2.47E-02

ZCCHC2	x		2.49E-02
ABHD4	x	x	2.49E-02
CSNK1G1		x	2.49E-02
DTNA	x		2.52E-02
VAMP3	x	x	2.52E-02
ZBTB5		x	2.53E-02
SLC4A4	x		2.57E-02
ARF4	x		2.60E-02
VASH1		x	2.61E-02
MRPL17	x		2.67E-02
ADAMTS18		x	2.73E-02
LMX1A		x	2.89E-02
SNX22		x	2.90E-02
TGFB3		x	2.90E-02
ZFP346		x	2.93E-02
SEMA4B	x		2.96E-02
SLC25A44		x	2.99E-02
YY1		x	3.08E-02
LYSMD1		x	3.11E-02
ATAD5		x	3.18E-02
ROD1		x	3.19E-02
SUDS3	x		3.22E-02
RAC2	x		3.30E-02
ZDHHC3	x		3.35E-02
SLC15A1	x		3.38E-02
SRCIN1	x		3.39E-02
ENPP2		x	3.40E-02
OSTF1	x		3.45E-02
GDAP1L1	x		3.46E-02
MARK2	x		3.48E-02
NCOR2		x	3.49E-02
PCGF3		x	3.51E-02
TCFEC		x	3.54E-02
CTNND1		x	3.58E-02
EPC1		x	3.62E-02
GMFB	x	x	3.63E-02
MEGF6		x	3.67E-02
ATP2B4		x	3.79E-02
USP45	x		3.86E-02
CDNF	x		3.90E-02
NUFIP2		x	3.98E-02
HNRNPAB	x		4.04E-02
EPM2AIP1	x		4.07E-02
TRIM36	x		4.10E-02
GDPGP1	x		4.20E-02
RAB40C		x	4.22E-02
SLC20A1	x		4.23E-02
MCL1		x	4.30E-02
LARP4B		x	4.34E-02
GXYLT2		x	4.35E-02

FUBP1		x	4.36E-02
DIP2C		x	4.37E-02
AFAP1	x		4.44E-02
SERPINH1		x	4.49E-02
CTDSPL2		x	4.50E-02
RRP1B	x		4.53E-02
ITSN1	x		4.60E-02
TNFAIP6	x		4.61E-02
TBC1D13		x	4.69E-02
DAAM2		x	4.76E-02
DARS2	x		4.79E-02
DBT		x	4.80E-02
TMEM229B		x	4.81E-02
PAN2		x	4.84E-02
CREB5		x	4.89E-02
SMAD9	x		4.98E-02
SLC39A9		x	4.99E-02
TUBB2A		x	5.00E-02
ERCC2	x		5.11E-02
FSTL1		x	5.17E-02
PER1		x	5.17E-02
GNG2	x	x	5.21E-02
TMEM86A		x	5.30E-02
NID2		x	5.45E-02
AGAP2	x		5.48E-02
CDC42BPA		x	5.50E-02
KIF21B	x		5.53E-02
RALA		x	5.55E-02
ERI2	x		5.61E-02
SMEK2		x	5.68E-02
IMPDH1	x	x	5.73E-02
ANK1		x	5.74E-02
IGF1		x	5.76E-02
ARC	x		5.80E-02
COL11A1		x	5.81E-02
LIX1	x		5.81E-02
CTNNBIP1		x	5.83E-02
COMMD2		x	5.91E-02
ZNF317	x		6.02E-02
ONECUT2	x	x	6.07E-02
CNR1		x	6.11E-02
POLR3GL	x		6.11E-02
ADAMTS7		x	6.19E-02
LEKR1	x		6.29E-02
ANKRD13C	x	x	6.34E-02
PITPNM2		x	6.41E-02
DICER1		x	6.41E-02
PCTP	x		6.43E-02
FASL		x	6.44E-02
FNBP1L	x		6.67E-02

FGF9	x		6.71E-02
AFF4		x	6.81E-02
BMPR1A		x	6.82E-02
PTEN		x	6.90E-02
HIATL1		x	6.91E-02
FEM1B		x	6.95E-02
LIF		x	6.99E-02
DOT1L		x	7.01E-02
STEAP4	x		7.11E-02
SLC16A1		x	7.19E-02
LMTK2		x	7.25E-02
FAM126B		x	7.36E-02
STAM	x		7.42E-02
FAM116A		x	7.44E-02
SGMS2	x		7.63E-02
EID1	x		7.70E-02
VPS26B		x	7.81E-02
WDFY1		x	7.81E-02
DDHD2	x		7.92E-02
ELOVL4		x	7.93E-02
LAMC1		x	7.93E-02
XPO1	x		7.97E-02
UBAC1	x		8.06E-02
SCN8A	x		8.26E-02
SIX4	x		8.29E-02
BNIP3L	x		8.31E-02
BCAM	x		8.40E-02
BTG2		x	8.51E-02
FAM184B		x	8.57E-02
BBC3	x	x	8.60E-02
ANKRD52		x	8.70E-02
IFFO2		x	8.92E-02
ABI1		x	8.96E-02
GK5	x		9.06E-02
E2F7		x	9.17E-02
ATRN		x	9.19E-02
ZNF385C	x		9.20E-02
FRS2	x		9.33E-02
NUP214	x		9.64E-02
TMEM201	x	x	9.65E-02
FBXO43	x		9.74E-02
PIGM		x	9.75E-02
YTHDF3		x	1.01E-01
TTN	x		1.02E-01
FAM19A1	x		1.02E-01
STMN2		x	1.03E-01
KDELC1		x	1.06E-01
IBA57	x		1.06E-01
PMP22		x	1.07E-01
IREB2		x	1.07E-01

TCFEB		x	1.09E-01
KIRREL		x	1.09E-01
RCOR1		x	1.10E-01
MRPL40	x		1.12E-01
TOR1AIP2	x		1.12E-01
HAPLN1		x	1.15E-01
CBFA2T2	x		1.15E-01
UBE2N	x		1.15E-01
HBP1		x	1.16E-01
LEP		x	1.17E-01
SPARC		x	1.21E-01
SRXN1	x		1.23E-01
PDGFB		x	1.24E-01
KBTBD8		x	1.24E-01
CBX2		x	1.24E-01
IFNG		x	1.25E-01
TASOR	x		1.27E-01
G6PC		x	1.30E-01
OXTR		x	1.31E-01
PPIH	x		1.32E-01
ZFP282		x	1.32E-01
ADAMTS1	x		1.34E-01
ENTPD7		x	1.35E-01
ICOS		x	1.36E-01
SUV420H1		x	1.37E-01
CRISPLD1		x	1.38E-01
LIMS1		x	1.39E-01
PDGFRB		x	1.39E-01
NDST1		x	1.40E-01
AK3		x	1.40E-01
COL5A2		x	1.40E-01
TGFB2	x		1.40E-01
PIGT		x	1.42E-01
CEP68		x	1.42E-01
ELN		x	1.43E-01
RNF169		x	1.44E-01
C1orf109	x		1.45E-01
ZFP36L1		x	1.45E-01
COMMD8	x		1.47E-01
ARHGAP5		x	1.48E-01
SLC43A2		x	1.49E-01
ARRDC3		x	1.49E-01
HECW1		x	1.53E-01
NAV3		x	1.54E-01
COL3A1		x	1.56E-01
ADAM11	x		1.56E-01
HOXC13		x	1.58E-01
THSD4		x	1.61E-01
TTC5	x		1.61E-01
C2orf80	x		1.63E-01

ADCYAP1R1		x	1.64E-01
HSPG2		x	1.64E-01
SS18L1		x	1.64E-01
HEXA		x	1.65E-01
ULK1	x		1.65E-01
SOAT1	x		1.65E-01
DMWD	x		1.67E-01
CLMN		x	1.67E-01
TSPAN14		x	1.67E-01
TIGD3	x		1.70E-01
IQCG	x		1.72E-01
ARPC4	x		1.73E-01
SLCO5A1		x	1.74E-01
COL8A1		x	1.74E-01
ZHX1		x	1.75E-01
ZFP91		x	1.75E-01
XBP1	x		1.76E-01
EDC3		x	1.78E-01
GAB1		x	1.79E-01
LRRN4CL	x		1.80E-01
ZER1		x	1.80E-01
TDG		x	1.86E-01
MAB21L1	x		1.88E-01
BACE1		x	1.90E-01
TRMT10A	x		1.91E-01
NKIRAS2		x	1.92E-01
ISG20L2	x		1.93E-01
ZBTB40		x	1.93E-01
HNRNPUL1		x	1.99E-01
RANBP3L	x		2.03E-01
SLC39A1	x		2.04E-01
ZMAT3	x		2.05E-01
SLC17A7	x		2.07E-01
POLE3		x	2.08E-01
SCN3B		x	2.08E-01
KPNA4		x	2.09E-01
YPEL2		x	2.14E-01
GLIS2		x	2.15E-01
EIF4A2	x	x	2.15E-01
CX3CL1		x	2.15E-01
PHF21A		x	2.22E-01
CDC42		x	2.27E-01
PITPNA		x	2.27E-01
GCC2		x	2.28E-01
ZFHX2	x		2.28E-01
ZFP191		x	2.29E-01
ZNF195	x		2.35E-01
COL19A1		x	2.35E-01
RAB6B		x	2.36E-01
PHLDB3		x	2.38E-01

SENP1		x	2.39E-01
ZNF830	x		2.41E-01
HP1BP3	x		2.42E-01
OSBPL8	x		2.46E-01
RIC8		x	2.47E-01
CHRM1	x		2.47E-01
TNFRSF1A		x	2.47E-01
TMEM183A		x	2.49E-01
TRAPPC13	x		2.50E-01
ARL4A		x	2.56E-01
PTX3		x	2.58E-01
SMCR7L		x	2.59E-01
USP42		x	2.61E-01
CSGALNACT2		x	2.66E-01
SUV420H2		x	2.66E-01
DRD1A		x	2.67E-01
PLK3	x		2.70E-01
KDM2A		x	2.71E-01
EBNA1BP2	x		2.71E-01
CWC25	x		2.72E-01
ADAMTS9		x	2.72E-01
PARG		x	2.73E-01
NRBP1		x	2.76E-01
KLHL28		x	2.77E-01
ING4		x	2.79E-01
ADAM19		x	2.79E-01
FMC1-LUC7L2	x		2.80E-01
R3HDM1	x		2.83E-01
CCDC117		x	2.83E-01
NFATC3		x	2.87E-01
SLC30A10		x	2.88E-01
RINL	x		2.90E-01
FBN1		x	2.92E-01
MAN2A2	x		2.97E-01
RNF19A		x	2.98E-01
YTHDF1		x	3.00E-01
NMRK2	x		3.04E-01
TSTD2	x		3.06E-01
XRCC2	x		3.13E-01
IFNGR2	x		3.13E-01
HTR7		x	3.19E-01
SLC27A1	x		3.19E-01
SOCS7		x	3.20E-01
SESTD1	x	x	3.31E-01
GRIP1		x	3.31E-01
HCN1		x	3.38E-01
PRKRA		x	3.39E-01
ATP6V1A		x	3.40E-01
SYNPO	x		3.46E-01
ERP29		x	3.47E-01

MYBL2		x	3.49E-01
FZD5	x	x	3.50E-01
DOLPP1		x	3.50E-01
EMP1		x	3.52E-01
DDX1		x	3.52E-01
ELMO2		x	3.54E-01
PTPRF	x	x	3.55E-01
TRIM63		x	3.56E-01
CDK6		x	3.61E-01
SLC7A1		x	3.63E-01
RICTOR		x	3.63E-01
RNF165		x	3.68E-01
CCND2		x	3.69E-01
ERCC6	x	x	3.70E-01
NOSIP	x		3.72E-01
PALM		x	3.74E-01
POGLUT3	x		3.79E-01
ARPP19		x	3.80E-01
KDM4A		x	3.81E-01
KLF4		x	3.87E-01
CCDC88A		x	3.87E-01
XKR7		x	3.89E-01
FBN2		x	3.96E-01
TXNL1	x		3.96E-01
SFPQ		x	3.96E-01
SRF	x		3.99E-01
ZHX3		x	4.03E-01
PALM2		x	4.03E-01
KDR	x		4.03E-01
AQP7	x		4.07E-01
TMX4	x		4.09E-01
OXR1		x	4.10E-01
SLC35E1	x		4.12E-01
MTF1	x		4.12E-01
CCDC80		x	4.17E-01
ATP1B1		x	4.17E-01
TUBE1	x		4.23E-01
PPP2CA		x	4.26E-01
ATXN1L	x		4.38E-01
THAP2	x		4.41E-01
FBXL20		x	4.47E-01
DNMT3B		x	4.47E-01
SC5D	x		4.52E-01
ING3	x		4.53E-01
SLC8B1	x		4.54E-01
PPP2R2A	x		4.61E-01
GAA	x		4.64E-01
TRAFD1		x	4.64E-01
SLC23A2	x		4.70E-01
SLC9A1	x		4.71E-01

FAM131B		x	4.73E-01
RAD1	x		4.76E-01
CALM3		x	4.78E-01
TCFAP2C		x	4.81E-01
SRCRB4D		x	4.83E-01
CALU		x	4.84E-01
ACER1	x		4.85E-01
RNF214		x	4.92E-01
DISP3	x		4.98E-01
AKAP8	x		5.00E-01
MAP3K7	x		5.01E-01
INPP5E	x		5.02E-01
RBFOX2		x	5.03E-01
KCTD20		x	5.07E-01
HDGF		x	5.10E-01
PRX	x		5.18E-01
LDLRAP1		x	5.18E-01
LTBR		x	5.21E-01
TMEM65		x	5.24E-01
CACNA1G	x		5.29E-01
GTPBP2		x	5.30E-01
ZNF354A	x		5.35E-01
ING2	x	x	5.37E-01
LATS1	x		5.39E-01
PDIK1L		x	5.39E-01
RSAD1		x	5.45E-01
COL5A1		x	5.45E-01
COL22A1		x	5.48E-01
PDGFC		x	5.49E-01
PDGFRA		x	5.51E-01
BAHD1		x	5.52E-01
INSRR		x	5.54E-01
KCNC3		x	5.56E-01
TMEM132A		x	5.57E-01
COL9A1		x	5.57E-01
UBFD1		x	5.57E-01
PVRL1		x	5.62E-01
KCTD21		x	5.70E-01
TAF5L	x		5.72E-01
ACTR3	x		5.75E-01
TMEM48		x	5.77E-01
AP4E1		x	5.79E-01
DHX37	x		5.80E-01
TMEM135		x	5.81E-01
PITPNB		x	5.91E-01
RBAK	x		5.95E-01
PDXK	x		5.97E-01
MYCN		x	6.04E-01
NFATC4		x	6.12E-01
CHIC2		x	6.12E-01

SLC30A3		x	6.14E-01
ACTL6A	x		6.16E-01
TET2		x	6.18E-01
TPM1		x	6.20E-01
SH3GLB1		x	6.26E-01
RHOG	x		6.28E-01
PAIP2		x	6.30E-01
MLEC	x		6.32E-01
ZNF558	x		6.32E-01
AJUBA	x		6.33E-01
MMP2		x	6.34E-01
H4C9	x		6.40E-01
COL4A1		x	6.41E-01
UBTD2		x	6.41E-01
TFCP2	x		6.44E-01
NRGN	x		6.45E-01
MZT1	x		6.48E-01
PRPF40A		x	6.49E-01
COL24A1		x	6.50E-01
TCEB1	x		6.51E-01
GATA5	x		6.56E-01
IRGM	x		6.57E-01
MAP2K4		x	6.60E-01
TAF5		x	6.61E-01
MED26		x	6.61E-01
NOP9	x		6.64E-01
UNC13B		x	6.74E-01
NKIRAS1	x		6.75E-01
ADAMTS16		x	6.78E-01
TTC14		x	6.84E-01
ZNF444	x		6.84E-01
HMCN1		x	6.86E-01
PKDCC	x		6.88E-01
SNX13	x		6.90E-01
VPS25		x	6.92E-01
TTC26	x		6.93E-01
GCNT3	x		6.97E-01
MAPK10		x	7.06E-01
HMGN3		x	7.06E-01
FAM136A		x	7.21E-01
OSBP		x	7.23E-01
RNF138		x	7.24E-01
INA		x	7.24E-01
PLIN3	x		7.27E-01
JOSD1		x	7.27E-01
COL6A3		x	7.29E-01
ARRDC4		x	7.40E-01
COL5A3		x	7.46E-01
INSIG1		x	7.48E-01
DUSP22		x	7.53E-01

TGFBR1	x		7.53E-01
ATAD2	x		7.55E-01
KCMF1	x		7.58E-01
PLEKHH2	x		7.62E-01
CFL2		x	7.70E-01
IFFO1		x	7.73E-01
SMTNL2	x	x	7.82E-01
ZNF845	x		7.90E-01
ACAP3		x	8.09E-01
GCSH		x	8.15E-01
FAM76B		x	8.31E-01
CLDN1		x	8.34E-01
ISLR2		x	8.40E-01
HAS2		x	8.51E-01
CDC7		x	8.57E-01
DGKD		x	8.63E-01
SLC25A36		x	8.67E-01
BPNT1	x		8.70E-01
UBE2K		x	8.75E-01
HTR5A	x		8.81E-01
GCAP14		x	8.90E-01
SCO1	x		8.92E-01
LRRC58	x		8.93E-01
DUSP3	x		8.93E-01
GPCPD1	x	x	8.98E-01
RRP36	x		8.98E-01
MARCHF2	x		9.01E-01
KLHDC3		x	9.01E-01
EPHB3		x	9.34E-01
LASP1	x		9.36E-01
ACOT11	x		9.51E-01
RMI2	x		9.53E-01
GJD2		x	9.56E-01
1110018G07RIK	x		#N/D
2310046O06RIK		x	#N/D
2400001E08RIK		x	#N/D
2410066E13RIK	x		#N/D
2810046L04RIK		x	#N/D
3110035E14RIK	x		#N/D
4833424O15RIK		x	#N/D
4921524J17RIK		x	#N/D
4921536K21Rik		x	#N/D
4931406P16RIK		x	#N/D
4931428F04RIK		x	#N/D
4933439F18RIK		x	#N/D
5133401N09RIK	x		#N/D
6720456H20RIK		x	#N/D
8030411F24RIK		x	#N/D
8430427H17RIK		x	#N/D
9130011E15RIK	x		#N/D

9530068E07RIK		x	#N/D
AC008397.2	x		#N/D
AC093525.2	x		#N/D
AGTR2		x	#N/D
AMOT	x	x	#N/D
AP002748.5	x		#N/D
ARF2		x	#N/D
ARHGAP36		x	#N/D
ASB11		x	#N/D
ATP1B4		x	#N/D
ATRX	x	x	#N/D
AU019823		x	#N/D
B230219D22RIK		x	#N/D
BC023814	x		#N/D
BC035295		x	#N/D
BC060632		x	#N/D
BC065085		x	#N/D
BRWD3		x	#N/D
C030046E11RIK		x	#N/D
C030046I01RIK		x	#N/D
C330019G07RIK		x	#N/D
C6orf47	x		#N/D
C77370		x	#N/D
CASTOR2	x		#N/D
CCDC28B		x	#N/D
CCL15-CCL14	x		#N/D
CCNL2		x	#N/D
CDK16	x		#N/D
CENPB		x	#N/D
COL4A5		x	#N/D
COL4A6		x	#N/D
CSDA		x	#N/D
CST6	x		#N/D
D0H4S114		x	#N/D
D19WSU162E		x	#N/D
D3BWG0562E		x	#N/D
D4ERTD22E	x		#N/D
D630045J12RIK		x	#N/D
DCX		x	#N/D
DDX3X		x	#N/D
DGKK		x	#N/D
DYNLT1B		x	#N/D
EFNB1	x		#N/D
ELOVL1	x		#N/D
EN1		x	#N/D
ENO2	x		#N/D
EOMES		x	#N/D
EPB4.1L4B		x	#N/D
ERLIN2		x	#N/D
EXOSC4	x		#N/D

FAM123B		x	#N/D
FAM70A		x	#N/D
FBXW9		x	#N/D
FOS		x	#N/D
FOXJ2		x	#N/D
FOXL1	x		#N/D
FOXP3	x		#N/D
FRAT2		x	#N/D
GDF3	x		#N/D
GLA	x		#N/D
GM11818	x		#N/D
Gm14137		x	#N/D
GM1587	x		#N/D
GM749	x		#N/D
GNL3L	x		#N/D
HDAC8	x		#N/D
HNRNPCL1	x		#N/D
HOXA1	x		#N/D
HTR2C		x	#N/D
IGSF1		x	#N/D
ING5	x		#N/D
KDM5C	x	x	#N/D
KIFC2		x	#N/D
MAFB		x	#N/D
MAFG		x	#N/D
MAZ		x	#N/D
MORF4L2		x	#N/D
NAP1L3		x	#N/D
NHSL2	x		#N/D
NKRF		x	#N/D
NLGN3	x	x	#N/D
NNAT	x		#N/D
NPAS4		x	#N/D
NTF4	x		#N/D
NUDT11		x	#N/D
NXT2	x		#N/D
ORF61		x	#N/D
P2RY4	x		#N/D
PCYT1B		x	#N/D
PDGFA		x	#N/D
PEX5		x	#N/D
PHC1		x	#N/D
PIGV	x		#N/D
PLP1		x	#N/D
POU3F1	x		#N/D
PPM1D		x	#N/D
PPP1R3D		x	#N/D
PRR3	x	x	#N/D
PYCR1	x		#N/D
RBM34	x		#N/D

REPS2		x	#N/D
RLIM		x	#N/D
SCML2		x	#N/D
SCRT1	x		#N/D
SEMA4C	x		#N/D
SETD8		x	#N/D
SH3RF3		x	#N/D
SHH	x		#N/D
SHROOM2		x	#N/D
SLC16A2		x	#N/D
SLC25A52	x		#N/D
SLC35E2B	x		#N/D
SMS		x	#N/D
SOCS1		x	#N/D
SPNA2		x	#N/D
SPNB2		x	#N/D
SRGAP2		x	#N/D
STAG2		x	#N/D
STARD8		x	#N/D
TCEANC	x		#N/D
TCFAP4	x		#N/D
THOC2	x	x	#N/D
TMEM55B	x	x	#N/D
TRIM27	x		#N/D
TRP53INP1		x	#N/D
TRP53INP2	x	x	#N/D
TSC22D3		x	#N/D
TUBB	x		#N/D
TUBB2B		x	#N/D
XKRX	x		#N/D
ZFP36		x	#N/D
ZFP366		x	#N/D
ZFP384		x	#N/D
ZFP385C		x	#N/D
ZFP532		x	#N/D
ZFP629	x		#N/D
ZFP641		x	#N/D
ZFP652		x	#N/D
ZFP704		x	#N/D
ZFP827		x	#N/D

In bold significant results after Bonferroni multiple testing correction
Yengo et al 2018. Hum Mol Genet. doi: 10.1093/hmg/ddy271.

Table S6. Significantly enriched KEGG pathways from the miR-29c-3p target genes

GeneSet	Description	Size	Overlap	Expect	Ratio	pValue	FDR	Genes (Enterz ID)
hsa04974	Protein digestion and absorption	90	25	3.30	7.56	6.66E-16	2.17E-13	1278;1280;1281;1282;1284;1285;1286;1287;1288;1289;1290;1293;1294;1297;1301;1306;169044;1803;2006;23439;255631;481;50509;54407;85301
hsa04510	Focal adhesion	199	34	7.31	4.65	2.41E-14	3.93E-12	10000;1278;1280;1282;1284;1285;1286;1287;1288;1293;1297;22801;2932;3381;3479;3688;3915;394;5154;5155;5156;5159;5295;5500;5602;56034;5728;6714;7414;7422;8503;858;894;998
hsa04151	PI3K-Akt signaling pathway	354	41	13.00	3.15	2.83E-11	3.08E-09	10000;1021;1026;1278;1280;1282;1284;1285;1286;1287;1288;1293;1297;1946;22801;2309;2538;2932;3381;3479;3688;3915;4170;4893;5154;5155;5156;5159;5295;54331;5515;55970;56034;5728;6446;7422;7531;8503;894;9470;9586
hsa04926	Relaxin signaling pathway	130	22	4.77	4.61	1.40E-09	1.14E-07	10000;1278;1281;1282;1284;1285;1286;1287;1288;1910;2353;4313;4893;5295;54331;55970;5602;6416;6714;7422;8503;9586
hsa05200	Pathways in cancer	524	48	19.24	2.49	1.91E-09	1.24E-07	10000;1021;1026;1282;1284;1285;1286;1287;1288;1910;23401;2353;27113;27436;2932;3458;3479;3688;3915;405;4313;4893;5154;5155;5156;5159;5295;54331;5467;55970;5602;5728;578;5898;5915;5979;6667;7043;7187;7422;7855;8031;808;818;8503;894;9618;998
hsa01521	EGFR tyrosine kinase inhibitor resistance	79	17	2.90	5.86	2.49E-09	1.36E-07	10000;2309;2549;2932;3479;4893;5154;5155;5156;5159;5295;56034;5728;6714;7422;8503;9470
hsa05222	Small cell lung cancer	92	18	3.38	5.33	4.25E-09	1.98E-07	10000;1021;1026;1282;1284;1285;1286;1287;1288;3688;3915;5295;5728;578;5915;7187;8503;9618
hsa05231	Choline metabolism in cancer	99	18	3.64	4.95	1.44E-08	5.88E-07	10000;139189;160851;2353;4893;5154;5155;5156;5159;5295;5602;56034;56261;6584;6667;8503;8527;9468
hsa05165	Human papillomavirus infection	339	35	12.45	2.81	1.99E-08	7.16E-07	10000;1021;1026;1278;1280;1282;1284;1285;1286;1287;1288;1293;1297;1740;22801;2932;3381;3688;3915;4893;5159;523;5295;5515;5728;578;7132;7187;7422;7855;8503;894;9586;9759;998
hsa05206	MicroRNAs in cancer	150	22	5.51	3.99	2.20E-08	7.16E-07	1021;1026;1788;1789;23405;23411;4170;4893;5154;5155;5156;5159;5728;578;599;6541;7168;7422;8651;894;90427;9759
hsa05214	Glioma	71	15	2.61	5.75	2.90E-08	8.60E-07	10000;1021;1026;3479;4893;5154;5155;5156;5159;5295;5728;578;808;818;8503
hsa04512	ECM-receptor interaction	82	16	3.01	5.31	3.30E-08	8.96E-07	1278;1280;1282;1284;1285;1286;1287;1288;1293;1297;1605;22801;3339;3381;3688;3915
hsa04933	AGE-RAGE signaling pathway in diabetic complications	99	17	3.64	4.68	8.88E-08	2.23E-06	10000;1278;1281;1282;1284;1285;1286;1287;1288;4313;4893;5295;5602;7043;7422;8503;998
hsa05218	Melanoma	72	14	2.64	5.30	2.55E-07	5.94E-06	10000;1021;1026;3479;4893;5154;5155;5156;5159;5295;56034;5728;578;8503
hsa05167	Kaposi sarcoma-associated herpesvirus infection	186	22	6.83	3.22	1.05E-06	2.28E-05	10000;1021;1026;2353;2932;4775;4776;4893;5155;5295;54331;55970;5602;578;6416;6714;7132;7187;7422;7538;808;8503
hsa05215	Prostate cancer	97	15	3.56	4.21	2.08E-06	4.24E-05	10000;1026;2932;3479;3645;4893;5154;5155;5156;5159;5295;56034;5728;8503;9586

hsa05205	Proteoglycans in cancer	198	22	7.27	3.03	3.03E-06	5.81E-05	10000;1026;1839;2549;286;288;3339;3479;3688;4313;4893;5295;5500;5781;6714;7074;7422;7855;818;8503;858;998
hsa05211	Renal cell carcinoma	69	12	2.53	4.74	6.39E-06	1.16E-04	10000;1026;2549;405;4893;5155;5295;5781;7043;7422;8503;998
hsa04917	Prolactin signaling pathway	70	12	2.57	4.67	7.46E-06	1.28E-04	10000;2309;2353;2932;30837;4893;5295;5602;6714;8503;8651;894
hsa05146	Amoebiasis	96	14	3.53	3.97	9.26E-06	1.51E-04	1278;1281;1282;1284;1285;1286;1287;1288;3458;3915;5295;7043;7414;8503
hsa05161	Hepatitis B	144	17	5.29	3.21	1.88E-05	2.92E-04	10000;1021;1026;1654;2353;3339;4775;4776;4893;5295;5602;5728;6416;6714;7043;8503;9586
hsa04360	Axon guidance	175	19	6.43	2.96	2.03E-05	3.01E-04	1073;1808;1946;2049;23380;27289;2932;3688;4775;4776;4893;5295;56896;5781;6091;6714;818;8503;998
hsa04014	Ras signaling pathway	232	22	8.52	2.58	3.85E-05	5.46E-04	10000;1946;2549;3363;3479;4893;5154;5155;5156;5159;5295;54331;55970;5602;56034;5781;5898;7074;7422;808;8503;998
hsa05418	Fluid shear stress and atherosclerosis	138	16	5.07	3.16	4.15E-05	5.64E-04	10000;2353;3458;4313;5154;5155;5295;5602;6416;657;6714;7132;7422;808;8503;858
hsa05166	Human T-cell leukemia virus 1 infection	255	23	9.36	2.46	5.64E-05	7.08E-04	10000;1026;2353;2932;4055;4605;4775;4776;4893;5154;5155;5156;5159;5295;54107;6416;7043;7132;7538;7855;821;8503;894
hsa04012	ErbB signaling pathway	85	12	3.12	3.84	5.65E-05	7.08E-04	10000;1026;1839;2549;2932;4893;5295;5602;6416;6714;818;8503
hsa04015	Rap1 signaling pathway	206	20	7.56	2.64	6.26E-05	7.39E-04	10000;1268;1500;1946;3479;3688;4893;5154;5155;5156;5159;5295;56034;5898;6714;7074;7422;808;8503;998
hsa05210	Colorectal cancer	86	12	3.16	3.80	6.35E-05	7.39E-04	10000;1026;2353;27113;2932;4893;5295;5602;578;5898;7043;8503
hsa04218	Cellular senescence	160	17	5.88	2.89	7.35E-05	8.27E-04	10000;1021;1026;2309;23411;4605;4775;4776;4893;5295;5500;5728;677;7043;808;8503;894
hsa04540	Gap junction	88	12	3.23	3.71	7.99E-05	8.43E-04	3358;347733;4893;5154;5155;5156;5159;5592;56034;57369;6714;7280
hsa05212	Pancreatic cancer	75	11	2.75	3.99	8.08E-05	8.43E-04	10000;1021;1026;5295;5602;578;5898;7043;7422;8503;998
hsa04072	Phospholipase D signaling pathway	146	16	5.36	2.98	8.27E-05	8.43E-04	10000;139189;160851;2549;26052;4893;5154;5155;5156;5159;5295;56034;5781;5898;8503;8527
hsa05223	Non-small cell lung cancer	66	10	2.42	4.13	1.29E-04	0.001274	10000;1021;1026;2309;27436;4893;5295;578;5915;8503
hsa05163	Human cytomegalovirus infection	225	20	8.26	2.42	2.12E-04	0.00203	10000;1021;1026;2932;4775;4776;4893;5156;5295;54331;55970;578;6376;6667;6714;7132;7422;808;8503;9586
hsa01522	Endocrine resistance	98	12	3.60	3.33	2.28E-04	0.002108	10000;1026;1839;2353;3479;4313;4893;5295;5602;6667;6714;8503
hsa05213	Endometrial cancer	58	9	2.13	4.23	2.33E-04	0.002108	10000;1026;2309;2932;4893;5295;5728;578;8503
hsa04630	JAK-STAT signaling pathway	162	16	5.95	2.69	2.81E-04	0.002477	10000;1026;30837;3458;3952;3976;4170;5154;5155;5156;5159;5295;5781;8503;8651;894
hsa04810	Regulation of actin cytoskeleton	213	19	7.82	2.43	2.91E-04	0.0025	10458;1073;22801;3645;3688;4893;5154;5155;5156;5159;5295;5500;55970;56034;6714;7074;7414;8503;998

hsa04068	FoxO signaling pathway	132	14	4.85	2.89	3.24E-04	0.002711	10000;1026;2309;23411;2538;3479;4893;5295;5602;5728;6446;7043;8503;894
hsa05100	Bacterial invasion of epithelial cells	74	10	2.72	3.68	3.39E-04	0.002764	2549;26052;3688;5295;63916;6714;7414;8503;858;998
hsa04722	Neurotrophin signaling pathway	119	13	4.37	2.97	3.96E-04	0.003148	10000;2309;2549;2932;4893;5295;5602;5781;7531;808;818;8503;998
hsa04931	Insulin resistance	107	12	3.93	3.05	5.19E-04	0.004027	10000;2538;2932;5295;5500;5509;5602;5728;5781;7132;8503;9586
hsa04550	Signaling pathways regulating pluripotency of stem cells	139	14	5.10	2.74	5.52E-04	0.004182	10000;10336;2932;3479;3670;3720;3976;4893;5295;657;7855;8503;9314;9869
hsa04668	TNF signaling pathway	110	12	4.04	2.97	6.68E-04	0.004947	10000;153090;2353;3976;5295;5602;6376;6416;7132;7187;8503;9586
hsa04215	Apoptosis	32	6	1.18	5.11	9.39E-04	0.006799	27113;5602;56616;57448;578;7132
hsa05224	Breast cancer	147	14	5.40	2.59	9.65E-04	0.006838	10000;1021;1026;23401;2353;2932;3479;4893;5295;5728;578;6667;7855;8503
hsa04066	HIF-1 signaling pathway	100	11	3.67	3.00	0.001038	0.007198	10000;1026;3458;3479;405;4055;5295;7422;818;8503;9470
hsa04152	AMPK signaling pathway	120	12	4.41	2.72	0.001446	0.00982	10000;2309;23411;2538;3156;3479;3952;51552;5295;5515;8503;9586
hsa05220	Chronic myeloid leukemia	76	9	2.79	3.22	0.001751	0.011651	10000;1021;1026;4893;5295;578;5781;7043;8503
hsa04960	Aldosterone-regulated sodium reabsorption	37	6	1.36	4.42	0.002065	0.013462	23439;3479;481;5295;6446;8503
hsa05230	Central carbon metabolism in cancer	65	8	2.39	3.35	0.002445	0.015629	10000;4893;5156;5159;5295;5728;5979;8503
hsa04670	Leukocyte transendothelial migration	112	11	4.11	2.67	0.002609	0.016358	1500;3688;394;4313;50848;5295;5781;7414;8503;9076;998
hsa04010	MAPK signaling pathway	295	21	10.83	1.94	0.002685	0.016514	10000;1844;1946;2353;3479;3556;4775;4893;5154;5155;5156;5159;55970;5602;56034;6416;7043;7132;7422;9448;998
hsa05225	Hepatocellular carcinoma	167	14	6.13	2.28	0.003238	0.019162	10000;1021;1026;23401;2549;2932;4893;5295;5728;578;6599;7043;7855;8503
hsa05120	Epithelial cell signaling in Helicobacter pylori infection	68	8	2.50	3.20	0.003258	0.019162	1839;50848;523;5602;5781;6416;6714;998
hsa04750	Inflammatory mediator regulation of TRP channels	99	10	3.64	2.75	0.003292	0.019162	3358;3479;3556;5295;5500;5602;6714;808;818;8503

hsa05219	Bladder cancer	41	6	1.51	3.99	0.003533	0.020093	1026;1839;4313;4893;6714;7422
hsa04150	mTOR signaling pathway	151	13	5.54	2.34	0.003575	0.020093	10000;253260;2932;3479;4893;523;5295;5728;6446;7132;7855;8503;9470
hsa04660	T cell receptor signaling pathway	101	10	3.71	2.70	0.003807	0.021038	10000;2353;2932;29851;3458;4775;4893;5295;8503;998
hsa05142	Chagas disease (American trypanosomiasis)	102	10	3.75	2.67	0.004088	0.022125	10000;2353;3458;5295;5515;5602;6416;7043;7132;8503
hsa04210	Apoptosis	136	12	4.99	2.40	0.00414	0.022125	10000;153090;2353;27113;4170;4893;5295;5602;56616;578;7132;8503
hsa04910	Insulin signaling pathway	137	12	5.03	2.39	0.004393	0.023099	10000;2538;2932;4893;5295;5500;5509;5602;808;8503;8651;9470
hsa04625	C-type lectin receptor signaling pathway	104	10	3.82	2.62	0.004699	0.024316	10000;4775;4776;4893;5295;5602;5781;6714;808;8503
hsa04973	Carbohydrate digestion and absorption	44	6	1.62	3.71	0.00506	0.025385	10000;23439;2538;481;5295;8503
hsa01524	Platinum drug resistance	73	8	2.68	2.98	0.005062	0.025385	10000;1026;1317;27113;5295;578;5980;8503
hsa04211	Longevity regulating pathway	89	9	3.27	2.75	0.005167	0.025523	10000;2309;23411;3479;4893;5295;8503;9470;9586
hsa04370	VEGF signaling pathway	59	7	2.17	3.23	0.005545	0.026982	10000;4893;5295;6714;7422;8503;998
hsa04024	cAMP signaling pathway	199	15	7.31	2.05	0.006355	0.030467	10000;117;23439;2353;481;493;5021;5295;5500;5602;7074;808;818;8503;9586
hsa04912	GnRH signaling pathway	93	9	3.42	2.64	0.006888	0.032391	1839;4313;4893;5602;6416;6714;808;818;998
hsa05203	Viral carcinogenesis	201	15	7.38	2.03	0.006955	0.032391	1021;1026;1654;4055;4893;5295;578;6714;7187;7531;8503;894;9586;9759;998
hsa04140	Autophagy	128	11	4.70	2.34	0.007229	0.03253	10000;4893;51100;5295;55014;5515;5602;5728;79065;8503;9110
hsa04310	Wnt signaling pathway	146	12	5.36	2.24	0.007272	0.03253	23002;23401;23500;2932;4775;4776;5467;5602;56998;7855;818;894
hsa04213	Longevity regulating pathway	62	7	2.28	3.07	0.007284	0.03253	10000;2309;23411;3479;4893;5295;8503
hsa05226	Gastric cancer	148	12	5.43	2.21	0.008079	0.035589	10000;1026;23401;2549;2932;4893;5295;578;5915;7043;7855;8503
hsa04728	Dopaminergic synapse	131	11	4.81	2.29	0.008559	0.036714	10000;2353;2932;54331;5500;5515;55970;5602;808;818;9586
hsa05160	Hepatitis C	131	11	4.81	2.29	0.008559	0.036714	10000;1026;2932;4893;5295;5515;5602;7132;7187;8503;9076
hsa04919	Thyroid hormone signaling pathway	116	10	4.26	2.35	0.010013	0.042392	10000;1734;23439;2932;481;4893;5295;6567;6714;8503

hsa04070	Phosphatidylinositol signaling system	99	9	3.64	2.48	0.010253	0.042854	139189;160851;23262;5295;5728;808;8503;8527;9110
hsa05216	Thyroid cancer	37	5	1.36	3.68	0.010709	0.044191	1026;4893;578;5979;8031
hsa05170	Human immunodeficiency virus 1 infection	212	15	7.78	1.93	0.011115	0.045294	10000;1073;164;2353;4775;4776;4893;5295;54331;55970;5602;578;7132;808;8503
hsa04915	Estrogen signaling pathway	137	11	5.03	2.19	0.011784	0.047423	10000;1839;2353;4313;4893;5295;6667;6714;808;8503;9586
hsa05031	Amphetamine addiction	68	7	2.50	2.80	0.011928	0.047423	23411;2353;5500;6804;808;818;9586

Table S7. Significantly enriched KEGG pathways from the differentially expressed target genes of miR-29c-3p.

Geneset	Description	Size	Overlap	Expect	Enrichment ratio	Pvalue	FDR	Overlapid
hsa04974	Protein digestion and absorption	90	9	0.73	12.32	3.44E-08	1.12E-05	1278;1281;1285;1287;1288;1289;1290;2006;54407
hsa04510	Focal adhesion	199	11	1.61	6.81	4.34E-07	6.86E-05	1278;1285;1287;1288;3688;3915;5602;5728;7414;8503;894
hsa05222	Small cell lung cancer	92	8	0.75	10.72	6.31E-07	6.86E-05	1021;1285;1287;1288;3688;3915;5728;8503
hsa05146	Amoebiasis	96	8	0.78	10.27	8.77E-07	7.15E-05	1278;1281;1285;1287;1288;3915;7414;8503
hsa04151	PI3K-Akt signaling pathway	354	13	2.87	4.53	3.65E-06	2.07E-04	1021;1278;1285;1287;1288;1946;3688;3915;55970;5728;6446;8503;894
hsa04512	ECM-receptor interaction	82	7	0.67	10.52	3.81E-06	2.07E-04	1278;1285;1287;1288;3339;3688;3915
hsa04926	Relaxin signaling pathway	130	8	1.05	7.58	8.68E-06	4.04E-04	1278;1281;1285;1287;1288;55970;5602;8503
hsa04933	AGE-RAGE signaling pathway in diabetic complications	99	7	0.80	8.71	1.34E-05	5.46E-04	1278;1281;1285;1287;1288;5602;8503
hsa04218	Cellular senescence	160	8	1.30	6.16	3.94E-05	0.00142865	1021;4605;4775;4776;5728;677;8503;894
hsa05200	Pathways in cancer	524	14	4.25	3.29	5.53E-05	0.0018017	1021;1285;1287;1288;3688;3915;55970;5602;5728;5898;6667;8031;8503;894
hsa05165	Human papillomavirus infection	339	11	2.75	4.00	7.18E-05	0.0021291	1021;1278;1285;1287;1288;3688;3915;5728;7132;8503;894
hsa05167	Kaposi sarcoma-associated herpesvirus infection	186	8	1.51	5.30	1.15E-04	0.00311137	1021;4775;4776;55970;5602;7132;7538;8503
hsa05161	Hepatitis B	144	7	1.17	5.99	1.49E-04	0.00373505	1021;3339;4775;4776;5602;5728;8503
hsa05166	Human T-cell leukemia virus 1 infection	255	8	2.07	3.87	9.65E-04	0.0224594	4055;4605;4775;4776;7132;7538;8503;894
hsa04310	Wnt signaling pathway	146	6	1.18	5.06	0.00110967	0.02410388	23002;23500;4775;4776;5602;894
hsa05231	Choline metabolism in cancer	99	5	0.80	6.22	0.00118301	0.02410388	5602;56261;6584;6667;8503
hsa04625	C-type lectin receptor signaling pathway	104	5	0.84	5.92	0.0014749	0.02765645	4775;4776;5602;5781;8503
hsa05170	Human immunodeficiency virus 1 infection	212	7	1.72	4.07	0.00152704	0.02765645	164;4775;4776;55970;5602;7132;8503
hsa04931	Insulin resistance	107	5	0.87	5.76	0.00167373	0.02871775	5602;5728;5781;7132;8503
hsa05163	Human cytomegalovirus infection	225	7	1.83	3.83	0.00214685	0.03499371	1021;4775;4776;55970;6667;7132;8503
hsa04014	Ras signaling pathway	232	7	1.88	3.72	0.00255311	0.03963392	1946;2549;55970;5602;5781;5898;8503
hsa04360	Axon guidance	175	6	1.42	4.23	0.00279394	0.04140116	1946;3688;4775;4776;5781;8503
hsa05100	Bacterial invasion of epithelial cells	74	4	0.60	6.66	0.00294841	0.04179053	2549;3688;7414;8503
hsa05212	Pancreatic cancer	75	4	0.61	6.57	0.00309587	0.04205219	1021;5602;5898;8503
hsa04140	Autophagy	128	5	1.04	4.81	0.00366009	0.04772759	51100;5602;5728;8503;9110

Table S8. Significantly enriched Reactome pathways from the differentially expressed target genes of miR-29c-3p or miR-665-3p.

miRNA	Geneset	Description	Size	Overlap	Expect	Enrichment Ratio	Pvalue	FDR	Overlapid
miR-29c-3p	R-HSA-1474244	Extracellular matrix organization	301	21	2.48	8.46	2.51E-14	4.34E-11	1278;1281;1285;1287;1288;1289;1290;1295;1307;1310;2006;22795;23473;3339;3688;3915;4237;56999;8038;871;8728
miR-29c-3p	R-HSA-1474228	Degradation of the extracellular matrix	140	15	1.15	13.00	3.79E-13	3.27E-10	1278;1281;1285;1287;1288;1289;1290;1295;1307;1310;2006;23473;3339;3915;56999
miR-29c-3p	R-HSA-8948216	Collagen chain trimerization	44	10	0.36	27.57	1.68E-12	9.70E-10	1278;1281;1285;1287;1288;1289;1290;1295;1307;1310
miR-29c-3p	R-HSA-1650814	Collagen biosynthesis and modifying enzymes	67	11	0.55	19.91	5.51E-12	2.38E-09	1278;1281;1285;1287;1288;1289;1290;1295;1307;1310;871
miR-29c-3p	R-HSA-3000171	Non-integrin membrane-ECM interactions	59	10	0.49	20.56	3.86E-11	1.33E-08	1278;1281;1285;1287;1288;1289;1290;3339;3688;3915
miR-29c-3p	R-HSA-216083	Integrin cell surface interactions	85	11	0.70	15.70	8.11E-11	2.22E-08	1278;1281;1285;1287;1288;1289;1290;1295;1307;3339;3688
miR-29c-3p	R-HSA-1442490	Collagen degradation	64	10	0.53	18.95	8.99E-11	2.22E-08	1278;1281;1285;1287;1288;1289;1290;1295;1307;1310
miR-29c-3p	R-HSA-1474290	Collagen formation	90	11	0.74	14.83	1.53E-10	3.31E-08	1278;1281;1285;1287;1288;1289;1290;1295;1307;1310;871
miR-29c-3p	R-HSA-3000178	ECM proteoglycans	76	10	0.63	15.96	5.23E-10	1.00E-07	1278;1281;1285;1287;1288;1289;1290;3339;3688;3915
miR-29c-3p	R-HSA-3000157	Laminin interactions	30	7	0.25	28.30	3.54E-09	6.11E-07	1285;1287;1288;22795;3339;3688;3915
miR-29c-3p	R-HSA-2022090	Assembly of collagen fibrils and other multimeric structures	61	8	0.50	15.91	3.17E-08	4.99E-06	1278;1281;1285;1287;1288;1289;1290;1295
miR-29c-3p	R-HSA-8875878	MET promotes cell motility	41	7	0.34	20.71	3.63E-08	5.23E-06	1278;1281;1289;1290;2549;3688;3915
miR-29c-3p	R-HSA-8874081	MET activates PTK2 signaling	30	6	0.25	24.26	1.34E-07	1.78E-05	1278;1281;1289;1290;3688;3915
miR-29c-3p	R-HSA-6806834	Signaling by MET	79	8	0.65	12.28	2.49E-07	3.08E-05	1278;1281;1289;1290;2549;3688;3915;5781
miR-29c-3p	R-HSA-3000170	Syndecan interactions	27	5	0.22	22.46	2.37E-06	2.73E-04	1278;1281;1289;1290;3688
miR-29c-3p	R-HSA-2214320	Anchoring fibril formation	15	4	0.12	32.35	5.49E-06	5.93E-04	1278;1285;1287;1288
miR-29c-3p	R-HSA-186797	Signaling by PDGF	58	6	0.48	12.55	7.57E-06	7.70E-04	1281;1285;1287;1289;1290;5781
miR-29c-3p	R-HSA-2243919	Crosslinking of collagen fibrils	18	4	0.15	26.96	1.21E-05	1.16E-03	1278;1285;1287;1288
miR-29c-3p	R-HSA-9006934	Signaling by Receptor Tyrosine Kinases	455	14	3.75	3.73	1.89E-05	1.72E-03	1278;1281;1285;1287;1289;1290;2549;3688;3915;5781;5796;5898;8038;8503
miR-29c-3p	R-HSA-419037	NCAM1 interactions	42	5	0.35	14.44	2.27E-05	1.96E-03	1281;1285;1287;1289;1290
miR-29c-3p	R-HSA-375165	NCAM signaling for neurite out-growth	63	5	0.52	9.63	1.64E-04	0.01346915	1281;1285;1287;1289;1290
miR-29c-3p	R-HSA-8934593	Regulation of RUNX1 Expression and Activity	17	3	0.14	21.41	3.39E-04	0.02659031	1021;5781;894
miR-29c-3p	R-HSA-177929	Signaling by EGFR	43	4	0.35	11.28	4.16E-04	0.03126549	2549;5781;5796;8038
miR-29c-3p	R-HSA-8865999	MET activates PTPN11	5	2	0.04	48.52	6.61E-04	0.04760224	2549;5781
miR-665-3p	R-HSA-556833	Metabolism of lipids	737	13	3.49	3.72E	2.67E-05	0.0461916	10226;11145;114882;166929;26027;26063;2717;4520;56261;6309;63874;64834;9110

Table S9. Significantly enriched GO Biological process from the differentially expressed target genes of miR-29c-3p, miR-665-3p or miR-137-3p

miRNA	Geneset	Description	Size	Overlap	Expect	Enrichment Ratio	Pvalue	FDR	Overlapid
miR-29c-3p	GO:0030198	extracellular matrix organization	347	22	2.71	8.13	2.95E-14	2.68E-10	871;1278;1281;1285;1287;1288;1289;1290;1295;1307;1310;2006;3339;3688;3915;4237;5806;7132;8038;8728;22795;56999
miR-29c-3p	GO:0043062	extracellular structure organization	400	22	3.12	7.05	5.29E-13	2.41E-09	871;1278;1281;1285;1287;1288;1289;1290;1295;1307;1310;2006;3339;3688;3915;4237;5806;7132;8038;8728;22795;56999
miR-29c-3p	GO:0031589	cell-substrate adhesion	332	16	2.59	6.18	6.70E-09	2.03E-05	1021;1281;1295;1307;1946;3688;3915;5728;5796;7414;9341;9448;10979;22795;56999;158326
miR-29c-3p	GO:0007160	cell-matrix adhesion	216	13	1.69	7.71	1.40E-08	3.18E-05	1021;1281;1307;1946;3688;5728;5796;7414;9448;10979;22795;56999;158326
miR-29c-3p	GO:0072359	circulatory system development	1026	25	8.00	3.12	3.09E-07	5.62E-04	657;677;1278;1281;1285;1289;1295;2006;2549;3339;3688;3720;4775;4776;5021;5168;5728;5781;6667;7132;7798;8038;8503; 56999;65009
miR-29c-3p	GO:0007155	cell adhesion	1369	29	10.68	2.72	5.61E-07	8.22E-04	677;1021;1281;1285;1288;1289;1295;1307;1310;1946;3556;3688;3915;5728;5781;5796;7414;8038;8728;9341;9448;10979;227 95;56134;56138;56147;56999;158326;220296
miR-29c-3p	GO:0022610	biological adhesion	1377	29	10.74	2.70	6.33E-07	8.22E-04	677;1021;1281;1285;1288;1289;1295;1307;1310;1946;3556;3688;3915;5728;5781;5796;7414;8038;8728;9341;9448;10979;227 95;56134;56138;56147;56999;158326;220296
miR-29c-3p	GO:0001944	vasculature development	682	19	5.32	3.57	1.41E-06	1.60E-03	657;677;1278;1281;1285;1289;1295;2549;3339;3688;4775;4776;5168;5728;6667;7798;8038;8503;56999
miR-29c-3p	GO:0072358	cardiovascular system development	691	19	5.39	3.52	1.71E-06	1.73E-03	657;677;1278;1281;1285;1289;1295;2549;3339;3688;4775;4776;5168;5728;6667;7798;8038;8503;56999
miR-29c-3p	GO:0001568	blood vessel development	654	18	5.10	3.53	3.25E-06	2.95E-03	657;677;1278;1281;1285;1289;1295;2549;3339;3688;4776;5168;5728;6667;7798;8038;8503;56999
miR-29c-3p	GO:0034333	adherens junction assembly	88	7	0.69	10.20	5.62E-06	4.64E-03	1307;1946;5728;5796;7414;9448;10979
miR-29c-3p	GO:0051093	negative regulation of developmental process	905	21	7.06	2.97	6.70E-06	5.08E-03	657;677;1021;1281;1285;1289;1290;1946;3339;3688;3720;4775;4776;5376;5728;7538;9448;11173;23500;28951;56999
miR-29c-3p	GO:0007044	cell-substrate junction assembly	95	7	0.74	9.45	9.34E-06	6.53E-03	1307;1946;3915;5728;5796;9448;10979
miR-29c-3p	GO:0007507	heart development	536	15	4.18	3.59	1.89E-05	0.01180215	657;677;1281;1289;2006;3688;3720;4776;5021;5728;5781;7132;7798;56999;65009
miR-29c-3p	GO:2000026	regulation of multicellular organismal development	1908	32	14.88	2.15	1.95E-05	0.01180215	657;677;1021;1281;1285;1289;1290;1946;2549;3339;3556;3688;3720;4775;4776;5021;5168;5376;5728;6667;7132;7538;8038; 9448;11173;22849;23002;23072;23500;26039;56999;65009
miR-29c-3p	GO:0045596	negative regulation of cell differentiation	678	17	5.29	3.21	2.09E-05	0.01189067	657;677;1021;1281;1289;1290;1946;3688;4775;4776;5376;5728;7538;9448;11173;23500;28951
miR-29c-3p	GO:0051241	negative regulation of multicellular organismal process	1143	23	8.92	2.58	2.35E-05	0.01216618	657;677;1021;1281;1285;1289;1290;3339;3720;4775;4776;5021;5376;5728;5781;7132;7538;9448;11173;23500;28951;56999;5 7561
miR-29c-3p	GO:0007492	endoderm development	74	6	0.58	10.39	2.41E-05	0.01216618	657;677;1289;1290;1295;3915
miR-29c-3p	GO:0007045	cell-substrate adherens junction assembly	79	6	0.62	9.74	3.50E-05	0.01591511	1307;1946;5728;5796;9448;10979
miR-29c-3p	GO:0048041	focal adhesion assembly	79	6	0.62	9.74	3.50E-05	0.01591511	1307;1946;5728;5796;9448;10979
miR-29c-3p	GO:0030199	collagen fibril organization	49	5	0.38	13.08	3.87E-05	0.01676791	871;1278;1281;1289;1290
miR-29c-3p	GO:0048514	blood vessel morphogenesis	574	15	4.48	3.35	4.16E-05	0.01717549	657;677;1281;1285;1295;2549;3339;3688;4776;5168;5728;6667;8038;8503;56999
miR-29c-3p	GO:0038063	collagen-activated tyrosine kinase receptor signaling pathway	10	3	0.08	38.46	5.35E-05	0.01964372	1285;1287;1288
miR-29c-3p	GO:0061157	mRNA destabilization	27	4	0.21	18.99	5.40E-05	0.01964372	677;7538;22849;253943
miR-29c-3p	GO:0035239	tube morphogenesis	806	18	6.29	2.86	5.40E-05	0.01964372	657;677;1281;1285;1295;2549;3339;3688;4776;5168;5728;5898;6667;7798;8038;8503;56999;65009
miR-29c-3p	GO:0050779	RNA destabilization	29	4	0.23	17.68	7.22E-05	0.02524522	677;7538;22849;253943
miR-29c-3p	GO:0035295	tube development	992	20	7.74	2.58	8.36E-05	0.02814293	657;677;1281;1285;1295;2549;3339;3688;4774;4776;5021;5168;5728;5898;6667;7798;8038;8503;56999;65009
miR-29c-3p	GO:0034332	adherens junction organization	139	7	1.08	6.46	1.09E-04	0.03528388	1307;1946;5728;5796;7414;9448;10979
miR-29c-3p	GO:0038065	collagen-activated signaling pathway	13	3	0.10	29.58	1.25E-04	0.03928271	1285;1287;1288
miR- 665-3p	GO:0006650	glycerophospholipid metabolic process	368	10	1.55	6.47	2.98E-06	0.01751911	391;9110;9365;11145;55650;56261;63874;84962;114882;116986
miR- 665-3p	GO:0006644	phospholipid metabolic process	470	11	1.97	5.57	3.85E-06	0.01751911	391;9110;9365;11145;55650;56261;63874;84962;114882;116986;166929
miR- 665-3p	GO:0044255	cellular lipid metabolic process	1065	16	4.47	3.58	6.48E-06	0.01962179	391;2717;5194;9110;9365;11145;26027;26063;55650;56261;63874;64834;84962;114882;116986;166929
miR- 665-3p	GO:0046486	glycerolipid metabolic process	456	10	1.92	5.22	1.95E-05	0.04424721	391;9110;9365;11145;55650;56261;63874;84962;114882;116986
miR-137-3p	GO:0007628	adult walking behavior	29	4	0.11	34.30	5.25E-06	0.03145353	2043;2743;6323;55074
miR-137-3p	GO:0090659	walking behavior	31	4	0.12	32.09	6.92E-06	0.03145353	2043;2743;6323;55074

Table S10. Statistical details of experiments shown in Fig. 1, S1 and S2.

**Table S10. Extreme subpopulations of addicted and non-addicted mice were obtained from mice trained with chocolate-flavored pellets (Fig. 1, S1 and S2).**				
**Figure number**	**Statistical analysis**	**Factor name**	**Statistic value**	**P-value**
Fig. 1B	Repeated measures ANOVA	FR1 (Sessions 1-6)<br>Group<br>Session<br>Group x Session	 $F(1, 336)= 0.56$<br>$F(5, 336)= 7.63$<br>$F(5, 336)= 0.006$	 n.s.<br>p<0.001<br>n.s.
	Repeated measures ANOVA	FR5 (Sessions 1-92)<br>Group<br>Session<br>Group x Session	 $F(1, 5152)= 812.5$<br>$F(91, 5152)= 2.81$<br>$F(91, 5152)= 0.83$	 p<0.0001<br>p<0.0001<br>n.s.
Fig. 1 C-E	U Mann-Whitney	Standard vs Chocolate<br>Persistence<br>Motivation<br>Compulsive-like behavior	 *U*= 96.50<br>*U*= 47<br>*U*= 137.5	 p<0.05<br>p<0.001<br>n.s.
Fig. 1F	Chi-square	Standard Chow vs Chocolate	χ^2=212.993	p<0.001
Fig. 1G-J	Kolmogorov-Smirnov	Addict vs non-addict<br>Addict<br>Impulsivity<br>Cognitive flexibility<br>Appetitive cue reactivity<br>Aversive cue reactivity<br>Non-addict<br>Impulsivity<br>Cognitive flexibility<br>Appetitive cue reactivity<br>Aversive cue reactivity	 <br>*K-S*= 0.15<br>*K-S*= 0.22<br>*K-S*= 0.19<br>*K-S*= 0.22<br><br>*K-S*= 0.10<br>*K-S*= 0.28<br>*K-S*= 0.22<br>*K-S*= 0.09	 <br>n.s.<br>n.s.<br>n.s.<br>n.s.<br><br>n.s.<br>p<0.001<br>p<0.001<br>n.s.
	U Mann-Whitney or t-test	Addict vs non-addict<br>Impulsivity<br>Cognitive flexibility<br>Appetitive cue reactivity<br>Aversive cue reactivity	 *t*= 4.93<br>*U= 191.5*<br>*U= 139*<br>*t*= 2.23	 p<0.001<br>n.s.<br>p<0.05<br>p<0.05
Fig. 1K-L	Kolmogorov-Smirnov	Addict vs non-addict<br>Total pellet intake non-addict<br>Total pellet intake addict<br>Body weight non-addict<br>Body weight addict	 *K-S*= 0.08<br>*K-S*= 0.20<br>*K-S*= 0.10<br>*K-S*= 0.12	 n.s.<br>n.s.<br>n.s.<br>n.s.
	t-test (equal variances assumed)	Total pellet intake<br><br>Body weight	*t*= 0.06<br><br>*t= 0.05*	n.s.<br><br>n.s.
Fig. S1 C-H	U Mann-Whitney	Standard vs chocolate<br>Early period<br>Persistence of response<br>Motivation<br>Compulsive-like behavior<br>Medium period<br>Persistence of response<br>Motivation<br>Compulsive-like behavior	 <br>*t= 2.36*<br>*U= 117.5*<br>*U= 172*<br><br>*U= 129*<br>*U= 72*<br>*U= 156*	 <br>p<0.05<br>n.s.<br>n.s.<br><br>n.s.<br>p<0.01<br>n.s.
Fig. S1 I-K	Pearson correlation	Persistence and addiction criteria<br>Motivation and addiction criteria<br>Compulsive-like behavior and addiction criteria	*r*= 0.62<br>*r*= 0.64<br>*r*= 0.45	p<0.001<br>p<0.001<br>p<0.001
Fig. S1 L-S	U Mann-Whitney or t-test	Addict vs non-addict<br>Persistence of response<br>Motivation<br>Compulsive-like behavior<br>Pellet intake<br>Body weight<br>Impulsivity	 *U= 17*<br>*t= 7*<br>*U= 4.52*<br>*t= 1.63*<br>*t= 0.75*<br>*t= 5.01*	 p<0.001<br>p<0.001<br>p<0.001<br>n.s.<br>n.s.<br>p<0.001

		Cognitive flexibility<br>Appetitive cue reactivity<br>Aversive cue reactivity	*t= 4.73*<br>*t= 2.43*<br>*U= 3.79*	p<0.001<br>p<0.05<br>p<0.01
Fig. S2 A-B	Repeated measures ANOVA	Appetitive cue reactivity and addiction criteria<br>Group<br>Session<br>Group x Session<br>Aversive cue reactivity and addiction criteria<br>Group<br>Session<br>Group x Session	 <br>*F*(1, 294)= 10.83<br>*F*(5, 294)= 5.29<br>*F*(5, 294)= 2.85<br><br><br>*F*(1, 142)= 6.38<br>*F*(5, 142)= 172.5<br>*F*(5, 142)= 1.48	 <br>p<0.01<br>p<0.001<br>p<0.05<br><br><br>p<0.05<br>p<0.001<br>n.s.
Fig. S2 C-F	Pearson correlation	Impulsivity and addiction criteria<br>Cognitive flexibility and addiction criteria<br>Appetitive cue reactivity g and addiction criteria<br>Aversive cue reactivity and addiction criteria	*r*= 0.50<br>*r*= 0.44<br>*r*= 0.28<br><br>*r*= 0.33	p<0.001<br>p<0.01<br>p<0.05<br><br>p<0.05
Fig. S2 G-J	Repeated measures ANOVA	Impulsivity<br>Group<br>Session<br>Group x Session<br>Cognitive flexibility<br>Group<br>Session<br>Group x Session<br>Appetitive cue reactivity<br>Group<br>Session<br>Group x Session<br>Aversive cue reactivity<br>Group<br>Session<br>Group x Session	 *F*(1, 147)= 4.76<br>*F*(2, 147)= 4.67<br>*F*(2, 147)= 3.40<br><br>*F*(1, 146)= 7.27<br>*F*(2, 146)= 0.98<br>*F*(2, 146)= 1.92<br><br>*F*(1, 147)= 12.57<br>*F*(2, 147)= 1.17<br>*F*(2, 147)= 1.33<br><br>*F*(1, 147)= 15.18<br>*F*(2, 147)= 1.66<br>*F*(2, 147)= 0.11	 p<0.05<br>p<0.05<br>p<0.05<br><br>n.s.<br>n.s.<br>p<0.001<br><br>p<0.001<br>n.s.<br>n.s.<br><br>p<0.001<br>n.s.<br>n.s.
Fig. 1G-J	Kolmogorov-Smirnov	Addict vs non-addict<br>Addict<br>Impulsivity<br>Cognitive flexibility<br>Appetitive cue reactivity<br>Aversive cue reactivity<br>Non-addict<br>Impulsivity<br>Cognitive flexibility<br>Appetitive cue reactivity<br>Aversive cue reactivity	 <br>*K-S*= 0.15<br>*K-S*= 0.22<br>*K-S*= 0.19<br>*K-S*= 0.22<br><br>*K-S*= 0.10<br>*K-S*= 0.28<br>*K-S*= 0.22<br>*K-S*= 0.09	 <br>n.s.<br>n.s.<br>n.s.<br>n.s.<br><br>n.s.<br>p<0.001<br>p<0.001<br>n.s.
	U Mann-Whitney or t-test	Addict vs non-addict<br>Impulsivity<br>Cognitive flexibility<br>Appetitive cue reactivity<br>Aversive cue reactivity	 *t*= 4.93<br>*U= 191.5*<br>*U= 139*<br>*t*= 2.23	 p<0.001<br>n.s.<br>p<0.05<br>p<0.05

Table S11. Statistical details of data shown in Fig. 4.

Table S11. Behavioral results of the three hallmarks of addiction in a human cohort comparing non-addicted ("NA") and addicted ("A") individuals (A).				
**Figure number**	**Statistical analysis**	**Factor name**	**Statistic value**	**P-value**
Fig. 4A-C	Kolmogorov-Smirnov	Men non-addict		
		Persistence	K-S= 0.21	n.s.
		Motivation	K-S= 0.21	n.s.
		Compulsive-like behavior	K-S= 0.53	p<0.001
		Men addict		
		Persistence	*N too small*	
		Motivation	*N too small*	
		Compulsive-like behavior	*N too small*	
		Women non-addict		
		Persistence	K-S= 0.24	p<0.001
		Motivation	K-S= 0.26	p<0.001
		Compulsive-like behavior	K-S= 0.49	p<0.001
		Women addict		
		Persistence	K-S= 0.13	n.s.
		Motivation	K-S= 0.16	n.s.
		Compulsive-like behavior	K-S= 0.21	n.s.
	U Mann-Whitney	Men addict vs non-addict		
		Persistence	U= 3	p<0.05
		Motivation	U= 12.5	n.s.
		Compulsive-like behavior	U= 1	p<0.01
		Women addict vs non-addict		
		Persistence	U= 18.5	p<0.001
		Motivation	U= 36	p<0.001
		Compulsive-like behavior	U= 22	p<0.001
Fig. 4D-F	Pearson correlation	Men		
		Persistence	r= 0.76	p<0.01
		Motivation	r= 0.46	n.s.
		Compulsive-like behavior	r= 0.90	p<0.001
		Women	r= 0.73	p<0.001
		Persistence	r= 0.81	p<0.001
		Motivation	r= 0.65	p<0.001
		Compulsive-like behavior		
Fig. 4G-L	Spearman correlation	Men		
		Persistence of response YFAS 2.0 score vs circulating miR-29c-3p	r= -0.60	p<0.05
		Motivation YFAS 2.0 score vs circulating miR-665-3p	r= -0.60	p<0.05
		Compulsive-like behavior YFAS 2.0 score vs circulating miR-29c-3p	r= -0.59	p<0.05
		YFAS 2.0 score vs circulating miR-29c-3p	r= -0.63	p<0.05
		Sensitivity to reward vs circulating miR-29c-3p	r= -0.68	p<0.01
		Sensitivity to reward vs circulating miR-192-5p	r= -0.23	n.s.
		Women		
		Persistence of response YFAS 2.0 score vs circulating miR-29c-3p	r= 0.06	n.s.
		Motivation YFAS 2.0 score vs circulating miR-665-3p	r= 0.09	n.s.
		Compulsive-like behavior YFAS 2.0 score vs circulating miR-29c-3p	r= 0.08	n.s.
		YFAS 2.0 score vs circulating miR-29c-3p	r= 0.08	n.s.
		Sensitivity to reward vs circulating miR-29c-3p	r= 0.18	n.s.
		Sensitivity to reward vs circulating miR-192-5p	r= 0.44	p<0.05

Table S12. Statistical details of experiments shown in Fig. 5 and Fig. S5.

Table S12. Addiction Criteria, Percentages of addicted animals and phenotypic traits of AAV-anti miR-29c TuD mice.				
**Figure number**	**Statistical analysis**	**Factor name**	**Statistic value**	**P-value**
Fig. 5D	Repeated measures ANOVA	FR1 (Sessions 1-2)<br>Treatment<br>Session<br>Treatment x Session	 $F(1, 29)= 0.26$<br>$F(1, 29)= 9.63$<br>$F(1, 29)= 1.7$	 n.s.<br>p<0.01<br>n.s.
		FR5 (Sessions 3-29)<br>Treatment<br>Session<br>Treatment x Session	 $F(1, 29)= 0.05$<br>$F(26, 754)= 27.85$<br>$F(26, 754)= 0.52$	 n.s.<br>p<0.0001<br>n.s.
Fig. 5 E-G	Kolmogorov-Smirnov	AAV-control TuD<br>Persistence<br>Motivation<br>Compulsive-like behavior<br>AAV-anti miR-29c-3p TuD<br>Persistence<br>Motivation<br>Compulsive-like behavior	 *K-S*= 0.18<br>*K-S*= 0.17<br>*K-S*= 0.19<br><br>*K-S*= 0.18<br>*K-S*= 0.22<br>*K-S*= 0.13	 n.s.<br>n.s.<br>n.s.<br><br>n.s.<br>n.s.<br>n.s.
	t-test (equal variances not assumed)	Persistence	*t*= -2,68	p<0.05
	t-test (equal variances assumed)	Motivation<br>Compulsive-like behavior	*t*= -1.27<br>*t*= 0.18	n.s.<br>n.s.
Fig. 5H	Chi square	Treatment	χ^2== 10.56	p<0.001
Fig. 5I-K	Pearson correlation	AAV-control TuD<br>Persistence and addiction criteria<br>Motivation and addiction criteria<br>Compulsive-like behavior and addiction criteria	 *r*= 0.52<br>*r*= 0.59<br>*r*= 0.56	 p<0.05<br>p<0.01<br>p<0.05
	Pearson correlation	AAV-anti miR-29c-3p TuD<br>Persistence and addiction criteria<br>Motivation and addiction criteria<br>Compulsive-like behavior and addiction criteria	 *r*= 0.73<br>*r*= 0.72<br>*r*= 0.60	 p<0.01<br>p<0.01<br>p<0.05
Fig. 5L-M	Kolmogorov-Smirnov	AAV-anti miR-29c-3p TuD<br>Total pellet intake Control<br>Total pellet intake AAV-anti miR-29c-3p TuD<br>Body weigh Control<br>Body weigh AAV-anti miR-29c-3p TuD	 *K-S*= 0.10<br>*K-S*= 0.23<br><br>*K-S*= 0.14<br>*K-S*= 0.31	 n.s.<br>n.s.<br><br>n.s.<br>p<0.01
	t-test (equal variances assumed)<br>U Mann-Whitney	Total pellet intake<br><br>Body weight	*t*= 0.38<br><br>*U*= 85	n.s.<br><br>n.s.
Fig. S5A	Kolmogorov-Smirnov	AAV-control TuD<br>Impulsivity<br>AAV-anti miR-29c-3p TuD<br>Impulsivity	 *K-S*= 0.15<br><br>*K-S*= 0.14	 n.s.<br><br>n.s.
	t-test (equal variances assumed)	Impulsivity	*t*= -1.09	n.s.
Fig. S5B	U Mann-Whitney	AAV-control TuD NA vs. A<br>Basal Active<br>Basal Inactive<br>Reversal Active (before inactive)<br>Reversal Inactive (before active)<br>AAV-anti miR-29c-3p TuD NA vs. A<br>Basal Active<br>Basal Inactive<br>Reversal Active (before inactive)<br>Reversal Inactive (before active)	 *U*= 17<br>*U*= 10<br>*U*= 10<br>*U*= 10<br><br>*U*= 8<br>*U*= 17<br>*U*= 8<br>*U*= 9	 n.s.<br>n.s.<br>n.s.<br>n.s.<br><br>n.s.<br>n.s.<br>n.s.<br>n.s.
Fig. S5C-D	Kolmogorov-Smirnov	AAV-control TuD<br>Appetitive cue reactivity<br>Aversive cue reactivity<br>AAV-anti miR-29c-3p TuD	 *K-S*= 0.16<br>*K-S*= 0.11	 n.s.<br>n.s.

		Appetitive cue reactivity<br>Aversive cue reactivity	*K-S*= 0.17<br>*K-S*= 0.15	n.s.<br>n.s.
	t-test (equal variances not assumed)	Appetitive cue reactivity	*t*= -1.41	n.s.
	t-test (equal variances assumed)	Aversive cue reactivity	*t*= -1.14	n.s.
Fig. S5E-H	Pearson correlation	AAV-control TuD<br>Impulsivity and addiction criteria<br>Cognitive flexibility and addiction criteria<br>Appetitive cue reactivity and addiction criteria<br>Aversive cue reactivity and addiction criteria	 *r*= 0.52<br>*r*= 0.58<br>*r*= 0.21<br>*r*= 0.25	 p<0.05<br>p<0.01<br>n.s.<br>n.s.
	Pearson correlation	AAV-anti miR-29c-3p TuD<br>Impulsivity and addiction criteria<br>Cognitive flexibility and addiction criteria<br>Appetitive cue reactivity and addiction criteria<br>Aversive cue reactivity and addiction criteria	 *r*= 0.12<br>*r*= 0.44<br>*r*= 0.26<br>*r*= 0.07	 n.s.<br>n.s.<br>n.s.<br>n.s.
Fig. S5I-L	U Mann-Whitney	AAV-control TuD NA vs A<br>Impulsivity<br>Persistence<br>Motivation<br>Compulsive-like behavior<br>AAV-anti miR-29c-3p TuD NA vs A<br>Impulsivity<br>Persistence<br>Motivation<br>Compulsive-like behavior	 *U*= 11<br>*U*= 2<br>*U*= 9.5<br>*U*= 12.5<br><br>*U*= 12<br>*U*= 3<br>*U*= 9<br>*U*= 10	 n.s.<br>p<0.01<br>n.s.<br>n.s.<br><br>n.s.<br>p<0.01<br>n.s.<br>n.s.

Table S13. Statistical details of experiments shown in Fig. 6, Fig. S4 and Fig. S6.

**Table S13. Addiction Criteria, Percentages of addicted animals and phenotypic traits of AAV-anti miR-665-3p TuD mice.**				
**Figure number**	**Statistical analysis**	**Factor name**	**Statistic value**	**P-value**
Fig. 6B	Repeated measures ANOVA	FR1 (Sessions 1-2)<br>Treatment<br>Session<br>Treatment x Session	 F(1, 35)= 0.65<br>F(1, 35)= 30.46<br>F(1, 35)= 0.01	 n.s.<br>p<0.0001<br>n.s.
		FR5 (Sessions 3-29)<br>Treatment<br>Session<br>Treatment x Session	 F(1, 35)= 0.09<br>F(26, 910)= 23.5<br>F(26, 910)= 0.49	 n.s.<br>p<0.0001<br>n.s.
Fig. 6C-E	Kolmogorov-Smirnov	AAV-control TuD<br>Persistence<br>Motivation<br>Compulsive-like behavior<br>AAV-anti miR-6653p TuD<br>Persistence<br>Motivation<br>Compulsive-like behavior	 K-S= 0.24<br>K-S= 0.21<br>K-S= 0.17<br><br>K-S= 0.25<br>K-S= 0.21<br>K-S= 0.17	 p<0.01<br>p<0.05<br>n.s.<br><br>p<0.01<br>p<0.05<br>n.s.
	U Mann-Whitney	Persistence<br>Motivation	U= 142.5<br>U= 162	n.s.<br>n.s.
	t-test (equal variances not assumed)	Compulsive-like behavior	t= -2.46	p<0.05
Fig. 6F	Chi square	Treatment	χ^2== 5.57	p<0.05
Fig. 6G-I	Pearson correlation	AAV-control TuD<br>Persistence and addiction criteria<br>Motivation and addiction criteria<br>Compulsive-like behavior and addiction criteria	 r= 0.62<br>r= 0.72<br>r= 0.37	 p<0.01<br>p<0.001<br>n.s.
	Pearson correlation	AAV-anti miR-665-3p TuD<br>Persistence and addiction criteria<br>Motivation and addiction criteria<br>Compulsive-like behavior and addiction criteria	 r= 0.77<br>r= 0.71<br>r= 0.63	 p<0.001<br>p<0.001<br>p<0.01
Fig. 6J-K	Kolmogorov-Smirnov	AAV-anti miR-665c-3p TuD<br>Total pellet intake Control<br>Total pellet intake AAV-anti miR-665-3p TuD<br>Body weigh Control<br>Body weigh AAV-anti miR-665-3p TuD	 K-S= 0.13<br>K-S= 0.18<br><br>K-S= 0.17<br>K-S= 0.19	 n.s.<br>n.s.<br><br>n.s.<br>n.s.
	t-test (equal variances assumed)	Total pellet intake<br>Body weight	t= 0.71<br>t= 0.097	n.s.<br>n.s.
Fig. S4	Kolmogorov-Smirnov	Ncam1<br>AAV-control TuD<br>non-addict<br>Addict<br>AAV-anti-miR-665-3p TuD<br>non-addict<br>Addict<br><br>Rbfox1<br>AAV-control TuD<br>non-addict<br>Addict<br>AAV-anti-miR-665-3p TuD<br>non-addict<br>Addict	 <br>K-S= 0.15<br>N too small<br><br>K-S= 0.21<br>K-S= 0.29<br><br><br><br>K-S= 0.23<br>N too small<br><br>K-S= 0.10<br>K-S= 0.21	 <br>n.s.<br><br><br>n.s.<br>n.s.<br><br><br><br>p<0.05<br><br><br>n.s.<br>n.s.
		Ncam1<br>AAV-control TuD<br>non-addict vs addict<br><br>AAV-anti-miR-665-3p TuD<br>non-addict vs Addict<br>AAV-control TuD vs AAV-anti-miR-665-3p TuD	 <br>U= 0.15<br><br><br>t= 2.14<br>U= 19	 <br>p<0.05<br><br><br>p<0.001<br>p<0.001

	U Mann-Whitney<br>or t-test	 Rbfox1<br>AAV-control TuD<br>non-addict vs Addict<br><br>AAV-anti-miR-665-3p TuD<br>non-addict vs Addict<br>AAV-control TuD vs AAV-anti-miR-665-3p TuD	 <br><br>U= 19.50<br><br><br>t= 2.60<br>U= 19.50	 <br><br>n.s.<br><br><br>p<0.05<br>p<0.001
Fig. S6A	Kolmogorov-Smirnov	AAV-control TuD<br>Impulsivity<br>AAV-anti miR-665-3p TuD<br>Impulsivity	 K-S= 0.23<br><br>K-S= 0.15	 p<0.05<br><br>n.s.
	U Mann-Whitney	Impulsivity	U= 141	n.s.
Fig. S6B	U Mann-Whitney	AAV-control TuD NA vs. A<br>Basal Active<br>Basal Inactive<br>Reversal Active (before inactive)<br>Reversal Inactive (before active)<br>AAV-anti miR-665-3p TuD NA vs. A<br>Basal Active<br>Basal Inactive<br>Reversal Active (before inactive)<br>Reversal Inactive (before active)	 U= 7<br>U= 18.5<br>U= 14<br>U= 3<br><br>U= 23<br>U= 31<br>U= 29<br>U= 24	 n.s.<br>n.s.<br>n.s.<br>p<0.05<br><br>p<0.05<br>n.s.<br>n.s.<br>n.s.
Fig. S6C-D	Kolmogorov-Smirnov	AAV-control TuD<br>Appetitive cue reactivity<br>Aversive cue reactivity<br>AAV-anti miR-665-3p TuD<br>Appetitive cue reactivity<br>Aversive cue reactivity	 K-S= 0.16<br>K-S= 0.13<br><br>K-S= 0.16<br>K-S= 0.20	 n.s.<br>n.s.<br><br>n.s.<br>n.s
	t-test (equal variances assumed)	Appetitive cue reactivity<br>Aversive cue reactivity	t= -0.28<br>t= -0.67	n.s.<br>n.s.
Fig. S6E-H	Pearson correlation	AAV-control TuD<br>Impulsivity and addiction criteria<br>Cognitive flexibility and addiction criteria<br>Appetitive cue reactivity and addiction criteria<br>Aversive cue reactivity and addiction criteria	 r= 0.33<br>r= 0.73<br>r= 0.60<br><br>r= 0.24	 n.s.<br>n.s.<br>n.s.<br><br>n.s.
	Pearson correlation	AAV-anti miR-665-3p TuD<br>Impulsivity and addiction criteria<br>Cognitive flexibility and addiction criteria<br>Appetitive cue reactivity and addiction criteria<br>Aversive cue reactivity and addiction criteria	 r= 0.42<br>r= 0.55<br>r= 0.34<br><br>r= 0.68	 n.s.<br>p<0.05<br>n.s.<br><br>p<0.01
Fig. S7I-L	U Mann-Whitney	AAV-control TuD NA vs A<br>Impulsivity<br>Persistence<br>Motivation<br>Compulsive-like behavior<br>AAV-anti miR-665-3p TuD NA vs A<br>Impulsivity<br>Persistence<br>Motivation<br>Compulsive-like behavior	 U= 8<br>U= 6<br>U= 2.5<br>U= 15<br><br>U= 21<br>U= 1<br>U= 9.5<br>U= 19.5	 n.s.<br>n.s.<br>p<0.01<br>n.s.<br><br>n.s.<br>p<0.001<br>p<0.01<br>n.s.

Table S14. Statistical details of experiments shown in Fig. 7 and Fig. S7.

**Table S14. Addiction Criteria, Percentages of addicted animals and phenotypic traits of AAV-anti miR-137-3p TuD mice.**				
**Figure number**	**Statistical analysis**	**Factor name**	**Statistic value**	**P-value**
Fig. 7B	Repeated measures ANOVA	FR1 (Sessions 1-2)<br>Treatment<br>Session<br>Treatment x Session	 $F(1, 31)= 0.36$<br>$F(1, 31)= 13.52$<br>$F(1, 31)= 0.91$	 n.s.<br>p<0.001<br>n.s.
		FR5 (Sessions 3-29)<br>Treatment<br>Session<br>Treatment x Session	 $F(1, 31)= 1.91$<br>$F(26, 806)= 26.23$<br>$F(26, 806)= 1.11$	 n.s.<br>p<0.0001<br>n.s.
Fig. 7C-E	Kolmogorov-Smirnov	AAV-control TuD<br>Persistence<br>Motivation<br>Compulsive-like behavior<br>AAV-anti miR-137-3p TuD<br>Persistence<br>Motivation<br>Compulsive-like behavior	 *K-S*= 0.19<br>*K-S*= 0.22<br>*K-S*= 0.17<br><br>*K-S*= 0.11<br>*K-S*= 0.33<br>*K-S*= 0.16	 n.s<br>p<0.05<br>n.s.<br><br>n.s.<br>p<0.001<br>n.s.
	t-test (equal variances assumed)	Persistence	$t= -0.94$	n.s.
	U Mann-Whitney	Motivation	$U= 129.5$	n.s.
	t-test (equal variances assumed)	Compulsive-like behavior	$t= -0.40$	n.s.
Fig. 7F	Chi square	Treatment	χ^2== 0.26	n.s.
Fig. 7G-I	Pearson correlation	AAV-control TuD<br>Persistence and addiction criteria<br>Motivation and addiction criteria<br>Compulsive-like behavior and addiction criteria	 $r= 0.64$<br>$r= 0.78$<br>$r= 0.83$	 p<0.01<br>p<0.001<br>p<0.0001
	Pearson correlation	AAV-anti miR-137-3p TuD<br>Persistence and addiction criteria<br>Motivation and addiction criteria<br>Compulsive-like behavior and addiction criteria	 $r= 0.82$<br>$r= 0.62$<br>$r= 0.60$	 p<0.0001<br>p<0.01<br>p<0.05
Fig. 7J-K	Kolmogorov-Smirnov	AAV-anti miR-137-3p TuD<br>Total pellet intake Control<br>Total pellet intake AAV-anti miR-137-3p TuD<br>Body weigh Control<br>Body weigh AAV-anti miR-137-3p TuD	 *K-S*= 0.19<br>*K-S*= 0.13<br>*K-S*= 0.18<br>*K-S*= 0.25	 n.s.<br>n.s.<br>n.s.<br>n.s.
	t-test (equal variances assumed)	Total pellet intake<br>Body weight	$t= 0.18$<br>$t= 1.38$	n.s.<br>n.s.
Fig. S7A	Kolmogorov-Smirnov	AAV-control TuD<br>Impulsivity<br>AAV-anti miR-137-3p TuD<br>Impulsivity	 *K-S*= 0.20<br><br>*K-S*= 0.33	 n.s.<br><br>p<0.05
	U Mann-Whitney	Impulsivity	$U= 129.5$	n.s.
Fig. S7B	U Mann-Whitney	AAV-control TuD NA vs. A<br>Basal Active<br>Basal Inactive<br>Reversal Active (before inactive)<br>Reversal Inactive (before active)<br>AAV-anti miR-137-3p TuD NA vs. A<br>Basal Active<br>Basal Inactive<br>Reversal Active (before inactive)<br>Reversal Inactive (before active)	 $U= 14$<br>$U= 13.5$<br>$U= 13$<br>$U= 15$<br><br>$U= 22$<br>$U= 15.5$<br>$U= 20$<br>$U= 18$	 n.s.<br>n.s.<br>n.s.<br>n.s.<br><br>n.s.<br>n.s.<br>n.s.<br>n.s.
Fig. S7C-D	Kolmogorov-Smirnov	AAV-control TuD<br>Appetitive cue reactivity<br>Aversive cue reactivity	 *K-S*= 0.26<br>*K-S*= 0.13	 p<0.01<br>n.s.

		AAV-anti miR-137-3p TuD<br>Appetitive cue reactivity<br>Aversive cue reactivity	 *K-S*= 0.11<br>*K-S*= 0.20	 n.s.<br>n.s.
	U Mann-Whitney	Appetitive cue reactivity	*U*= 117500	n.s.
	t-test (equal variances assumed)	Aversive cue reactivity	*t*= -1.21	n.s.
Fig. S7E-H	Pearson correlation	AAV-control TuD<br>Impulsivity and addiction criteria<br>Cognitive flexibility and addiction criteria<br>Appetitive cue reactivity and addiction criteria<br>Aversive cue reactivity and addiction criteria	 *r*= 0.45<br>*r*= 0.45<br>*r*= 0.13<br>*r*= 0.29	 n.s.<br>n.s.<br>n.s.<br>n.s.
	Pearson correlation	AAV-anti miR-137-3p TuD<br>Impulsivity and addiction criteria<br>Cognitive flexibility and addiction criteria<br>Appetitive cue reactivity and addiction criteria<br>Aversive cue reactivity and addiction criteria	 *r*= 0.37<br>*r*= 0.46<br>*r*= 0.13<br>*r*= 0.32	 n.s.<br>n.s.<br>n.s.<br>n.s.
Fig. S7I-L	U Mann-Whitney	AAV-control TuD NA vs A<br>Impulsivity<br>Persistence<br>Motivation<br>Compulsive-like behavior<br>AAV-anti miR-137-3p TuD NA vs A<br>Impulsivity<br>Persistence<br>Motivation<br>Compulsive-like behavior	 *U*= 9<br>*U*= 11.5<br>*U*= 3.5<br>*U*= 1<br><br>*U*= 23<br>*U*= 2.5<br>*U*= 8<br>*U*= 17.5	 n.s.<br>n.s.<br>p<0.05<br>p<0.01<br><br>n.s.<br>p<0.01<br>p<0.05<br>n.s.
